# Supplementary material for: First-in-human phase Ia study of the PI3Kα inhibitor CYH33 in patients with solid tumors
Source: Nat Commun. 2022 Nov 16;13:7012. doi: 10.1038/s41467-022-34782-9 (PMC9669016; doi:10.1038/s41467-022-34782-9)
Supplement: Supplementary file 1 — Supplementary Information [file 41467_2022_34782_MOESM1_ESM.pdf]

Supplementary Information File

Supplementary Table 1. Summary of CYH33-specific PK parameters on Cycle 1 Day 28

Supplementary Table 2. Summary of I27 PK parameters on Cycle 1 Day 1

Supplementary Table 3. Summary of I27 PK parameters on Cycle 1 Day 28

Supplementary Fig. 1 Waterfall plot of maximum percent change from baseline in the sum of target lesion diameters in the response evaluable populations by local investigators/radiologists' review (n=42)

Supplementary Fig. 2 Swimmer plot of duration of treatment and response at each visit in the response evaluable populations (n=42)

Supplementary Note 1. Study Protocol

Supplementary Note 2. Statistical Analysis Plan

Supplementary Table 1. Summary of CYH33-specific PK parameters on Cycle 1 Day 28

Dose	T_{max}, h, median (min-max)	C_{max}, ng/mL	C_{min}, ng/mL	$AUC_{0\text{-}24h}$, h*ng/mL	CL_{ss}/F, L/h	Rac_AUC	Rac_C_{max}
1 mg (n=1)	4	4.8	0	51.6	19.4	2.0	1.0
5 mg (n=1)	1	56.2	15.1	582.2	8.6	2.2	2.3
10 mg (n=2)	3 (2–4)	78.9 (3)	33.4 (34)	1234.5 (10)	8.2 (10)	2.0 (14)	1.8 (4)
20 mg (n=6)	3 (1–4)	154.0 (32)	80.8 (27)	2627.0 (28)	8.1 (25)	4.2 (68)	3.3 (70)
30 mg (n=9)	3 (1–12)	187.3 (38)	111.4 (44)	3530.0 (37)	11.0 (77)	2.9 (37)	1.9 (34)
40mg (n=7)	4 (1–6)	224.4 (35)	126.5 (42)	3942.4 (36)	11.8 (48)	4.3 (42)	3.2 (41)
60 mg (n=2)	2.5 (1–4)	220.5 (19)	133.2 (45)	4008.8 (34)	15.9 (34)	3.2 (2)	2.2 (3)

All data are summarized as mean, CV% unless indicated. $AUC_{0\text{-}24}$, area under the plasma concentration-time curve from time 0 to 24 hours; C_{max}, maximum plasma drug concentration; C_{min}, minimum drug concentration between doses; CL_{ss}/F, apparent total clearance of the drug from plasma after oral administration (at steady state); RAC_AUC, accumulation ratio based on AUC; RAC_C_{max}, accumulation ratio based on C_{max}.

Supplementary Table 2. Summary of I27 PK parameters on Cycle 1 Day 1 [a]

Dose	T_{max}, h, median (min-max)	C_{max}, ng/mL	AUC_{0-24}, h*ng/mL	$AUC_{0-\infty}$, h*ng/mL	$t_{1/2}$, h	V/F, L	CL/F, L/h
1 mg (n=1)	8.0	1.4	29.1	90.9	NA	NA	NA
5 mg (n=1)	8.0	10.2	207.4	707.1	NA	NA	NA
10 mg (n=2)	10 (8.0-12)	7.7 (9)	160.9 (13)	540.0 (23)	27.8 (NA)	887.1 (NA)	22.1 (NA)
20 mg (n=5)	23.8 (8.0-24)	14.7 (58)	298.4 (60)	1808.9 (NA)	NA	NA	NA
30 mg (n=12)	23.9 (6.0-23.9)	18.3 (31)	354.2 (34)	NA	NA	NA	NA
40mg (n=3)	23.9 (8.0-23.9)	22.7 (49)	424.0 (44)	NA	NA	NA	NA
60 mg (n=4)	15.9 (4.0-23.9)	30.0 (42)	559.0 (41)	NA	NA	NA	NA

All data are summarized as mean, CV% unless indicated. [a]AUC_{0-inf}, $t_{1/2}$, V/F and CL/F were only calculated when $\%AUC_{ex}$ was <30%. $AUC_{0-24,}$ area under the plasma concentration-time curve from time 0 to 24 hours; $AUC_{0-\infty}$, area under the plasma concentration-time curve from time 0 to infinity; C_{max}, maximum plasma drug concentration; CL/F, apparent total clearance of the drug from plasma after oral administration; $t_{1/2}$, terminal elimination half-life; T_{max}, maximum plasma drug concentration; V/F, apparent volume of distribution.

Supplementary Table 3. Summary of I27 PK parameters on Cycle 1 Day 28

Dose	T_{max}, h, median (min-max)	C_{max}, ng/mL	C_{min}, ng/mL	AUC_{0-24}, h*ng/mL	CL_{ss}/F, L/h	Rac_AUC	Rac_C_{max}
1 mg (n=1)	4.0	3.2	2.0	57.5	17.4	2.0	2.2
5 mg (n=1)	2.0	35.5	28.1	759.4	6.6	3.7	3.5
10 mg (n=2)	4.0 (4.0-4.0)	29.3 (16)	19.9 (28)	557.6 (14)	18.1 (14)	3.6 (26)	3.9 (25)
20 mg (n=6)	4.0 (3.0-6.0)	85.4 (55)	66.7 (53)	1827.3 (55)	13.6 (45)	6.7 (69)	6.0 (62)
30 mg (n=9)	6.0 (3-23.9)	63.8 (49)	47.3 (50)	1348.7 (51)	43.4 (146)	3.7 (39)	3.4 (36)
40mg (n=7)	4.0 (0.0-4.0)	104.6 (26)	79.5 (39)	2201.0 (31)	20.5 (45)	6.2 (32)	5.6 (38)
60 mg (n=2)	6.0 (6.0-6.0)	109.3 (49)	88.9 (56)	2273.2 (54)	30.9 (54)	5.1 (42)	4.8 (34)

All data are summarized as mean, CV% unless indicated. AUC_{0-24}, area under the plasma concentration-time curve from time 0 to 24 hours; C_{max}, maximum plasma drug concentration; C_{min}, minimum drug concentration between doses; CL_{ss}/F, apparent total clearance of the drug from plasma after oral administration (at steady state); RAC_AUC, accumulation ratio based on AUC; RAC_C_{max}, accumulation ratio based on C_{max}.

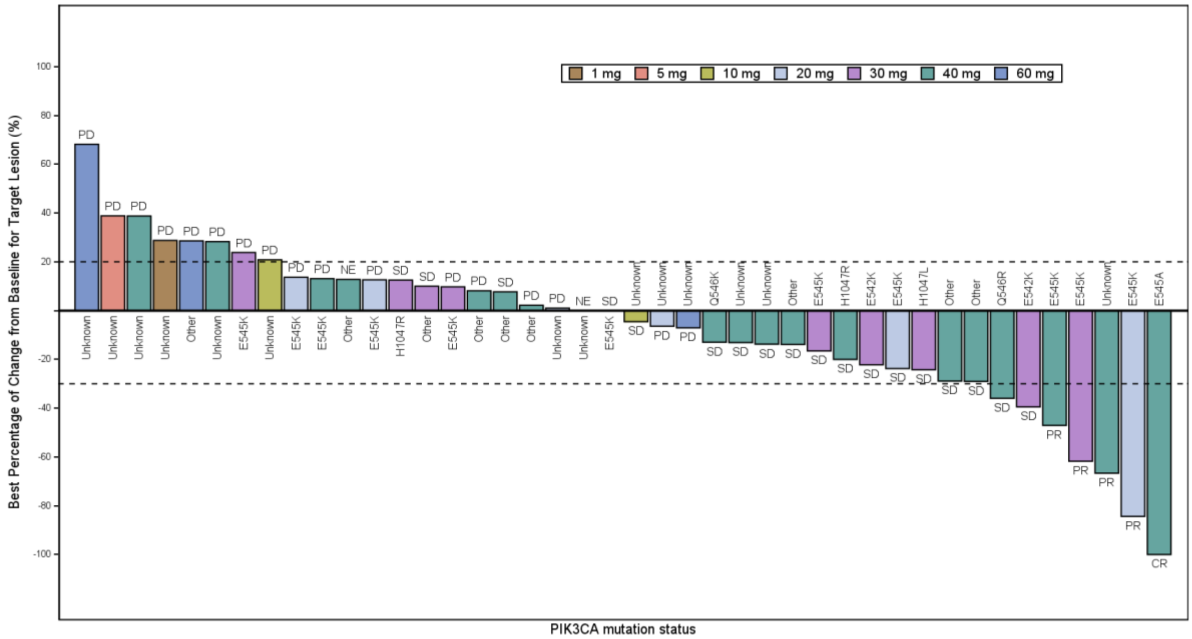

Supplementary Fig. 1 Waterfall plot of maximum percent change from baseline in the sum of target lesion diameters in the response evaluable populations by local investigators/radiologists' review (n=42). The best overall responses and *PIK3CA* mutation status were shown on the upper and lower of the histograms, respectively. Two best responses marked as NE represented SD lasting less than 5 weeks. CR, complete response; PD, progressive disease; PR, partial response; SD, stable disease.

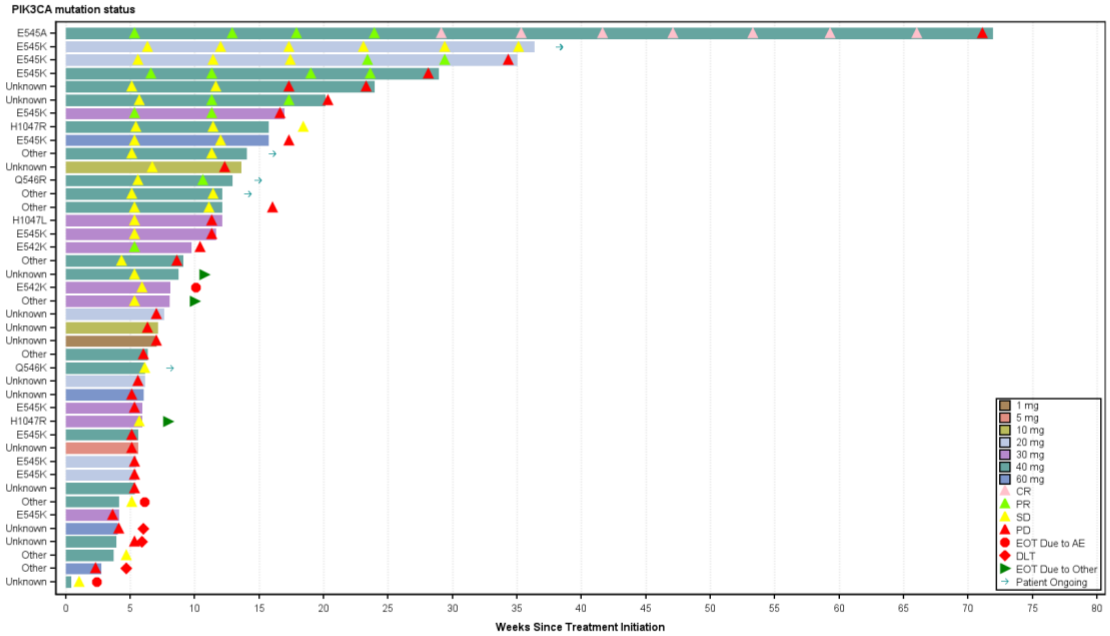

Supplementary Fig. 2 Swimmer plot of duration of treatment and response at each visit in the response evaluable populations (n=42). The vertical scale marked the *PIK3CA* mutation status for each patient. CR, complete response; DLT, dose-limiting toxicity; EOT, end of treatment; PD, progressive disease; PR, partial response; QD, once daily; SD, stable disease. Other represented alterations in *PIK3CA* outside of exon9 and exon20.

Supplementary Note 1

Clinical Study Protocol

**Protocol title:**	A multi-center, open-label, single-arm dose escalation and expansion Phase I clinical study to evaluate the safety, tolerability, pharmacokinetic characteristics and preliminary efficacy of CYH33 in patients with advanced solid tumors
**Study protocol No.**	CYH33-101
**Study drug:**	CYH33

**Co-investigators:**	Professor Xu Ruihua

**Protocol version:**	5.0
**Date:**	September 24, 2019

Confidentiality Statement

All the information contained in this protocol is confidential and proprietary to Shanghai Haihe Biopharma Co., Ltd.

Protocol version	Date finalized	Country involved	Revisions
5.0	September 24, 2019	China	• **Study population:**<br>▪ For the Phase Ia dose expansion part: the study population was revised from patients with *PIK3CA* gene abnormalities (mutations or amplifications) to patients with PIK3CA-mutated advanced solid tumors who have failed or cannot tolerate standard treatment or currently have no standard treatment.<br>• **Inclusion criteria:**<br>▪ Molecular screening criteria were revised from providing evidence of *PIK3CA* gene abnormality (mutation or amplification) to providing evidence of *PIK3CA* gene mutation.<br>▪ For the patients with available *PIK3CA* gene mutation diagnosis report provided by medical/diagnostic facilities, "the tumor tissue specimens must be sent to the designated screening laboratory for confirmation before the initiation of treatment with study drug" was revised to "the clinical screening and subsequent study phases can be entered, while sending tumor tissue specimens (at least 10 unstained tumor tissue sections; or/and formalin-fixed paraffin-embedded pathological tumor tissue or/and fresh tumor tissue) and copies of the corresponding pathology reports and genetic diagnosis reports to the designated screening laboratory for confirmation.<br>• **Dose adjustment:** Delete "Each dose reduction will be reduced to the previous dose group"<br>• **Principles for handling of adverse events during the study:**<br>▪ "Each dose reduction will be reduced to the previous dose group" was deleted and revised to "The dose reduction is determined by the investigator and sponsor based on the

Protocol version	Date finalized	Country involved	Revisions
			severity of AE and available safety and efficacy data".<br>• **The schematic diagram of dose escalation and expansion design was updated accordingly.**<br>• **Study procedures section was updated accordingly.**
4.0	June 26, 2019	China	• The **protocol title** was changed to "A multi-center, open-label, single-arm dose escalation and expansion Phase I clinical study to evaluate the safety, tolerability, pharmacokinetic characteristics and preliminary efficacy of CYH33 in patients with advanced solid tumors."<br>• **Study background:** The rationale for mTPI2 study design and subject population selection was updated.<br>• **Study objectives and endpoints:** The primary study objectives and endpoints, secondary study objectives and endpoints, and exploratory study objectives and endpoints were updated. The definition of study endpoints, including MTD, RP2D and CBR, were updated.<br>• **Study plan:**<br>1) Study phase: Phase Ia dose escalation, Phase Ia dose expansion and Phase Ib.<br>2) Changed the study design:<br>▪ Dose expansion study was additionally conducted with dose escalation in Phase Ia.<br>▪ Phase Ia dose escalation was changed from rapid titration phase followed by conventional "3 + 3" design escalation phase to rapid titration phase followed by adaptive dose escalation phase guided by mTPI2 design.<br>[redacted]

Protocol version	Date finalized	Country involved	Revisions
			[redacted]<br>▪ In light of the safety, efficacy, and PK/PD data from Phase Ia study, SMC will determine the recommended Phase II dose (RP2D), which may be adjusted during Phase Ib study if necessary.<br>3) Changes in the study population:<br>▪ Phase Ia dose escalation part: patients with advanced solid tumors who have failed or cannot tolerate standard treatment or currently have no standard treatment.<br>▪ Phase Ia dose expansion part: patients with advanced solid tumors with *PIK3CA* gene mutations who have failed or cannot tolerate standard treatment or currently have no standard treatment.<br>[redacted]<br>4) Updates of the criteria for dose-limiting toxicity (DLT).<br>5) Updates of the principles for dose modification.<br>6) Updates of the definition of “End of study”.<br>• **Selection of subjects:**<br>1) Updates of inclusion criteria:<br>▪ Addition of molecular screening criteria: Patients sign the informed consent form and provide evidence of *PIK3CA* gene abnormality (mutation or amplification). This is applicable to the Phase Ia dose expansion part and Phase Ib.

Protocol version	Date finalized	Country involved	Revisions
			▪ Updates of clinical screening criteria.<br>2) Updates of exclusion criteria:<br>• **Study drug and study methods:** The criteria for CYH33 withdrawal, resumption, dose reduction and permanent withdrawal were updated.<br>• Corresponding updates of **study procedures** and assessments.<br>• Corresponding updates of **statistics** and statistical analyses.<br>• **Appendix:** Phase Ia and Phase Ib assessment list, PK blood sampling time points and blood sampling volumes, Cockcroft-Gault equation were updated.

Protocol version	Date finalized	Country involved	Revisions
2.0	September 18, 2016	China	• Changes to the protocol title:<br>▪ Patients enrolled in Phase Ib were changed to those with advanced squamous cell carcinoma of esophagus and gastroesophageal junction<br>• Updates of the study background<br>• Updates of nonclinical PD, PK, and toxicological data<br>• Updates of clinical benefit/risk profile of study drug<br>• Updates of study rationale<br>• Changes to the study design<br>▪ The observation phase after single dose was changed to 7 days, which was adjusted based on PK ($t_{1/2}$) data<br>▪ The observation phase for DLT was 28 days (35 days for patients who received single dose)<br>▪ One subject was enrolled in the initial dose, 1 mg group, and at least 2 males or 2 females were required in MTD group (6 subjects) to analyze the gender differences in PK parameters.<br>▪ In principle, dose up-regulation was not allowed in individual patients during the study.<br>• Updates of DLT definition<br>▪ Grade 4 neutropenia (neutrophil count < 0.5×10^9/L);<br>▪ Grade 3 thrombocytopenia (platelet count < 50×10^9/L), and failure to return to ≤ grade 2 or baseline level within 7 days after the study drug is suspended;<br>▪ Grade 3 neutropenia with fever (neutrophil count < 1.0×10^9/L, fever ≥ 38.5°C)<br>▪ Other grade 4 hematological toxicities<br>▪ Grade 3 hyperglycemia (fasting blood glucose > 13.9-27.8 mmol/L or 250 mg/dL) or asymptomatic Grade 4 hyperglycemia (fasting blood glucose > 27.8 mmol/L or 500 mg/dL), which does not improve within ≤ 7 days after appropriate anti-diabetic treatment. Or symptomatic Grade 4 hyperglycemia (fasting blood glucose > 27.8 mmol/L or 500 mg/dL)<br>• Update and clarification of inclusion/exclusion criteria:<br>▪ Platelets ≥ 90×10^9/L<br>▪ Fasting blood glucose ≤ 126 mg/dL or ≤ 7.0 mmol/L;<br>▪ HbA1c ≤ 7.5%

Protocol version	Date finalized	Country involved	Revisions
			▪ The time interval between the last dose, if treated with PD-1 or PDL-L1, and the first dose of study drug must be greater than 90 days<br>▪ Dysphagia, or ulcerative colitis, Crohn's disease; Or patients who have undergone small bowel resection (more than one-third of the small intestine has been resected); Or uncontrollable nausea, vomiting, diarrhea, malabsorption syndrome and other diseases which may significantly affect the administration or absorption of CYH33. Patients with a history of digestive tract perforation or obstruction may not be enrolled<br>• Updates of PK blood sampling sites<br>▪ C1D15: pre-dose (within 15 min before administration);<br>▪ C1D28: pre-dose (within 15 min before administration), and at 0.5, 1, 2, 4, 8 and 12 h post-dose, respectively;<br>▪ C2D1: at 24 h post-dose.<br>• Updates of response assessment cycle<br>▪ Tumor evaluation is conducted according to RECIST Version 1.1 every 6 weeks until disease progression, unacceptable toxicity, death, or discontinuation study by investigator's decision or consent withdrawal of patients.<br>• Updates of tumor markers and pharmacodynamic parameters<br>▪ To evaluate the molecular biological alterations in markers related to PI3K signaling in tumor specimens (e.g. *PIK3CA* alteration, PTEN alteration, KRAS and BRAF mutation, AKT alteration, etc.)<br>▪ To examine pharmacodynamic parameters such as blood glucose, Akt phosphorylation, S6k1 and rpS6 phosphorylation over time.

Table of Contents

List of Tables ... 11

List of Figures ... 11

Protocol Approval Signature Page ... 12

Protocol Signature Page ... 13

Protocol Synopsis ... 15

Abbreviations ... 33

1 Study Background ... 36

1.1 Medical Backgroung ... 36
1.2 Introduction to study drug ... 37
1.2.1 Results of Nonclinical Pharmacodynamic Study ... 38
1.2.2 Nonclinical Pharmacokinetics ... 40
1.2.3 Results of Nonclinical Toxicological Study ... 41
1.3 Clinical Benefit/Risk Analysis of Study Drug ... 41
1.4 Rationale of the Clinical Study ... 42

2 Study Objectives and Endpoints ... 45

2.1 Study Objectives ... 45
2.2 Study endpoints ... 45
2.3 Definitions of Study Endpoints ... 47

3 Study Plan ... 48

3.1 Overall Study Design ... 48
3.2 Phase Ia Dose Escalation Part ... 52
3.2.1 Single Dose Phase ... 52
3.2.2 Continuous Dose Phase ... 52
3.2.3 Dose-finding Principle ... 52
3.2.4 Dose Escalation Adjustment ... 53
3.2.5 Intra-patient Dose Escalation ... 53
3.2.6 Definition and Evaluation of Dose-Limiting Toxicity (DLT) ... 53
3.2.7 Requirements for Enrollment Interval within One Dose Group ... 54
3.2.8 Follow-up of DLT ... 55
3.3 Phase Ia Dose Expansion Part ... 55
3.4 Phase Ib ... 55
3.5 End of Study ... 55
3.6 Data Safety Monitoring Committee (SMC) ... 55

4 Selection of Subjects ... 56

4.1 Inclusion Criteria ... 56

4.1.1 Inclusion Criteria for Molecular Screening 56
4.1.2 Clinical Screening Criteria for Inclusion 56
4.2 Exclusion Criteria 58
4.3 Criteria for Termination of Treatment..60
4.3.1 Treatment of Patients who Discontinue Treatment...61
4.3.2 Substitution of Patients who Discontinue Treatment......................................61
4.4 Determination of Recommended Phase II Dose 61
5 Study Drug and Study Method...62
5.1 Study Drug...62
5.2 Packaging and Labeling of Study Drug 62
5.3 Storage and Shipment of Study Drug 62
5.4 Drug Management 62
5.5 Drug Dispensing 62
5.6 Dose and Frequency of Administration 63
5.7 Method of Administration..63
5.8 Concomitant Medications 64
5.9 Permitted and Prohibited Drugs and Non-drug Therapies................................65
5.9.1 Permitted Drugs and Non-drug Therapies 65
5.9.2 Prohibited Drugs and Non-drug Therapies 65
5.10 Overdosage 66
5.11 Treatment Compliance...66
5.12 Principles for Treatment of Adverse Events During the Study.........................66
5.13 Monitoring and Management of Hyperglycemia in the Study 71
5.14 Female subjects and female partners of male patients must use highly effective contraception during the study and within 6 months after the last dose of study drug. 72
6 Study Procedures 72
6.1 Phase Ia 72
6.1.1 Molecular Screening Phase (Prior to the Clinical Screening Phase) 72
6.1.2 Clinical Screening/Baseline Phase (From Day -28 to Day -1) 73
6.1.3 Single Dose Phase (Day 1 to Day 7)..75
6.1.4 Continuous Dose Phase (First Cycle ± 2 Days; ± 3 Days of Cycle 2 and Each Subsequent Cycle)..76
6.1.5 Early Termination/End of Treatment (Within 7 Days after End of the Last Dose± 3 Days)...78
6.1.6 Safety Visit (Within 30 Days ± 7 Days after the Last Dose)..........................78
6.2 Phase Ib..79
6.2.1 Molecular Screening Phase (Prior to the Clinical Screening Phase) 79

6.2.2 Clinical Screening/Baseline Phase (From Day -28 to Day -1) 79
6.2.3 Study Treatment Phase (28-Day Cycle ± 3 Days) 81
6.2.4 Early Termination/End of Treatment (Within 7 Days after End of the Last Dose± 3 Days) 82
6.2.5 Safety Follow-up (Within 30 Days ± 7 Days after the Last Dose) 83
6.2.6 Survival Follow-up 83
7 Study Assessment 84
7.1 Safety Assessment 84
7.1.1 Safety Endpoints 84
7.1.2 Adverse Event Assessment 84
7.1.3 Laboratory Tests 89
7.1.4 Other Safety Evaluation 90
7.2 Efficacy Evaluation 90
7.3 Pharmacokinetic Assessment 91
7.3.1 Blood Sample Collection 91
7.3.2 Pharmacokinetic Endpoints 92
7.3.3 Blood Sample Handling and Transport 92
7.4 Assessment of Tumor Markers and Pharmacodynamic Markers 92
7.4.1 Rationale for Test Items 92
7.4.2 Requirements for Tissue Samples 93
7.4.3 Summary of Biomarker Sampling 94
8 Data Management 95
8.1 Data Entry 95
8.2 Database Lock 95
9 Statistics and Statistical Analysis 95
9.1 Sample Size 96
9.2 Analysis Sets 96
9.3 Safety Analysis 96
9.4 Efficacy Analysis 97
9.5 PK Analysis 97
9.6 Tumor Markers and PD Analysis 98
9.7 Interim Analysis 98
9.8 Final Analysis 98
10 Study Management 98
10.1 Ethical Considerations 98
10.2 Informed Consent 98
10.3 Compensation for Health Damage of Subjects 99

10.4 Recording and Retention of Study Data 99
10.5 Return or Destruction of Investigational Product(s)/Therapeutic Products 99
10.6 Quality Control and Quality Assurance 99
10.6.1 Monitoring and Auditing 100
10.6.2 Audits and Inspections 100
10.7 Amendments to Study Protocol 100
10.8 Protocol Violation 100
10.9 Study Termination 101
10.10 Study Report 101
10.11 Confidentiality and Publication of Study Results 101
11 References 102
12 Appendices 103
Appendix 1.1 List of Phase Ia Assessments (Single Dose Phase + Continuous Dose Phase) 103
Appendix 1.2 List of Phase Ia Assessments (Continuous Dose Phase) 108
Appendix 2 PK Blood Sample Collection Schedule for Phase Ia 114
Appendix 3 List of Assessments in Phase Ib 116
Appendix 4 Estimation of Blood Volume to Be Collected (Phase Ia/Ib) 121
Appendix 5 Eastern Cooperative Oncology Group (ECOG) Performance Status 125
Appendix 6 Response Evaluation Criteria in Solid Tumors (RECIST1.1) 126
Appendix 7 New York Heart Association (NYHA) Functional Classification 141
Appendix 8 Cockcroft and Gault formula 142
Appendix 9 Fridericia's formula 143
Appendix 10. List of Prohibited QT Interval-Prolonging Medications 144
Appendix 11 List of QT Interval-Prolonging Drugs to be Used with Caution 146

List of Tables

Table 1 Criteria for Dose-limiting Toxicity 21
Table 2 Main Physical and Chemical Parameters of CYH33 38
Table 3 Summary of In Vivo Anti-tumor Efficacy of CHY33 39
Table 4 Criteria for Discontinuation, Resumption, Dose Reduction, and Permanent Discontinuation of CYH33 67
Table 5 Summary of Biomarker Sampling 94

List of Figures

Figure1 Schematic Diagram of mTPI2 Design Escalation [19]: 50
Figure2 Schematic Diagram of Dose Escalation and Expansion Design 52

Protocol Approval Signature Page

**Protocol title:**	A multi-center, open-label, single-arm dose escalation and expansion Phase I clinical study to evaluate the safety, tolerability, pharmacokinetic characteristics and preliminary efficacy of CYH33 in patients with advanced solid tumors
**Protocol No.:**	CYH33-101
**Version number and date:**	September 24, 2019, Version 5.0

Sponsor: Shanghai Haihe Biopharma Co., Ltd.

Sponsor's address: No. 421 Newton Road, Zhangjiang Hi-Tech Park, Pudong New District, Shanghai

Approver Name:

Role:

Signature of approver: ______________ Date: _________________

Protocol Signature Page

**Protocol title:**	A multi-center, open-label, single-arm dose escalation and expansion Phase I clinical study to evaluate the safety, tolerability, pharmacokinetic characteristics and preliminary efficacy of CYH33 in patients with advanced solid tumors
**Protocol No.:**	CYH33-101
**Version number and date:**	September 24, 2019, Version 5.0

I have read this protocol and agree to conduct this clinical study in accordance with this protocol, current laws and regulations, and the ethical principles set forth in Declaration of Helsinki.

Name of coordinating investigator:

Signature of coordinating investigator: _____________ Date:________________

Protocol Signature Page

**Protocol title:**	A multi-center, open-label, single-arm dose escalation and expansion Phase I clinical study to evaluate the safety, tolerability, pharmacokinetic characteristics and preliminary efficacy of CYH33 in patients with advanced solid tumors
**Protocol No.:**	CYH33-101
**Version number and date:**	September 24, 2019, Version 5.0

I have read this protocol and agree to conduct this clinical study in accordance with this protocol, current laws and regulations, and the ethical principles set forth in Declaration of Helsinki.

Investigator name:

Signature of investigator: ________________ Date:________________

Protocol Synopsis

**Protocol No.:**	CYH33-101	
**Protocol Title:**	A multi-center, open-label, single-arm dose escalation and expansion Phase I clinical study to evaluate the safety, tolerability, pharmacokinetic characteristics and preliminary efficacy of CYH33 in patients with advanced solid tumors	
**Study Drug:**	CYH33	
**Phase of Study:**	Phase I	
**Number of Patients:**	It is expected that approximately 60 patients will be enrolled in Phase Ia [redacted]. The total number of patients will depend on the needs of dose escalation and dose expansion assessments.	
**Number of Study Sites:**	About 5 sites in Phase Ia [redacted]. The number of sites can be adjusted based on actual enrollment.	
**Phase of Study:**	Phase Ia: screening phase (Day -28 to Day -1); single dose phase (7 days), and continuous dose phase (with 28 days as one cycle). The necessity of a single dose phase or the length of the single dose phase for subsequent subjects will be decided based on the PK data observed in the first three dose groups. In these phases, the dose-limiting toxicity (DLT) observation phase is Cycle 1 of the continuous dose phase (28 days), and for subjects receiving a single administration, it lasts 35 days, i.e., the single dose phase plus Cycle 1 of the continuous dose phase; safety follow-up phase (30 days after the last dose)<br>[redacted]<br>All patients will receive treatment until disease progression, unacceptable toxicity, death, or discontinuation study by investigator's decision, or consent withdrawal by patient.<br>Initiation of study: it is estimated in February 2018<br>End of study: it is estimated in August 2021	
**Study Objectives and Endpoints**	**Study objectives**	**Study endpoints**
Primary	• Phase Ia: to determine the safety, tolerability and maximum tolerated dose (MTD) of oral CTH33 monotherapy in patients with advanced solid tumors who have failed or cannot tolerate standard treatment or currently have no standard treatment, and determine the	• Phase Ia: Type and frequency of treatment-emergent adverse events (TEAE), and evaluation of toxicity grades according to NCI CTCAE version 4.03; Laboratory test results, electrocardiogram (ECG) and cardiac imaging findings and physical examination findings (including vital signs, weight and ECOG

	recommended Phase 2 dose (RP2D).	performance status score), etc.<br>• Phase Ia: number and proportion of patients experiencing DLT in the DLT observation phase (approximately 28 days after the first dose, and 35 days after the first dose for patients receiving a single administration) (Phase Ia dose escalation part and Phase Ia dose expansion part).<br>• Maximum tolerated dose (MTD). If MTD is not observed, the RP2D will be determined through PK (pharmacokinetic)/PD (pharmacodynamic) data, safety and preliminary efficacy.
	[redacted]	[redacted]
Secondary	• Phase Ia: to assess the preliminary efficacy of oral CTH33 monotherapy in patients with advanced solid tumors who have failed or cannot tolerate standard treatment or currently have no standard treatment	• Phase Ia: response assessment endpoints: objective response rate (ORR), progression-free survival (PFS), duration of response (DoR) and disease control rate (DCR), clinical benefit rate (CBR).

	• Phase Ia: to determine the pharmacokinetic (PK) characteristics of CYH33 and its metabolite (I27) after single and continuous oral administration of CYH33.<br><br>[redacted]	• Phase Ia: PK parameters of CYH33 and its metabolite (I27), including $AUC_{0\text{-}last}$, $AUC_{0\text{-}24h}$, $AUC_{0\text{-}\infty}$, C_{max}, t_{max}, $t_{1/2}$, Vz/F, CL/F, ARC_{max}, $ARAUC_{0\text{-}24h}$, LI.<br><br>• [redacted]
	[redacted]<br><br>[redacted]	[redacted]<br><br>[redacted]<br><br>[redacted]

Exploratory	• To explore the relationship between the efficacy of CYH33 treatment and potential tumor biomarkers.	• The treatment response of CYH33 per tumor biomarker status (including but not limited to *PIK3CA*-alteration, *KRAS*, *BRAF* mutation, *AKT* alteration, etc.).
**Study design:**	This is a multi-center, open-label, single-arm Phase I clinical study of dose escalation and dose expansion of oral CYH33 monotherapy in patients with advanced solid tumors who have failed or cannot tolerate standard treatment or currently have no standard treatment. Patients in the Phase Ia expansion part [redacted] must agree in writing to provide archived tumor tissue sections or/and wax blocks or/and fresh tumor tissue to be delivered to the central laboratory for confirmation of *PIK3CA* gene alteration status before the screening phase.<br>The study consists of Phase Ia and Phase Ib. The Phase Ia dose escalation part will observe the safety, tolerability and pharmacokinetic characteristics of CYH33 in patients with advanced solid tumors who have failed or cannot tolerate standard treatment or currently have no standard treatment, and determine the MTD and RP2D;Before the determination of the MTD or RP2D, a safe and considerably effective dose level will be selected for synchronous expansion. Patients with advanced solid tumors who have failed or cannot tolerate standard treatment or currently have no standard treatment with *PIK3CA*gene mutation will be included to further assess the safety, tolerability and preliminary efficacy. [redacted]<br>**Phase Ia dose escalation part:**<br>This part includes two dosing phases: single dose phase and continuous dose phase .<br>**1. Single dose phase (Day 1 to Day 7):**<br>Patients in the first 3 dose groups are orally administered one dose of CYH33 in the fasting state (fasting for at least 2 h pre-dose and post-dose) and are observed for 7 days. If DLT does not occur in the observation phase, the patient enters into the continuous dose phase.<br>The necessity of a single dose phase or the length of the single dose phase for subsequent subjects will be decided by the SMC based on the PK data observed in the first 3 dose groups.<br>**2. Continuous dose phase (from Cycle 1):**	

	Patients in the first 3 dose groups are orally administered CYH33 in the fasting state (fasting for at least 2 h before and after administration) daily for 28 consecutive days as one cycle.<br><br>**Principle for dose escalation:**<br>• According to the existing preclinical data, the initial dose is determined as 1 mg daily, and 1 evaluable male patient will be enrolled. According to the study data in the 1 mg dose group, the Data Safety Monitoring Committee (SMC) will discuss and decide whether 1 patient each can be enrolled in the subsequent dose groups and follow this principle: 1 patient will be enrolled in each dose group for dose escalation. Dose escalation in the same patient is not acceptable. Whena study drug-related DLT is observed for the first time or any study drug-related CTCAE grade 2 toxicity (regardless of the type) is observed in ≥ 2 subjects in the DLT observation phase, this dose level will be converted to guide toxicity monitoring and dose escalation by using the modified Toxicity Probability Interval method 2 (mTPI2) . At least 3 assessable subjects should be enrolled at each dose level.<br>• Since a gender difference in drug metabolism has been observed in preclinical studies, only male patients will be enrolled in the ATD phase. Later according to the PK data in male subjects, the SMC will decide the dose for enrolled female subjects, and enroll 1 female subject to determine whether there is any gender difference in PK parameters.<br>• Principle for dose escalation using mTPI2:<br>1. When a study drug-related DLT is observed for the first time or any study drug-related CTCAE grade 2 toxicity is observed in ≥ 2 subjects in the DLT observation phase, this dose group will enter mTPI2 escalation, and three subjects will be enrolled at each dose level. If none of the 3 patients experiences DLT, the next schedule dose group will be opened to recruit 3 patients;<br>2. If 1 of the 3 patients experiences DLT, 2 more patients will be added to this dose group (see Figure 1: Dose Design Escalation Diagram using mTPI2). Five patients will allow the decision-making on whether to step up or down a dose level;<br>3. If 2 of the 3 patients experience DLT on a dose level, additional participants will be entered at the next lower dose group;<br>4. The number of patients to be enrolled should be calculated based on Figure 1 from the number of existing patients at this dose and the number of DLTs. Each time no more than 3 patients will be enrolled. The SMC will decide when to stop enrollment for Phase Ia escalation.

	• After the observation of DLT in each dose group, the SMC will evaluate the safety events, efficacy and PK/PD, and decide the next dose level for observation or dosing frequency. If necessary, it is allowed to increase a new dose level between two explored dose levels for exploration. The MTD will eventually be determined.<br>• Definition of MTD: the maximum dose at which the incidence of DLT is lower than 33% in Phase Ia.<br>• If the MTD is not observed, the RP2D will be determined in light of the safety, efficacy and PK/PD data in Phase Ia. If necessary, the RP2D can be adjusted in the course of the Phase Ib study.<br>**Phase Ia dose expansion part:**<br>In the course of Phase Ia dose escalation, a safe and considerably effective dose is selected for the Phase Ia expansion study, while the Phase Ia dose escalation study can continue synchronously. In the Phase Ia dose expansion part, DLT events will also be observed synchronously in the DLT observation phase. At any dose level or if necessary, combined with the effective dose predicted with the PK/PD model, the SMC can decide to enter the expansion study at this dose level to further investigate the safety, tolerability and efficacy of this dose level. The subjects enrolled in the Phase Ia expansion part are patients with advanced solid tumors with *PIK3CA*gene mutations, and the total number of subjects for expansion will be decided by the SMC based on the proportions of patients with CR, PR and SD, as well as on the safety and tolerability. The SMC will choose dose levels and some subjects for a PK study in the Phase Ia dose expansion part according to the needs of PK studies.<br>[redacted]<br>[redacted]<br>**Dose modification:**<br>Among patients in the Phase Ia dose escalation part, if any DLT event stipulated in the protocol occurs within the DLT observation phase (within 28 days after the first dose, and within 35 days after the first dose for patients receiving a single administration, i.e. the first treatment cycle of both the single dose phase and the

continuous dose phase), the drug should be permanently discontinued.

During the treatment phase after the DLT observation phase in the Phase Ia dose escalation part, during the treatment [redacted] in the Phase Ia dose expansion part, and throughout Phase Ib, all patients are allowed to have two dose reductions at most (the minimum dose is the initial dose). After a dose reduction, it is not allowed to restore to the previous higher dose. If the administration is discontinued due to a study drug-related AE for more than 21 days, the patient should permanently withdraw from the study, unless there is enough evidence of treatment benefit. If the patient previously benefited from the treatment with CYH33, the investigator can discuss with the sponsor about whether the administration should be resumed 21 days after discontinuation.

Dose up-regulation for individual patients:

Dose up-regulation is not allowed for the same patient within the first 4 treatment cycles. After 4 treatment cycles, if the patient has not experienced any study-drug related CTCAE ≥ grade 2 toxicity, and the investigator considers that a higher dose may benefit the patient, the dose can be up-regulated to a higher dose, and the new dose level after up-regulation must have gone through safety evaluations and not exceed the maximum tolerated dose (MTD).

DLT assessment:

DLT is defined as an adverse event or laboratory abnormality that occurs within 28 days after the first dose in Phase Ia (within 35 days after the first dose for patients receiving a single administration, i.e. 7 days of the single dose phase + 28 days of Cycle 1 of the continuous dose phase) and meets all the following criteria:

- It is judged by the investigator and/or sponsor to be unrelated to the disease itself, disease progression, concomitant diseases or concomitant medication, and thus considered to be related to CYH33;
- And meets any of the criteria in the table below (unless otherwise specified, the NCI CTCAE version 4.03 criteria will be used for grading).

Table 1 Criteria for Dose-limiting Toxicity

**Toxicity**	**Any of the following events will be regarded as DLT**
Hematology	Grade 4 neutropenia (neutrophil count < 0.5×10^9/L);<br>Grade 3 thrombocytopenia (platelet count <50×10^9/L) with a significant bleeding tendency, persisting for >7 days, and failing to return to ≤ grade 2 or baseline level within 7 days after the study drug is suspended;

	Grade 3 neutropenia with fever (neutrophil count $<1.0\times10^9$/L, fever ≥ 38.5°C), persisting for >7 days;<br>Other grade 4 hematological toxicities.
Hyperglycaemia	Grade 2 hyperglycemia (fasting blood glucose 8.9 - 13.9 mmol/L or 160 mg/dL-250 mg/dL), and after appropriate anti-diabetic treatment (e.g., metformin, hypoglycemic sulfonylureas, etc.) while maintaining the current dose level of CYH33 treatment), the blood glucose fails to return to normal fasting blood glucose or baseline levels within ≤ 14 days;<br>or Grade 3 hyperglycemia (fasting blood glucose 13.9-27.8 mmol/L or 250 -500 mg/dL) or asymptomatic Grade 4 hyperglycemia (fasting blood glucose > 27.8 mmol/L or 500 mg/dL), which does not return to ≤ Grade 2 hyperglycemia within ≤ 7 days after suspension of CYH33 and adequate/appropriate anti-diabetic treatment;<br>or symptomatic (symptoms can include, but are not limited to, thirst, polydipsia, polyuria, fatigue, blurred vision, and even loss of consciousness) Grade 4 hyperglycemia (fasting blood glucose > 27.8 mmol/L or 500 mg/dL);<br>In all cases, fasting blood glucose should be retested within 24 h for confirmation.
Non-hematological toxicity	≥ Grade 3 non-hematological (except hyperglycemia) toxicities, except for the following conditions:<br>Nausea, vomiting, diarrhea, constipation and electrolyte imbalance which return to ≤ Grade 2 or baseline levels within 3 days after adequate/appropriate supportive care;<br>Fatigue persisting for ≤ 7 days after adequate/appropriate supportive care;<br>Nonclinically significant laboratory changes, which will not lead to discontinuation of the study drug per the investigator's judgment;<br>Grade 3 blood pressure increased, which can be spontaneously relieved or be controlled by medications to below 140/90mmHg or baseline levels within 3 days;
	Other clinically significant and unacceptable toxicity of any grade which requires termination of the study drug per the investigator's judgment, and will be determined by the SMC as DLT.

Tumor assessments:

The anti-tumor activity of CYH33 will be assessed in the study population per RECIST version 1.1. Both Phase Ia and Phase Ib will use 28 days as 1 cycle. Patients will receive tumor assessments every 6 weeks (42 ±7 days). All assessments meeting

	the criteria for partial response (PR) or complete response (CR) should be re-assessed 6 weeks (42 ±7 days) later for response confirmation before PR or CR can be determined until disease progression, unacceptable toxicity, death, or discontinuation study by investigator's decision, or consent withdrawal by patient.<br>**Determination of the recommended Phase 2 dose (RP2D):**<br>The SMC will determine the RP2D in light of the Phase Ia safety, efficacy and PK/PD data. If necessary, the RP2D can be adjusted in the course of the Phase Ib study.<br>**Data Safety Monitoring Committee (SMC):**<br>During the study, an SMC composed of site principal investigators enrolling subjects in the Phase Ia escalation part, independent clinical oncologists (if necessary), the sponsor's medical monitors and drug safety physicians will be established to review the safety, PK/PD and efficacy data arising from the study, decide the assignment of patients, dose levels and dosing frequency in each dose group in the Phase Ia dose escalation phase, and recommend and choose doses for entering the Phase Ia expansion part. The SMC will decide the MTD/RP2D. After deciding the MTD, the SMC may evaluate whether other modes of administration are required (e.g., intermittent dosing). PK experts and biostatisticians, if necessary, may attend the meeting to provide advice, but have no right to vote.<br>**End of study:**<br>The last enrolled patient completes two tumor assessments after the first dose, has disease progression or unacceptable toxicity, withdraws from the study or dies, whichever occurs first.<br>By then, if some patients are still continuing the treatment, the drug will continue to be supplied until disease progression, unacceptable toxicity, patient withdrawal at the discretion of the investigator, or withdrawal of informed consent by the patient, or death.
**Study population:**	Phase Ia dose escalation part: patients with advanced solid tumors who have failed or cannot tolerate standard treatment or currently have no standard treatment.<br>Phase Ia dose expansion part: patients with advanced solid tumors who have failed or cannot tolerate standard treatment or currently have no standard treatment with *PIK3CA*gene mutations.<br>[redacted]
**Inclusion criteria:**	Molecular screening criteria for inclusion, applicable to the Phase Ia dose expansion part [redacted]:

	Patients must meet all the following criteria:<br>**1.** Patients who sign the informed consent form for molecular screening before any study-specific operations.<br>**2.** Patients who provide diagnostic evidence for *PIK3CA* gene mutations.<br>Evidence for *PIK3CA* gene mutations can be obtained through the following channels:<br>a. Pathological and genetic diagnosis reports of patient tumor tissue provided by existing medical/diagnostic institutions. The patients can enter the clinical screening and subsequent study phases, and it is required to forward tumor tissue specimens (at least 10 unstained tumor tissue sections; or/and formalin-fixed paraffin-embedded pathological tumor tissue or/and fresh tumor tissue) and copies of the corresponding pathology reports and genetic diagnosis reports to the designated screening laboratory for confirmation.<br>b. If no medical/diagnostic institution’s report proves that the patient's tumor tissue has a *PIK3CA* gene mutation, it is required to forward tumor tissue specimens (at least 10 unstained tumor tissue sections; or/and formalin-fixed paraffin-embedded pathological tumor tissue or/and fresh tumor tissue) and copies of the corresponding pathology reports and genetic diagnosis reports must be sent to the designated screening laboratory for molecular screening. Each site can conduct the clinical screening of patients only when the *PIK3CA* genetic test result is available.<br><br>Clinical screening criteria for inclusion:<br>Patients must meet all the following criteria prior to enrollment:<br>1. Patients who sign the informed consent form (ICF) for clinical screening. The ICF must be signed before any study-specific operations.<br>2. Male or female patients ≥ 18 years old.<br>3. Phase Ia: patients with histopathologically or cytologically confirmed locally advanced or metastatic solid tumors who have failed or cannot tolerate standard treatment regimens or currently have no standard treatment regimen (including patients who reject any chemotherapy). [redacted]

	[redacted]<br>4. In the screening phase, if agreed, patients can provide tumor tissue sections (unstained, approximately 4-30 sections); or/and formalin-fixed paraffin-embedded histopathological tumor samples (200 mm^3) and/or fresh tumor samples (20-150 mg/1-2 pieces of puncture biopsy tissue) and send them to the central laboratory for determination of responding tumor biomarkers.<br>Note: Tumor tissue shall be obtained from radiotherapy-naïve sites.<br>5. An ECOG performance status score of 0 or 1.<br>6. Life expectancy ≥ 12 weeks.<br>7. Patients who can swallow tablets when enrolled in the study.<br>8. Patients with at least one measurable lesion assessed according to RECIST version 1.1.<br>Note: A lesion previously treated by radiotherapy cannot be regarded as a target lesion, unless progression is documented after radiotherapy.<br>9. Patients' laboratory tests meet the following requirements to confirm adequate organ and hematopoietic system functions:<br>Absolute neutrophil count (NEU) ≥ 1.5 × 10^9/L;<br>Hemoglobin (HGB) ≥ 90 g/L (in the case of no blood transfusion in the past two weeks);<br>Platelet (PLT) count < 90 × 10^9/L;<br>Serum total bilirubin (TBIL) ≤ 1.5×ULN (if Gilbert syndrome is diagnosed, serum total bilirubin ≤ 3 × ULN is allowed but serum direct bilirubin (DBIL) must be ≤ 1×ULN);<br>Aspartate aminotransferase (AST) and alanine aminotransferase (ALT) ≤ 2.5×ULN (if there is liver metastasis, AST and ALT ≤ 5 × ULN are allowed);<br>Serum creatinine (Scr) ≤ 1.5 × ULN; if there is an uncertain threshold, the creatinine clearance (Ccr) calculated should be ≥ 50 mL/min (calculated using the Cockcroft and Gault formula, see Appendix 8);<br>International normalized ratio (INR) ≤ 1.5 or activated partial thromboplastin time (APTT) ≤ 1.5 × ULN;<br>Fasting blood glucose (GLU) ≤ 126 mg/dL or ≤ 7.0 mmol/L;<br>Glycated hemoglobin (HbA1c) ≤ 7.5%;<br>Serum amylase (AMS) ≤ 1 × ULN;<br>Serum lipase (LPS) ≤ 1 × ULN.

	10. Patients who are able to follow the study procedures, restrictions and requirements at the investigator's discretion.<br>11. Both female and male patients of childbearing age must agree to use effective contraceptive measures from the date of signing the informed consent form to within 6 months after the last dose.
**Exclusion criteria:**	Patients who meet any of the following criteria may not enter the study:<br>1. Patient's previous history of anti-tumor treatment complies with one of the following conditions:<br>a. Patients who have received the last dose of any anti-cancer treatment (including chemotherapy, targeted therapy, biological therapy, or hormonal therapy, etc.) prior to the study drug administration less than 28 days or less than 5 half-lives (whichever is shorter) from the first dose of this study; Patients who have received traditional Chinese medicine for anti-tumor treatment (the indications on the package insert of the traditional Chinese medicine shall prevail) without a 14-day washout phase;<br>b. Patients who have undergone major surgery within 28 days prior to the study drug administration or have not yet fully recovered from the previous surgery (in China, the definition of major surgery is based on grade 3 or 4 surgery defined in Management Measures for Clinical Application of Medical Technology implemented on May 1, 2009);<br>c. Patients who have received radiotherapy (including whole brain radiotherapy) within 28 days prior to the study drug administration;<br>d. Patients who have previously received and failed treatment with PI3K inhibitors, AKT inhibitors or mTOR inhibitors.<br>2. Patients have not yet recovered from the toxic reactions (except hair loss and pigmentation) caused by previous anti-tumor treatments (CTCAE > grade 1); Patients who have previously received platinum-based therapy, whose neurotoxicity needs to be restored to CTCAE ≤ grade 2; Patients who have not yet recovered to CTCAE grade 0-1 from the previous radiotherapy (except for permanent radiotherapy damage).<br>3. Patients with central nervous system metastases or malignant tumor-related epilepsy requiring clinical intervention.<br>a. Patients with previously treated stable central nervous system metastases and asymptomatic central nervous system metastases can participate in the study. Patients

	must have completed topical treatment (including radiotherapy and/or surgery) for central nervous system metastasis ≥ 14 days prior to initiation of the study drug. Meanwhile, if the patient is receiving steroid treatment, the following criteria must be met:<br>1. The patient must be receiving a steady dose or is reducing the dose (≤ dexamethasone 4 mg/day or other steroids with the same anti-inflammatory effects).<br>2. The dose of steroids has not been increased for 14 days before the start of the study treatment.<br>4. Patients diagnosed with pancreatic cancer; Patients with concurrent malignancies or other malignancies within 5 years prior to the study recruitment (excluding properly treated basal or squamous cell carcinoma, non-melanoma skin cancer, or radically resected cervical carcinoma in situ).<br>5. Patients who have been treated with a granulocyte colony-stimulating factor within 14 days prior to the study drug administration.<br>6. Patients who have received a blood transfusion or platelet transfusion therapy within 14 days prior to the study drug administration.<br>Note: Erythropoietin or erythropoietin treatment can be maintained if it has been used prior to recruitment.<br>7. Patients who are currently receiving medications known to have a risk of prolonging QT interval or inducing torsades ventricular tachycardia, and can neither stop the treatment nor switch to another drug prior to the start of the study drug administration.<br>8. Patients who are currently treated with warfarin or any other coumarin derivative.<br>9. Patients with known immediate or delayed hypersensitivity reaction(s) to fellow drugs and excipients.<br>10. Patients who concurrently participate or have participated in another therapeutic clinical trial within 4 weeks prior to the first dose of the study drug. If the patient has participated in a non-interventional clinical trial (e.g., epidemiological studies) or is in the survival follow-up phase of an interventional clinical trial, the patient can be enrolled in this study.<br>11. Patients with hepatitis B virus (HBV) or hepatitis C virus (HCV) infection. All patients must receive HBV and HCV screening in the screening phase. HBsAG-positive patients can be enrolled in this study if HBV DNA is <1,000 copies/ml or 200 IU/ml, but during the study phase, HBV DNA should be monitored in each treatment cycle, and the patient should receive antiviral treatment if necessary.

HCV antibody-positive patients, if negative for HCV RNA tests, can be enrolled in this study.

12. Patients with human immunodeficiency virus (HIV) infection. All patients must receive HIV screening in the screening phase, and HIV-positive patients shall not be enrolled.
13. Patients' cardiac function and diseases meet one of the following conditions:
    a. During the screening phase, three electrocardiogram (ECG) measurements are performed at the study site, and the mean of the three measurements is calculated according to the instrument's QTc formula: male > 450 ms, and female QTc > 470 ms (corrected with the Fridericia's formula);
    b. Clinically significant arrhythmia, including but not limited to complete left bundle branch conduction abnormality, second-degree atrioventricular block, and PR interval > 250 ms;
    c. Any risk factors that increase QTc prolongation, such as heart failure, hypokalemia, congenital QT prolongation syndrome, familial long QT syndrome or a family history of sudden death under 40 years of age, and administration of any medications prolonging the QT interval, etc.;
    d. ≥ Grade 3 congestive heart failure according to New York Heart Association (NYHA) classification;
    e. Unstable angina or new angina (within 3 months). Myocardial infarction occurring within 6 months prior to the study drug administration;
    f. Left ventricular ejection fraction (LVEF) < 50% based on echocardiography (ECHO) or multiple-gated acquisition scan (MUGA);
    g. Poorly controlled hypertension (defined as systolic blood pressure ≥ 140 mmHg and/or diastolic blood pressure ≥ 90 mmHg after optimal antihypertensive drug treatment) despite optimal drug intervention at the investigator's discretion;
    h. Patients who have received or are about to receive placement of a pacemaker.
14. Arterial or venous thromboembolic events such as cerebrovascular accidents (including TIA), deep vein thrombosis or pulmonary embolism occurring within 6 months prior to the study drug administration.
15. Poorly healing wounds, ulcers or fractures.
16. Presence of serious clinically active infections (CTCAE > grade 2).
17. Presence of epilepsy that requires drug treatment.
18. Dysphagia, or ulcerative colitis, Crohn's disease; Or patients who have undergone small bowel resection (more than one-third of the small intestine has been resected); Or

	uncontrollable nausea, vomiting, diarrhea, malabsorption syndrome and other diseases which may significantly affect the administration or absorption of CYH33. Patients with a history of digestive tract perforation or obstruction may not be enrolled.<br>Note: Patients with less than one-third of the small intestine resected can be enrolled in this study if their drug absorption is not affected at the investigator's discretion.<br>19. Patients with severe liver injury, end-stage liver diseases of Child-Pugh grade B and C.<br>20. Patients with a medical history or disease state of great clinical significance, or any disease state that is unstable or may affect the assessment of the study results or the safety or compliance of the subject at the investigator's discretion.<br>21. Pregnant or lactating women.<br>22. Patients with a history of acute or chronic pancreatitis or risk factors that may increase the risk of pancreatitis.
**Study drug, and dosage and administration:**	• CYH33 scored tablets:<br>Strength: 1 mg, 10 mg and 50 mg.<br>• Dosage and administration:<br>Phase Ia: the starting dose is 1 mg. For subsequent doses, the magnitude of the next dose escalation will be regulated according to the PK data and the previous dose group's toxic reactions; Patients will be given a single oral dose in the fasting state on Day 1 of the single dose phase; In the continuous dose phase, patients will orally administer the drug in the fasting state once or several times daily for a 28 - day cycle.<br>[redacted]
**Safety evaluation:**	This mainly includes monitoring and recording AEs and SAEs, laboratory tests (including hematology, blood biochemistry and urinalysis) specified in the protocol, 12-lead electrocardiogram, vital sign measurements, etc. The severity of AEs will be evaluated per NCI CTCAE version 4.03 criteria.
**Efficacy evaluation:**	All patients will be assessed for tumor response by computed tomography (CT) or magnetic resonance imaging (MRI) using the Response Evaluation Criteria in Solid Tumors (RECIST) version 1.1, every 6 weeks (42 ± 7 days) (or according to actual clinical needs) until disease progression, unacceptable toxicity, death, or discontinuation study by investigator's decision, or consent withdrawal by patient.
**Pharmacokinetic evaluation:**	To detect concentration of CYH33 and its metabolite (I27) in plasma and investigate the metabolic characteristics of CYH33 in plasma, plasma samples will be collected at the following time points:

	**Phase Ia:**<br>**If there is a single dose phase:**<br>• Single dose phase:<br>o Day 1 (D1): within 15 min before administration, and 0.5, 1, 2, 4, 8 and 12 h post-dose;<br>o D2: 24 and 36 h post-dose;<br>o D3: 48 and 72 h post-dose;<br>• Cycle 1 (C1) of the continuous dose phase:<br>o Cycle 1 Day 1 (C1D1): pre-dose (within 15 min before administration);<br>o C1D8: pre-dose (within 15 min before administration);<br>o C1D15: pre-dose (within 15 min before administration);<br>o C1D28: pre-dose (within 15 min before administration), and 0.5, 1, 2, 3, 4, 6, 8, 12 and 24 h post-dose;<br>It will be decided according to the PK data in the first 3 dose groups whether subsequent subjects need a single dose phase or how long does it take, and the corresponding blood sampling sites should be decided.<br>**If there is no single dose phase, subjects will directly enter the continuous dose phase:**<br>PK blood collection points: C1D1: pre-dose (within 15 min before administration), and 0.5, 1, 2, 3, 4, 6, 8, 12 and 24 h post-dose; C1D8: pre-dose (within 15 min before administration); C1D15: pre-dose (within 15 min before administration); C1D28: pre-dose (within 15 min before administration), and 0.5, 1, 2, 3, 4, 6, 8, 12 and 24 h post-dose.<br>Among these points, the 24 h blood samples on C1D1 and C1D28 will be collected within 15 min before administration on C1D2 and C2D1.<br>The PK study will be carried out in the Phase Ia dose escalation part, and will be conducted at selected dose levels in some subjects in the PhaseIa dose expansion part.
**Pharmacodynamic evaluation**	[redacted]
**Tumor marker evaluation:**	[redacted]
**Statistical analysis**	

**Sample size:**	It is estimated that a total of about 60 evaluable patients will be enrolled in the Phase Ia study. [redacted] The actual number of cases will be decided by the SMC.
**Analysis Populations:**	DLT analysis set (only applicable to Phase Ia): patients who have experienced DLT within 28 days after the first dose (within 35 days after the first dose for patients receiving a single administration) or patients who have used the medication as planned on the first day and had Cycle 1 medication>=75% in the continuous dose phase.<br><br>Full analysis set: enrolled patients who have taken at least one dose of CYH33. The full analysis set will be applied to efficacy assessments.<br><br>Evaluable analysis set: patients who have taken at least one dose of CYH33 with baseline tumor assessment data and data of post-baseline tumor assessments at least once.<br><br>Safety analysis set: patients who have taken at least one dose of CYH33.<br><br>PK analysis set: including patients who have taken at least one dose of CYH33 and have at least one assessable PK data. Subjects with protocol violations seriously affecting the PK assessment results will be excluded out of the PK analysis set.<br><br>PD analysis set: including all patients who have received one dose of CYH33 and have evaluable PD data (without major protocol violation that may affect the PD data analysis).
**Safety analysis:**	Safety will be evaluated by summarizing DLTs, AEs, changes in laboratory test results and changes in vital signs. The number and proportion of patients who have experienced at least one DLT in the DLT observation phase of the Phase Ia study will be summarized by dose level group using descriptive statistics. Adverse events will be separately summarized on the basis of the single dose phase, Cycle 1 of the continuous dose phase and the entire treatment phase, and a statistical analysis will be performed on TEAEs, SAEs, treatment-related AEs and SAEs, grade 3 and above AEs and AEs leading to drug withdrawal.<br>Changes in laboratory test results will be summarized according to grading by the NCI-CTCAE version 4.03 criteria. For laboratory indicators, the maximum toxicity occurring in the study will be summarized using number and percentage. Changes in vital sign and ECOG scores will be compared with baseline levels and descriptive statistical analysis will be performed.
**Efficacy analysis:**	Confirmed ORR will be calculated, and 90% Clopper-Pearson confidence interval will also be calculated. Survival analysis will be performed for PFS and DoR by using the Kaplan-Meier curve according to the investigator's assessment results, and descriptive

	statistical analysis will be performed for DCR and CBR. [redacted]
**Pharmacokinetic analysis**	At least the following parameters will be analyzed and calculated by using non-compartmental model from the single dose phase to the continuous dose phase according to individual concentration-time data of CYH33 and its metabolite:<br>Phase Ia:<br>• Single dose phase:<br>$AUC_{0-\infty}$, AUC_{0-last}, $AUC_{(0-24h)}$, C_{max}, t_{max}, $t_{1/2}$, CL/F and V_z/F<br>• Continuous dose phase:<br>$AUC_{(0-24h)}$, C_{max}, t_{max}, ARC_{max}, $ARAUC_{(0-24h)}$ and LI<br>Descriptive analysis for plasma concentration and PK parameters of CYH33 and its metabolite will be performed.
**Tumor markers and pharmacodynamic analysis**	[redacted]
**Protocol date:**	September 24, 2019

Abbreviations

Abbreviations	Full text
AE	Adverse event
ALT	Alanine transaminase
AMS	Serum amylase
APTT	Activated partial thromboplastin time
$ARAUC_{(0\text{-}24h)}$	Accumulation ratio of area under concentration-time curve from time 0 to 24 h
ARC_{max}	Accumulation ratio of maximum plasma concentration
AST	Aspartate transaminase
ATD	Rapid titration dose
ATP	Adenosine triphosphate
$AUC_{(0\text{-}24h)}$	Area under concentration-time curve from time 0 to 24 h
$AUC_{0\text{-}\infty}$	Area under concentration-time curve from time 0 (before administration) to infinity (∞)
$AUC_{0\text{-}last}$	Area under concentration-time curve from time point 0 (before administration) to last time point with measurable concentration
$AUC_{0\text{-}t}$	Area under the concentration-time curve from time 0 (before administration) to the last time point with measurable concentration
C1D1	Cycle 1 Day 1
CBR	Clinical benefit rate
Ccr	Creatinine clearance
cf DNA	Circulating free DNA
CL/F	Apparent clearance
C_{max}	Maximum plasma concentration
CNS	Central nervous system
CR	Complete response
CT	Computed tomography
CXDX	Day X of Cycle X
DBIL	Direct bilirubin
DCR	Disease control rate
DLT	Dose limiting toxicity
DoR	Duration of response
ECG	Electrocardiogram
ECHO	Echocardiograms
ECOG	Eastern Cooperative Oncology Group
eCRF	Electronic Case Report Form
EGF	Epidermal growth factor

GCP	Good Clinical Practice
G-CSF	Granulocyte colony-stimulating factor
GLU	Blood glucose
GM-CSF	Granulocyte macrophage colony-stimulating factor
HbA1c	Glycosylated hemoglobin
HBV	Hepatitis B virus
HCV	Hepatitis C virus
HED	Human equivalent dose
HGB	Hemoglobin
HGF	Hepatocyte growth factor
HIV	Human immunodeficiency virus
HNSTD	Highest non-severely toxic dose
IC_{50}	Half maximal inhibitory concentration
ICF	Informed Consent Form
ICH-E9	Statistical Principles for Clinical Trials (Chinese Version)
IEC	Independent Ethics Committee
IGF	Insulin-like growth factor
INR	International normalized ratio
LI	Linearity index
LLN	Lower limit of normal
LPS	Serum lipase
LVEF	Left ventricular ejection fraction
MLD	Minimal lethal dose
MRI	Magnetic resonance imaging
MTD	Maximum tolerated dose
mTOR	Mammalian target of rapamycin
mTPI	Modified toxicity probability interval
MUGA	Multiple uptake gated acquisition scan
NCI CTCAE	National Cancer Institute-Common Terminology Criteria for Adverse Events
NEU	Absolute neutrophil count
NMPA	National Medical Products Administration
NOAEL	No-observed-adverse-effect level
NYHA	New York Heart Association
ORR	Objective response rate
OS	Overall survival
PAM	PI3K-Akt-mTOR signaling pathway
PD	Pharmacodynamics

PDK	Phosphatidylinositol-dependent protein kinase
PDX	Patient-derived tumor xenograft
PFS	Progression free survival
PI3K	Phosphatidylinositol 3-kinase
PIK3CA gene	Proto-oncogene located on chromosome 3 (q25 to 27), p110α, encodes p110α, the catalytic subunit of PI3K
PK	Pharmacokinetics
PLT	Platelets
PR	Partial response
QT interval	Interval measured from the start of the Q wave to the end of the T wave
RECIST	Response Evaluation Criteria in Solid Tumors
RNA-Seq	RNA sequencing
RP2D	Recommended phase II dose
RR Interval	Distance from the start of R wave to the start of the next R wave
RTK	Receptor tyrosine kinase
SAE	Serious adverse event (SAE)
Scr	Serum creatinine
SD	Stable disease
SMC	Data Safety Monitoring Committee
$t_{1/2}$	Elimination half life
TBIL	Total bilirubin
TEAE	Treatment-emergent adverse event
t_{max}	Time to maximum plasma concentration
ULN	Upper limit of normal
V_z/F	Apparent volume of distribution

1 Study Background

1.1 Medical Backgroung

Cancer has emerged as a prevalent and common disease which seriously threatens human health and life. According to the 2012 World Cancer Report released by the World Health Organization, there were 14 million new cases of cancer and 8.2 million deaths from cancer worldwide in 2012. Of these, nearly half of the new cancer cases were from Asia, most of which were from China. The 2015 China Cancer Statistics released in early 2016 based on the statistics of the years 2009 to 2011 predicted[1] that there would be approximately 4.292 million new cases of cancer and 2.814 million deaths from cancer in 2015 in China. Among these cases, lung cancer is the malignancy with the highest mortality, followed by gastric cancer, esophageal cancer and liver cancer.

PI3K/AKt/mTOR Signaling Pathway

The PI3K/AKt/mTOR signaling pathway is one of the numerous mechanisms that regulate the cell cycle and apoptosis, and dysregulation of a single component of the pathway can lead to tumorigenesis[2]. Receptor tyrosine kinase (RTK), when activated by the growth factors, regulates the activation of PI3K/AKt signaling pathway. These growth factors include insulin-like growth factors (IGF), epidermal growth factor (EGF) and hepatocyte growth factor (HGF), which activate RTK through phosphorylation of tyrosine residues. While PI3K, on the other hand, activates the catalytic subunit of PI3K after binding to phosphorylated tyrosine residues. The catalytic subunit of Class IA PI3K, the p110α, activates PI3K through its binding to p85, thus further phosphorylating the phosphatidylinositol 4,5-diphosphate (PIP2) to phosphatidylinositol 3, 4,5-triphosphate (PIP3). The PIP3, a critical second messenger and mediator, recruits AKt from the cytoplasm to the cell membrane through interaction with the PH domain of AKt. Membrane translocation of AKt and the phosphorylation of the Thr308 and Ser473 sites by phosphatidylinositol-dependent kinase 1 (PDK1) and phosphatidylinositol-dependent protein kinase 2 (PDK2), respectively, are essential for AKt activation. AKt, when fully activated, regulates biological processes such as cell proliferation and apoptosis through its regulation of upstream and downstream. Meanwhile, PIP3 phosphatase PTEN (phosphatase and tensin homologue deleted on chromo-some ten) becomes a regulator of this pathway by maintaining the balance of pathway activation. The activated AKt phosphorylates tuberous sclerosis complex (TSC2) and PRAS40 can no longer inhibit mTOR. mTORC1 complex-phosphorylated ribosomal S6 kinase and transcription initiation factor 4E (4EBP1) can regulate protein synthesis, thereby regulating protein translation and cell growth[3].

Expression of PI3K/Akt signaling pathway in tumors

Dysregulation of the PI3K signaling pathway has almost been implicated in all human cancers. *PIK3CA* gene amplification and acquired gene mutations are highly prevalent in cancers and contribute to the activation of signals through PI3K pathway. *PIK3CA* is in fact one of the most frequently mutated oncogenes in human tumors [4-7]. *PTEN* gene mutations, deletions, and reduced expression levels are also frequently observed in human tumors [8]. PI3K is overexpressed in ovarian and cervical cancers, and its mutations have been observed in breast, glioblastoma, and gastric cancers. Although mutations in the Akt protein itself are rare, Carpten et al. [9] described that the Akt1 enzyme also occurred in a small fraction of breast, ovarian, and colorectal cancer cells in human. Studies have shown that oncogene *PIK3CA* mutations (rare frequency of gene variation and survival analysis in

thymic epithelial tumors) are present in approximately 2% - 5% of human solid tumors, with a proportion of about 32%, 27%, 25%, 8% and 4% in colon cancer, glioblastoma, gastric cancer, breast cancer and lung cancer, respectively. In other gastrointestinal cancers, the mutation rate is 11% in esophageal squamous cell carcinoma, and 6% in esophageal adenocarcinoma [9, 10]. Therefore, it can be predicted that the PI3K/Akt signaling pathway may serve as a potential target for clinical anti-tumor treatment.

Research and development status of PI3K inhibitors

Although substantial studies have been conducted on PI3K targets and their relationship with tumors worldwide over the past 30 years, only one PI3K inhibitor has been successfully marketed (idelalisib, a selective PI3K inhibitor, was approved in 2014 for the treatment of chronic lymphocytic leukemia, small lymphocytic leukemia and non-Hodgkin's lymphoma). Others are in clinical studies or even earlier stages, including: the selective PI3K inhibitor BYL719; Pan-PI3K inhibitors BKM120 and GDC-0032; PI3K/mTOR dual inhibitors BEZ235, PF-05212384, PF-04691502, GDC-0941 and SAR245409, etc.

Currently the confirmatory Phase III clinical studies include BELLE-2, BELLE-3 and BELLE-4 studies for BKM120, SOLAR-1 study for BYL719, Lung-MAP and SANDPIPER studies for GDC-0032 with main target indications of breast cancer and lung cancer.

1.2 Introduction to study drug

CYH33 is a new small molecule compound selectively inhibiting PI3Kα (see Table 2) with intellectual property rights independently developed by Shanghai Institute of Materia Medica, Chinese Academy of Sciences. CYH33 will be clinically developed by Shanghai Haihe Biopharma Co., Ltd.

Drug code: CYH33

[redacted]

[redacted]

[redacted]

Table 2 Main Physical and Chemical Parameters of CYH33

Parameter	Description
[Description]	Off-white or light yellow solid powder
[Crystal form]	Crystal, crystallinity 89.6%
[Molecular weight]	598.60 g/mol (free)<br>730.73 g/mol (salt form)
[Purity test]	99.13%
[Water]	5.2%
[pKa]	4.86 ± 0.06 in H_2O
[Solubility]	1.21 mg/ml in H_2O<br>pH dependent High solubility at pH <3
［log P］	2.89

1.2.1 Results of Nonclinical Pharmacodynamic Study

CYH33 could significantly inhibit the activity of the wild-type and mutant PI3K kinase, with slight effects on more than 300 other kinases, including other members of the phosphatidylinositol kinase-related kinase (PIKK) family. CYH33 also showed the selective inhibitory effect of PI3K at cellular level. By inhibiting the PIK3 mediated signaling pathway, CYH33 can block DNA synthesis at an early stage (G1 phase) and suppress cell proliferation, thus exhibiting remarkable anti-tumor activity both in vitro and in vivo, especially for esophageal cancers with the most frequently activated PI3K signaling pathway. At the same dosage, CYH33 showed superior anti-tumor activity over its counterpart candidate BYL719 under clinical study.

Based on the good activity of CYH33 at molecular and cellular levels, a murine model of esophageal cancer was further selected to investigate the antitumor effect of this candidate drug in vivo. KYSE410 and KYSE510, subcutaneous xenograft models in nude mice derived from human esophageal cancer cell lines, were used to investigate the in vivo activity of this compound. The results showed that oral administration of the compound at 50, 25 and 12.5 mg/kg for 21 days significantly inhibited the growth of these two subcutaneous xenografts, and the T/C ratio in 12.5 mg/kg dose group was approximately 30% only (Table 3).

The in vivo pharmacodynamics of CYH33 was evaluated using a patient-derived tumor xenograft (PDX) model from tumor specimens of esophageal cancer patients. Three models, EC104, EC063 and EC074, were used for the evaluation, and the results showed that CYH33 could inhibit the growth of esophageal carcinoma subcutaneous xenograft in these models in a dose-dependent manner. It was more sensitive in EC104 and EC063 models, with T/C ratio of only about 30% after oral administration of CYH33 at 12.5 mg/kg for 21

days, while T/C ratio reached 36.78% in EC074 after oral administration of CYH33 at 25 mg/kg for 21 days. The good tumor growth inhibitory effect of CYH33 on PDX model further suggests that this compound may have bright prospects for clinical application.

In all tested animal models of esophageal cancer disease, CYH33 demonstrated a better tumor-inhibiting efficacy than the positive control compound BYL719 or comparable to BYL719. In particular, in KYSE410 subcutaneous xenograft model in nude mice, the ability of BYL719 at 50 mg/kg to inhibit tumor growth was only comparable to that of CYH33 at 12.5 mg/kg (Table 3).

Table 3 Summary of In Vivo Anti-tumor Efficacy of CHY33

Group	Dose mg/kg	T/C%								
		Cell line-derived xenograft model				Patient-derived xenograft model				
		KYSE410		KYSE510		EC104		EC063		EC074
BYL-719	50	25.74	26.89	32.43	26.32	19.38	17.56	19.44	25.30	30.52
	12.5	29.09	NA	47.62	NA	NA	NA	NA	NA	NA
CYH33	50	3.37	6.56	23.54	11.61	24.71	13.78	16.56	10.18	24.27
	25	7.95	13.44	21.06	17.96	25.19	19.74	24.53	21.29	36.78
	12.5	32.05	31.97	52.39	30.96	21.32	40.54	24.24	27.69	55.94
	6.25	68.17	NA	41.50	39.47	54.84	54.20	30.69	39.25	80.46

T/C% is tumor/control ratio; "NA" stands for not assessed

A subcutaneous xenograft model of human esophageal cancer KYSE410 in nude mice that was sensitive to PI3K inhibitors was used, and the doses, 12.5 mg/kg (inhibition rate >67%), 25 mg/kg (inhibition rate >90%) and 50 mg/kg (inhibition rate >95%), that could significantly inhibit tumor growth in a dose-dependent manner after multiple doses in vivo assays were selected to further investigate the inhibition of CYH33 on PI3K signaling pathway in tumor tissues.

The results showed that CYH33 at three doses could significantly down-regulate the phosphorylation of Akt and rpS6 at 0.25 h (25 mg/kg, 50 mg/kg) or 0.5 h (12.5 mg/kg) after single dose. After CYH33 at each concentration reached the peak at 2 h, the concentration in tumor tissues then gradually decreased. Therefore, phosphorylated Akt and rpS6 recovered after 8 h. With time extended to 24 h after administration, phosphorylated Akt and rpS6 returned to the level equivalent to the blank control. The above results reflect that the inhibitory activity of CYH33 against PI3K pathway has certain dose dependency and time dependency, and this activity also has certain correspondence with the anti-tumor effect of CYH33 in the KYSE410 model, indicating that CYH33 significantly inhibited the growth of PI3Kα inhibitor-sensitive subcutaneous xenografts by targeting PI3Kα.

For detailed results of nonclinical pharmacodynamic study of CYH33, see Investigator's Brochure (IB)[12].

1.2.2 Nonclinical Pharmacokinetics

CYH33 displayed a marked gender difference in its pharmacokinetics in rats, with a plasma clearance CL of 34.0 mL/min/kg in females and 4.83 mL/min/kg in males, respectively; CYH33 had a low clearance in dogs with a plasma clearance CL of 6.75 mL/min/kg. CYH33 was widely distributed in rats and dogs, with a Vss, steady-state volume of distribution, of 1.96 L/kg and 1.86 L/kg, respectively. After intragastric administration, CYH33 exposure AUC_{0-t} in female rats was more than 12 times that in male rats. The absolute bioavailability of CYH33 was 19.0% in male rats (1 mg/kg), 53.7% in female rats (1 mg/kg), and 70.9% in dogs (0.05 mg/kg), respectively; Elimination of CYH33 was slower in female rats ($t_{1/2}$ 6.7 hr) than in male rats ($t_{1/2}$ 1.3-4.5 hr), with an elimination half-life of 5.1 hr in dogs. After intragastric administration, plasma exposure of amide hydrolysis metabolite I27 in rats was approximately 2% (males) and 16% (females) of that of parent drug, and exposure in dogs was approximately 1.4- to 1.8-fold higher than that of parent drug. Within the dose range of 1 to 12 mg/kg (male rats) and 1 to 6 mg/kg (female rats), the increases in AUC_{0-t} of CYH33 and I27 in rats were close to or less than dose proportional increase; The increases in AUC_{0-t} of both CYH33 and I27 in dogs were basically proportional to the dose increase over the dose range of 0.05 to 0.2 mg/kg. After continuous administration for 7 days, the accumulations of CYH33 and I27 were not significant in rats and dogs.

After intragastric administration of CYH33 in rats, the drug was mainly distributed in such organ tissues as liver, adrenal gland, digestive tract, pancreas, kidney, spleen and ovaries. The exposure in tissues was more than 4 times that in plasma, the exposure in brain tissue was approximately 20% of that in plasma, and the drug was basically not distributed into the red blood cells. The concentration of the parent drug at 72 h after administration was 0.5% lower than the peak concentration. The CYH33 exposure in tissues was approximately 7 - 18-fold higher in female rats than in male rats. The distribution profile of metabolite I27 was similar to that of parent drug. The plasma protein binding (PPB) of CYH33 in rats, dogs and human was 97.5%, 97.1% and 94.9%, respectively, without concentration dependency. The PPB of metabolite I27 in rats, dogs and human was 83.3%, 80.5% and 79.1%, respectively, without concentration dependency.

After intragastric administration of CYH33 in rats, a total of 14 metabolites were detected in vivo, with their major metabolic routes consisting of mono-oxidative hydrogenation (M5), dual oxidation (M6) and morpholine ring-opening (M1-2). It was mainly excreted in feces as parent drug and metabolites via bile. The cumulative excretion of parent drug in feces within 96 h after administration accounted for approximately 24% of the dose administered; Urinary excretion of parent drug and the metabolite I27 was less than 1% of the dose administered. After intragastric administration of CYH33 in beagle dogs, the main components were found to be the parent drug and amide hydrolytic metabolite I27 in plasma.

CYH33, up to 100 μM, did not inhibit CYP1A2, CYP2C8, CYP2B6, CYP2C9, CYP2C19, CYP2D6 and CYP3A4.

CYH33 was primarily metabolized by the amide bond hydrolysis in the hepatocytes of human, monkey, dog, rat, and mouse (I27, M3). The degree of hydrolysis of amide bonds

in hepatocytes was significantly higher in dogs than in other species. The extent of hydrolytic metabolism of amide bonds in monkey hepatocytes was low, but the extent of oxidative metabolism (M5, M6) was higher than that in other animal species. Oxidative metabolites of CYH33 were not detected in human hepatocytes, so the metabolic effect of oxidative metabolic enzymes in human liver on CYH33 was not evaluated. The liver and intestine homogenates of dogs were collected, confirming that the amide hydrolysis of CYH33 was mainly catalyzed by carboxylesterase in liver.

CYH33 was moderately permeable, and the efflux transporters may be involved in the transport across Caco-2 cells in the concentration range of 2.00 - 10.0 μM.

For detailed results of nonclinical pharmacokinetic and product metabolism studies of CYH33, see the IB [12].

1.2.3 Results of Nonclinical Toxicological Study

In the safety pharmacology study, CYH33 showed no significant drug-related changes in tidal volume (TV), respiratory volume (MV) per minute and frequency of respiration (Rf) of rats; Compared with the Beagle dogs, no drug-related arrhythmia or changes in ECG, blood pressure and body temperature parameters were observed in the rats; nor were effects on the central nervous system (CNS) noticed.

The acute toxicity assay confirmed that the MTD was ≥ 600 mg/kg in SD rats, and the maximum tolerated dose (MTD) was >120 mg/kg in beagle dogs. The long-term toxicity assay showed that the exposure and degree of toxicity in female SD rats at the same dose were greater than those in males, and the highest non-severely toxic dose (HNSTD) was 8 mg/kg in males and 3 mg/kg in females. The target organs were identified as hair follicles, immune/hematopoietic system, reproductive system (female rats) and pancreas. The most frequently affected organs of toxicity in Beagle dogs included skin/mucosa, immune/hematopoietic system, gastrointestinal tract, pancreas and (males) reproductive organ, with clear dose dependency. The highest non-severely toxic dose (HNSTD) was 0.2 mg/kg. These results suggest that close attention should be paid to the above target organ toxicities in clinical studies.

Fetal developmental toxicity assay showed slow weight gain in all the pregnant rats after administration of CYH33. At the dose of 0.30 mg/kg, live-birth rate, fetal weight, crown-rump length and tail length decreased; short/absent tail deformity, delayed ossification, skeletal malformation or variation were observed. The no-observed-adverse-effect level (NOAEL) for embryo-fetal development was 0.10 mg/kg. In addition, the salmonella typhimurium reverse mutation assay (Ames test), CHL chromosomal aberration assay and mice bone marrow micronucleus test all reported negative results.

For detailed results of CYH33 nonclinical toxicology studies, see the IB[12].

1.3 Clinical Benefit/Risk Analysis of Study Drug

This study is the first-in-human study of CYH33, and its safety and efficacy in humans are unknown.

In cell lines and PDX model in vitro, CYH33 showed strong antitumor activity against breast cancer and esophageal cancer, especially against esophageal cancer with high frequency PI3K activation. At the same dosage, CYH33 showed superior anti-tumor

activity over its counterpart candidate BYL719 under clinical study. Alpelisib (BYL719), an oral PI3K inhibitor developed by Novartis, potently and selectively inhibits PI3Kα, which has similar site and mechanism of action of CYH33. Several Phase Ia and Ib studies of BYL719 alone or in combination with letrozole, encorafenib and Erbitux showed that BYL719 was well tolerated and had preliminary efficacy in breast cancer (ER+ or ER+/HER2-) and colorectal cancer [15-17]. CYH33 may also have the potential to provide clinical benefit to treated patients.

CYH33 is a highly selective inhibitor of PI3K-AKt-mTOR signaling pathway, and its safety in humans is unknown. According to the preclinical safety evaluation data, we will pay close attention to the subject's immune, hematopoietic, reproductive, pancreatic, skin/mucosal systemic toxicity, blood glucose metabolism and cardiotoxicity in CYH33 clinical study, which may also be the adverse reactions requiring close attention in CYH33 clinical study. For the safety events occurred during the study, please refer to Section 5.12 for patient management.

1.4 Rationale of the Clinical Study

Calcaulation of Starting Dose

According to ICH-S9 (NONCLINICAL EVALUATION FOR ANTICANCER PHARMACEUTICALS) [14], for the selection of initial dose in clinical studies, interspecies dose conversion is usually performed by body surface area for most systemically acting small molecule drugs. One common practice is to use 1/10 of rodent STD10 as the initial dose. If a non-rodent is a closer species, 1/6 of its HNSTD (Highest Non-Severely Toxic Dose) may be selected as the initial dose. HNSTD is defined as the highest dose level that does not produce evidence of lethality, life-threatening toxicities or irreversible findings.

According to the results of long-term toxicological studies in nonclinical studies with CYH33, the HNSTD was 3 mg/kg in rats (females) and 0.2 mg/kg in dogs. The initial dose for human was calculated to be about 5 mg/person (60 kg) using 1/10 of the HNSTD in rats, 3 mg/kg; It was calculated to be about 1 mg/person (60 kg) using 1/6 of the HNSTD in dogs, 0.2 mg/kg; In contrast, a lower and safer dose at 1 mg/person was adopted as the initial dose of CYH33 in this clinical study, orally once daily.

CYH33 displayed a marked gender difference in its pharmacokinetics in rats, with a plasma clearance CL of 34.0 mL/min/kg in females and 4.83 mL/min/kg in males, respectively; After intragastric administration, CYH33 exposure AUC0-t in female rats was more than 12 times that in male rats; Elimination of CYH33 was slower in female rats ($t_{1/2}$ 6.7 hr) than in male rats ($t_{1/2}$ 1.3-4.5 hr); After intragastric administration, plasma exposure of amide hydrolyzed metabolite I27 in rats was approximately 2% (males) and 16% (females) of that of parent drug. In view of gender difference in pharmacokinetics in rats, only male subjects will be enrolled in rapid titration phase of this study, and based on PK data of male subjects, SMC will determine the dose for female subjects enrolled, and 1 subject will be enrolled to determine whether there is gender difference in PK parameters. Male and female subjects will be simultaneously enrolled in "3 + 3" escalation phase to observe the difference of PK parameters between genders.

In this study, based on the PK data from 1 mg dose group, the Safety Monitoring Committee (SMC) will discuss whether there is gender difference, and adjust the subsequent study design as needed.

Setting of Effective Dose to Human

According to the results from preclinical studies, CYH33 significantly inhibited the growth of these two subcutaneous xenografts at the dose of 12.5 mg/kg for 21 days in the subcutaneous xenograft models in nude mice derived from human esophageal cancer cell lines KYSE410 and KYSE510, with a T/C ratio of about 30% . The T/C ratio for patient-derived tumor xenograft (PDX) models EC104 and EC063 was about 30% when CYH33 12.5 mg/kg was orally administered for 21 days. Therefore, the effective dose of CYH33 in nude mice was determined at 12.5 mg/kg, and the converted human equivalent dose (HED) was approximately 60 mg.

Estimation of Critical Toxic Dose in Human

It was tentatively assumed that HNSTD would be a critical toxic dose for the occurrence of ≥ Grade 2 AE with a metabolic profile in human similar to that in rats. The long-term HNSTD of rats (females) was 3 mg/kg, converted to human equivalent dose (HED), and calculated as 3 mg/kg x 0.162 x 60 kg ≈ 30 mg.

It is expected that ≥ Grade 2 study drug-related AEs will begin to occur in the 30 mg group, and it is expected that at least 3 evaluable patients will be required for each group at the next dose to 30 mg. At that time, the Safety Monitoring Committee (SMC) will assess the safety, and determine the dose selection and number of enrolled cases for the subsequent study.

Rapid Titration + mTPI2 Design

The starting dose of the study is 1 mg/day, the expected effective dose is 60 mg/day, and the expected critical toxic dose in human is 30 mg/day. Therefore, in order to minimize the number of patients receiving lower than therapeutic concentrations, an rapid titration design will be adopted in several dose groups below 30 mg/day. In order to maximize patient protection, no dose increase is allowed for the same patient in the study, unless the investigator believes that a higher dose may be beneficial for the patient and it can be up-titrated to a higher dose, and the up-titrated new dose level must be assessed for its safety and not exceed the maximum tolerated dose (MTD).

In this study, based on the study data in the 1 mg dose group, SMC will discuss and decide whether a rapid titration design is adopted in the subsequent dose groups: 1 patient is enrolled in each dose group, and the next dose is escalated by 100% if no ≥ Grade 2 study-related AE occurs.

It is expected that ≥ Grade 2 AEs related to study drug will be observed in the 30 mg group. When DLT occurs for the first time or ≥ 2 subjects experience any study drug-related CTCAE Grade 2 toxicity (regardless of category) during the DLT observation phase, the dose level will be converted to mTPI2 design to guide toxicity monitoring and dose escalation. Safety Monitoring Committee (SMC) will assess the safety, and determine the dose selection and number of patients enrolled in the subsequent study.

Based on the results of nonclinical pharmacodynamic studies, T/C% in CYH33 50 mg/kg dose group is < 20% in the subcutaneous xenograft tumor model, approximately equivalent to a human dose of 240 mg. Therefore, the highest dose group is preset to 240 mg in the dose escalation phase. The actual highest dose level will be guided by PK/PD data simulation model and safety and efficacy of the enrolled subjects, as assessed by the SMC.

After the completion of DLT observation for each dose group, SMC will determine the dose, number of patients, or frequency of administration for the next dose group based on the safety, efficacy and PK data from the previous dose group. The SMC will determine the MTD and/or RP2D in light of the Phase Ia safety, efficacy and PK/PD data.

Selection of Subject Population

According to the results of nonclinical studies, it is decided that the safety, efficacy, and PK of CYH33 in patients with advanced solid tumors who have failed or cannot tolerate standard treatment or currently have no standard therapy will be investigated in Phase Ia.

[redacted]

The study will be conducted in compliance with the clinical protocol, Good Clinical Practice (GCP) and the applicable regulatory requirements.

2 Study Objectives and Endpoints

2.1 Study Objectives

Primary objectives:

- Phase Ia: to determine the safety, tolerability and maximum tolerated dose (MTD) of oral CTH33 monotherapy in patients with advanced solid tumors who have failed or cannot tolerate standard treatment or currently have no standard treatment, and determine the recommended Phase 2 dose (RP2D).

[redacted]

Secondary objectives:

- Phase Ia: to assess the preliminary efficacy of oral CTH33 monotherapy in patients with advanced solid tumors who have failed or cannot tolerate standard treatment or currently have no standard treatment

- Phase Ia: to determine the pharmacokinetic (PK) characteristics of CYH33 and its metabolite (I27) after single and continuous oral administration of CYH33.

[redacted]

[redacted]

[redacted]

Exploratory objective:

- To explore the relationship between the efficacy of CYH33 treatment and potential tumor biomarkers.

2.2 Study endpoints

Primary endpoints:

- Phase Ia: Type and frequency of treatment-emergent adverse events (TEAE), and evaluation of toxicity grades according to NCI CTCAE version 4.03; Clinically significant laboratory, electrocardiogram (ECG) and cardiac imaging findings and physical examination findings (including vital signs, weight and ECOG performance status score), etc.

- Phase Ia: Number and proportion of subjects with dose limiting toxicity (DLT) in DLT observation phase (within about 28 days after the first dose, 35 days after the first dose for patients receiving single dose: 7 days in the single dose phase + 28 days in the first cycle of continuous dose phase) [redacted]
- Phase Ia: Maximum tolerated dose (MTD); if MTD is not observed, the RP2D will be determined through PK (pharmacokinetic)/PD (pharmacodynamic) data, safety and preliminary efficacy.

[redacted]

Secondary endpoints:

- Phase Ia: Response assessment endpoints, including objective response rate (ORR), progression-free survival (PFS), duration of response (DoR) and disease control rate (DCR), clinical benefit rate (CBR).
- Phase Ia: PK parameters of CYH33 and its metabolite (I27), including:
    - Area under concentration-time curve from time point 0 (pre-dose) to last time point with measurable concentration ($AUC_{0\text{-last}}$)
    - Area under concentration-time curve from time 0 to 24 h ($AUC_{0\text{-24h}}$)
    - Area under concentration-time curve from time 0 (pre-dose) to infinity (∞) ($AUC_{0\text{-}\infty}$)
    - Maximum plasma concentration (C_{max})
    - Time to maximum plasma concentration (t_{max})
    - Elimination half-life ($t_{1/2}$)
    - Apparent volume of distribution (V_z/F)
    - Apparent clearance (CL/F)
    - Accumulation ratio of maximum plasma concentration (ARC_{max})
    - Accumulation ratio of area under concentration-time curve from time 0 to 24 h ($ARAUC_{0\text{-24h}}$)
    - Linearity index (LI)

[redacted]

[redacted]

[redacted]

Exploratory endpoint:

- The treatment response of CYH33 per tumor biomarker status (including but not limited to *PIK3CA*-alteration, *KRAS*, *BRAF* mutation, *AKT* alteration, etc.).

2.3 Definitions of Study Endpoints

DLT is defined as an adverse event or laboratory abnormality that occurs within 28 days after the first dose in Phase Ia (within 35 days after the first dose for patients receiving a single administration, i.e. 7 days of the single dose phase + 28 days of Cycle 1 of the continuous dose phase) and meets all of the criteria as shown in Table 1.

MTD is the maximum tolerated dose, defined as the maximum dose at which the incidence of DLTs is less than 33%. If necessary, MTD may be adjusted after the end of Phase Ib.

RP2D is the recommended Phase II dose and will be determined in light of Phase Ia safety, efficacy, and PK/PD data, regardless of MTD observed. If necessary, the RP2D can be adjusted in the course of the Phase Ib study.

Efficacy evaluation endpoints are determined according to guidelines (ICH E9, 1998, EMEA Guidelines, 2005 [6]). Patient's best response is defined as the best overall response from enrollment to end of treatment as judged by the investigator according to RECIST (Version 1.1).

ORR is defined as the proportion of patients with a second confirmed complete response (CR) or partial response (PR) as evaluated according to RECIST v1.1.

PFS is defined as time from the first dose of the study drug to disease progression or death, whichever is earlier. Patients without events (no progression or death) will be censored at date of the last tumor assessment. For patients lacking post-baseline efficacy evaluation, censoring will be made at date of the first dose.

OS is defined as overall survival time from the first dose of study drug to death for any reason. For patients without death event, censoring will be made at the last date when patient's survival information is obtained.

DoR is defined as the time from first CR or PR to tumor progression or death due to any reason, whichever occurs first. If no tumor progression or death is observed, censoring will be made at date of the last tumor assessment.

DCR is defined as the proportion of patients with confirmed CR, PR and SD lasting for ≥ 6 weeks.

CBR is defined as the proportion of patients with confirmed CR, PR and SD lasting for ≥ 24 weeks.

3 Study Plan

3.1 Overall Study Design

This is a multi-center, open-label, single-arm Phase I clinical study of dose escalation and dose expansion of oral CYH33 monotherapy in patients with advanced solid tumors who have failed or cannot tolerate standard treatment or currently have no standard treatment. The study population consists of patients with advanced solid tumors who have failed or cannot tolerate standard treatment or currently have no standard therapy in Phase Ia dose escalation part, patients with *PIK3CA*-mutated advanced solid tumors who have failed or cannot tolerate standard treatment or currently have no standard therapy in Phase Ia dose expansion part [redacted].

Patients must sign written informed consent and provide an unstained, radiation-naïve tumor tissue section, or/and formalin-fixed paraffin-embedded block or/and fresh tumor tissue to the central laboratory for confirming *PIK3CA* gene mutation status prior to screening.

This study includes Phase Ia dose escalation part, Phase Ia dose expansion part and Phase Ib. In Phase Ia, CYH33 will be observed to determine MTD in patients with advanced solid tumors who have failed or cannot tolerate standard treatment or currently have no standard treatment. If MTD is not observed, the recommended Phase II dose (RP2D) will be determined in light of Phase Ia safety, efficacy and PK/PD data. RP2D may be adjusted during Phase Ib study, if necessary. [redacted]

The phase Ia dose escalation part includes two dosing phases: single dose phase and continuous dose phase. Single dose phase (Day -1 to Day 7): Patients are orally administered one dose of CYH33 in the fasting state (fasting for at least 2 h before and after administration) and are observed for 7 days. If DLT does not occur in the observation phase, the patient enters into the continuous dose phase. Continuous dose phase (starting from Cycle 1): CYH33 will be orally administered once daily continuously in 28-day cycles under fasting state (fasting at least 2 h before and after administration) until disease progression, unacceptable toxicity, withdrawal of informed consent, withdrawal of the patient by the investigator or death. In these phases, the dose-limiting toxicity (DLT) observation phase is defined as Cycle 1 of the continuous dose phase (28 days), and for subjects receiving single dose, it lasts 35 days (i.e., 7 days in the single dose phase plus 28 days in Cycle 1 of the continuous dose phase);

According to the existing preclinical data, the initial dose is determined as 1 mg daily, and 1 evaluable male patient will be enrolled.

Based on the safety and PK data from 1 mg dose group, SMC will discuss and decide whether 1 male patient can be enrolled in the subsequent therapeutic dose or in each group.

Due to the low starting dose of 1 mg, the escalation will be performed as an ATD in order to avoid too many patients exposed to the therapeutic dose. One male patient will be enrolled per dose after 1 mg dose group. Then, according to the PK data in male subjects, the SMC will decide the dose for enrolled female subjects, and enroll 1 female subject to

determine whether there is any gender difference in PK parameters. When a study drug-related DLT is observed for the first time or any study drug-related CTCAE grade 2 toxicity (regardless of the type) is observed in ≥ 2 subjects in the DLT observation phase at a certain dose level, this dose level will be converted to guide toxicity monitoring and dose escalation by using the modified Toxicity Probability Interval method 2 (mTPI2).

Principle for dose escalation using mTPI2:

1. Three male or female subjects will be enrolled per dose group. If none of the 3 patients experiences DLT, the next schedule dose group will be opened to recruit 3 patients;
2. If 1 of the 3 patients experiences DLT, 2 more patients will be added to this dose group (see Figure 1: Dose Design Escalation Diagram mTPI2). Five patients will allow the decision-making on whether to step up or down a dose level;
3. If 2 of the 3 patients experience DLT on a dose level, additional participants will be entered at the next lower dose group;
4. The number of patients to be enrolled should be calculated based on Figure 1 from the number of existing patients at this dose and the number of DLTs. Each time no more than 3 patients will be enrolled. The SMC will decide when to stop enrollment for Phase Ia escalation.

DLT in Phase Ia is defined as an adverse event or laboratory abnormality that meets the definition and occurs within 35 days after the first dose (7 days in the single dose phase + 28 days in the Cycle 1 of continuous dose phase).

When DLT observation is completed for each dose group, SMC will evaluate the safety, efficacy data from the previous dose group and refer to existing PK data, and determine the dose and number of subjects in the next dose group; Meanwhile, the SMC will, based on sufficient PK data, decide as to whether the dosing frequency needs to be changed. If necessary, it is allowed to increase a new dose level between two explored dose levels for exploration. The MTD will eventually be determined.

MTD is defined as the maximum dose at which the incidence of DLT in Phase Ia is less than 33%; If the MTD is not observed, the RP2D will be determined by the SMC in light of the safety, efficacy and PK/PD data in Phase Ia. If necessary, the RP2D can be adjusted in the course of the Phase Ib study.

Figure 1 Schematic Diagram of mTPI2 Design Escalation [19]:

Number of Patients with DLTs

	1	2	3	4	5	6	7	8	9	10	11	12	13	14	15	16	17	18	19	20
0	E	E	E	E	E	E	E	E	E	E	E	E	E	E	E	E	E	E	E	E
1	D	D	S	S	E	E	E	E	E	E	E	E	E	E	E	E	E	E	E	E
2		D	D	D	D	S	S	S	E	E	E	E	E	E	E	E	E	E	E	E
3			DU	DU	D	D	D	D	S	S	S	S	E	E	E	E	E	E	E	E
4				DU	DU	DU	D	D	D	D	D	S	S	S	S	S	E	E	E	E
5					DU	DU	DU	DU	DU	D	D	D	D	D	S	S	S	S	S	S
6						DU	DU	DU	DU	DU	DU	D	D	D	D	D	D	S	S	S
7							DU	DU	DU	DU	DU	DU	DU	D	D	D	D	D	D	D
8								DU	DU	DU	DU	DU	DU	DU	DU	DU	D	D	D	D
9									DU	DU	DU	DU	DU	DU	DU	DU	DU	DU	DU	D
10										DU	DU	DU	DU	DU	DU	DU	DU	DU	DU	DU
11											DU	DU	DU	DU	DU	DU	DU	DU	DU	DU
12												DU	DU	DU	DU	DU	DU	DU	DU	DU
13													DU	DU	DU	DU	DU	DU	DU	DU
14														DU	DU	DU	DU	DU	DU	DU
15															DU	DU	DU	DU	DU	DU
16																DU	DU	DU	DU	DU
17																	DU	DU	DU	DU
18																		DU	DU	DU
19																			DU	DU
20																				DU

E: Escalate to the next higher dose; S: Stay at the same dose; D: De-escalate to the previous lower dose; DU: De-escalate to the previous lower dose and the current dose will never be used again in the trial;

Notes: The horizontal column represents the number of patients receiving this dose, and the vertical column represents the number of patients with DLTs at this dose. E: Escalate to the next higher dose S: Stay at the same dose D: De-escalate to the previous lower dose DU: De-escalate to the previous lower dose and the current dose will never be used again in the trial

In Phase Ia, a safe and considerably effective dose is selected for the Phase Ia expansion study, while the Phase Ia dose escalation study can continue synchronously. [redacted] At any dose level or if necessary, combined with the effective dose predicted with the PK/PD model, the SMC can decide to enter the expansion study at this dose level to further investigate the safety, tolerability and efficacy of this dose level. The subjects enrolled in the Phase Ia dose expansion part are patients with advanced solid tumors with *PIK3CA* mutations, and the total number of subjects for expansion will be decided by the SMC based on the proportions of patients with CR, PR and SD, as well as the safety and tolerability.

The rules for assignment of subjects awaiting enrollment to each dose group are as follows:

Subjects awaiting enrollment will be assigned to dose groups based on the number of cases required for each dose group, with priority given to subjects in the mTPI2 escalation phase.

When there are no less than two dose groups at the same time of expansion, the allocation ratio of the number of subjects in each dose group should be as close to the ratio of ORR in each dose group.

In principle, when the cumulative ORR at a certain dose level is <10%, the number of subjects in this dose group will be determined by SMC.

Approximately 1 subject will be enrolled at each dose level in ATD phase of Phase Ia dose escalation part, and at least 3 subjects will be enrolled at each dose level in the mTPI2 escalation part. A total of approximately 60 evaluable patients are expected to be enrolled. [redacted]

Principles for Dose Modification:

For patients in Phase Ia dose escalation part who develop any non-DLT AE during DLT observation phase, treatment may be discontinued depending on the grade of AE, but dose adjustment is not allowed; The occurrence of any protocol-specified DLT event requires discontinuation and is recorded as completing the study. For the remaining patients (including the patients who have completed DLT observation phase in the Phase Ia dose escalation part, as well as those [redacted] in the Phase Ia dose expansion part and all patients enrolled in phase Ib), no more than two dose reductions are allowed (the minimum dose is the initial dose), it is not allowed to resume the previous higher dose after a dose reduction. A patient will be permanently withdrawn from study treatment if the discontinuation due to an AE has been for more than 21 consecutive days. In these cases, the investigator may discuss with the sponsor whether to resume the treatment unless there is sufficient evidence that benefits and risks of the study treatment are manageable.

Dose up-regulation is not allowed for the same patient within the first 4 treatment cycles. After 4 treatment cycles, if the patient has not experienced any study-drug related CTCAE ≥ grade 2 toxicity, and the investigator considers that a higher dose may benefit the patient, the dose can be up-regulated to a higher dose, and the new dose level after up-regulation must have gone through safety evaluations and not exceed the maximum tolerated dose (MTD). The anti-tumor activity of CYH33 will be assessed in the study population per RECIST version 1.1. In Phase Ia and Phase Ib, the tumor assessment will be performed every 6 weeks (42 ± 7 days) in 28-day cycles from the first day of continuous dose phase until disease progression, unacceptable toxicity, death, or discontinuation study by investigator's decision or consent withdrawal of patients.

During the study, an SMC composed of site principal investigators enrolling subjects in the Phase Ia escalation part, independent clinical oncologists (if necessary), the sponsor's medical monitors and drug safety physicians will be established to review the safety, PK/PD and efficacy data arising from the study, decide the assignment of patients, dose levels and dosing frequency in each dose group in the Phase Ia dose escalation phase, and recommend and choose doses for entering the Phase Ia expansion part. After deciding the MTD, the SMC may evaluate whether other modes of administration are required (e.g.,

intermittent dosing). PK experts and biostatisticians, if necessary, may attend the meeting to provide advice, but have no right to vote.

End of study is defined as follows: the last enrolled patient completes two tumor assessments after the first dose, has disease progression or unacceptable toxicity, withdraws from the study or dies, whichever occurs first. By then, if some patients are still continuing the treatment, the drug will continue to be supplied until disease progression, unacceptable toxicity, patient withdrawal at the discretion of the investigator, or withdrawal of informed consent by the patient, or death.

See Figure 2 for dose escalation and expansion design, and see Appendixes 1 and 3 for study assessment list.

Figure 2 Schematic Diagram of Dose Escalation and Expansion Design

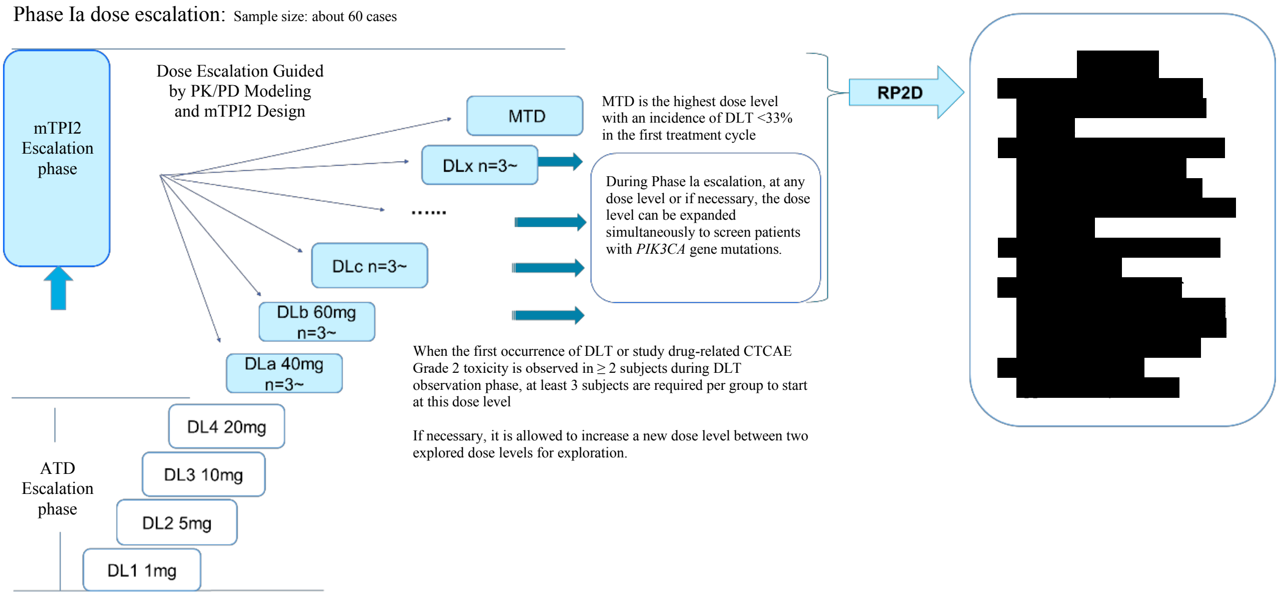

3.2 Phase Ia Dose Escalation Part

3.2.1 Single Dose Phase

Patients in the first 3 dose groups are orally administered one dose of CYH33 in the fasting state (fasting for at least 2 h before and after administration) and are observed for 7 days. If DLT does not occur in the observation phase, the patient enters into the continuous dose phase.

The necessity of a single dose phase or the length of the single dose phase for subsequent subjects will be decided by the SMC based on the PK data observed in the first 3 subjects.

3.2.2 Continuous Dose Phase

Patients are orally administered CYH33 in the fasting state (fasting for at least 2 h before and after administration), once daily for a 28 consecutive days to comprise one cycle until the end of study.

3.2.3 Dose-finding Principle

- According to the existing preclinical data, the initial dose is determined as 1 mg daily, and 1 evaluable male patient will be enrolled.

- According to the study data from 1 mg dose group, SMC will discuss and determine whether it may respectively enroll 1 patient in the subsequent several dose groups and adjust the observation phase after single dose, according to the following principles: If there is no ≥ Grade 2 study drug-related AE, the rapid titration is continued, enrolling 1 male subject per dose group; Then, according to the PK data in male subjects, the SMC will decide the dose for enrolled female subjects, and enroll 1 female subject to determine whether there is any gender difference in PK parameters. If there is gender difference, SMC will determine the dose groups and number of female subjects.
- When a study drug-related DLT is first observed or any study drug-related CTCAE Grade 2 toxicity (regardless of category) is observed in ≥ 2 subjects during DLT observation phase, the dose level will be converted to mTPI2 design to guide toxicity monitoring and dose escalation, and at least 3 evaluable subjects, both male and female patients, should be included at each dose level.
- When DLT observation is completed for each dose group, SMC will evaluate the safety, efficacy data from the previous dose group and refer to existing PK data, and determine the dose and number of subjects in the next dose group; Meanwhile, the SMC will, based on sufficient PK data, decide as to whether the dosing frequency needs to be changed. If necessary, it is allowed to increase a new dose level between two explored dose levels for exploration. The MTD will eventually be determined.
- The highest dose with an incidence of DLT less than 33% is considered as the MTD.
- The SMC will determine the RP2D in light of the Phase Ia safety, efficacy and PK/PD data.

3.2.4 Dose Escalation Adjustment

Currently, the dose escalation range is tentatively set as 1 - 240 mg. Dose escalation uses rapid titration design (ATD) plus mTPI2 design to guide toxicity monitoring and dose escalation mode. For the first two preset doses of 1 mg and 5 mg in ATD phase, if there is no ≥ Grade 2 study drug-related AE during DLT observation phase, 100% dose escalation is allowed in the subsequent dose groups. After entering mTPI2 phase, the SMC will determine the dose level, dosing frequency and number of subjects in the next dose group based on the safety and PK data of the dose group obtained previously. If necessary, it is allowed to increase a new dose level between two explored dose levels for exploration.

3.2.5 Intra-patient Dose Escalation

Dose up-regulation is not allowed for the same patient within the first 4 treatment cycles. After 4 treatment cycles, if the patient has not experienced any study-drug related CTCAE ≥ grade 2 toxicity, and the investigator considers that a higher dose may benefit the patient, the dose can be up-regulated to a higher dose, and the new dose level after up-regulation must have gone through safety evaluations and not exceed the maximum tolerated dose (MTD).

3.2.6 Definition and Evaluation of Dose-Limiting Toxicity (DLT)

DLT is defined as an adverse event or laboratory abnormality that occurs within 28 days after the first dose in Phase Ia (within 35 days after the first dose for patients receiving a

single administration, i.e., 7 days of the single dose phase + 28 days of Cycle 1 of the continuous dose phase) and meets all the following criteria:

- It is judged by the investigator and/or sponsor to be unrelated to the disease itself, disease progression, concomitant diseases or concomitant medication, and thus considered to be related to CYH33,
- and meets any of the criteria in the table below (unless otherwise specified, the NCI CTCAE version 4.03 criteria will be used for grading).

Table 1 Criteria for Dose-limiting Toxicity

Toxicity	Any of the following events will be regarded as DLT
Hematology	Grade 4 neutropenia (neutrophil count < 0.5×10^9/L);<br>Grade 3 thrombocytopenia (platelet count < 50×10^9/L) with a significant bleeding tendency, persisting for > 7 days, and failing to return to ≤ grade 2 or baseline level within 7 days after the study drug is suspended;<br>Grade 3 neutropenia with fever (neutrophil count <1.0×10^9/L, fever ≥ 38.5℃), persisting for > 7 days<br>Other grade 4 hematological toxicities
Hyperglycaemia	Grade 2 hyperglycemia (fasting blood glucose 8.9 -13.9 mmol/L or 160 mg/dL-250 mg/dL), and after appropriate anti-diabetic treatment (e.g., metformin, hypoglycemic sulfonylureas, etc.) while maintaining the current dose level of CYH33 treatment), the blood glucose fails to return to normal fasting blood glucose or baseline levels within ≤ 14 days,;<br>or Grade 3 hyperglycemia (fasting blood glucose 13.9 - 27.8 mmol/L or 250 - 500 mg/dL) or asymptomatic Grade 4 hyperglycemia (fasting blood glucose > 27.8 mmol/L or 500 mg/dL), which does not return to ≤ Grade 2 hyperglycemia within ≤ 7 days after suspension of CYH33 and adequate/appropriate anti-diabetic treatment;<br>or symptomatic (symptoms can include, but are not limited to, thirst, polydipsia, polyuria, fatigue, blurred vision, and even loss of consciousness) Grade 4 hyperglycemia (fasting blood glucose > 27.8 mmol/L or 500 mg/dL);<br>In all cases, fasting blood glucose should be retested within 24 h for confirmation.
Non-hematological toxicity	≥ Grade 3 non-hematological (except hyperglycemia) toxicities, except for the following conditions:<br>Nausea, vomiting, diarrhea, constipation and electrolyte imbalance which return to ≤ Grade 2 or baseline levels within 3 days after adequate/appropriate supportive care;<br>Fatigue persisting for ≤ 7 days after adequate/appropriate supportive care;<br>Nonclinically significant laboratory changes, which will not lead to discontinuation of the study drug per the investigator's judgment;<br>Grade 3 blood pressure increased, which can be spontaneously relieved or be controlled by medications to below 140/90mmHg or baseline levels within 3 days;
	Other clinically significant and unacceptable toxicity of any grade which requires termination of the study drug per the investigator's judgment, and will be determined by the SMC as DLT

3.2.7 Requirements for Enrollment Interval within One Dose Group

SMC will determine the dose for the next dose group based on safety data of the previous dose group (refer to the existing PK data), and adjust the interval time of subject enrollment in the next dose group.

3.2.8 Follow-up of DLT

Treatment discontinuation or permanent termination due to DLT should be followed once every week for 3 weeks, and once at least every 3 weeks afterwards, until the events are recovered or stable.

3.3 Phase Ia Dose Expansion Part

During Phase Ia study, the safe and highly powered effective dose will be delivered to the expansion cohort while the Phase Ia dose escalation study can be continued simultaneously. At any dose level or if necessary, combined with the effective dose predicted with the PK/PD model, the SMC can decide to enter the expansion study at this dose level to further investigate the safety, tolerability and efficacy of this dose level. The subjects to be enrolled in the expansion cohort are patients with advanced solid tumors with *P1K3CA* gene mutations, and the total number of subjects for expansion will be decided by the SMC based on the proportions of patients with CR, PR and SD, as well as on the safety and tolerability.

[redacted]

[redacted]

3.5 End of Study

End of study is defined as follows: the last enrolled patient completes two tumor assessments after the first dose, has disease progression or unacceptable toxicity, withdraws from the study or dies, whichever occurs first. By then, if some patients are still continuing the treatment, the drug will continue to be supplied until disease progression, unacceptable toxicity, patient withdrawal at the discretion of the investigator, or withdrawal of informed consent by the patient, or death.

3.6 Data Safety Monitoring Committee (SMC)

During the study, an SMC composed of site principal investigators enrolling subjects in the Phase Ia escalation part, independent clinical oncologists (if necessary), the sponsor's medical monitors and drug safety physicians will be established to review the safety, PK/PD and efficacy data arising from the study, decide the assignment of patients, dose levels and dosing frequency in each dose group in the Phase Ia dose escalation phase, and recommend and choose doses for entering the Phase Ia expansion part. After having determined the MTD/RP2D, the SMC may evaluate whether other modes of administration are required (e.g., intermittent dosing). PK experts and biostatisticians, if necessary, may attend the meeting to provide advice, but have no right to vote.

4 Selection of Subjects

Patients who meet all of the following inclusion criteria and do not meet any of the exclusion criteria can be included in this clinical study.

4.1 Inclusion Criteria

4.1.1 Inclusion Criteria for Molecular Screening

For Phase Ia dose extension part and Phase Ib, all of the following conditions must be met for patient enrollment:

1. Patients who sign the informed consent form for molecular screening before any study-specific operations.
2. Patients who provide diagnostic evidence for *PIK3CA* gene mutations.

   Evidence for *PIK3CA* gene mutations can be obtained through the following channels:

   - Pathological and genetic diagnosis reports of patient tumor tissue provided by existing medical/diagnostic institutions. These patients can enter into the clinical screening and subsequent study stages, and meanwhile, tumor tissue specimens (at least 10 unstained tumor tissue sections, or/and formalin-fixed paraffin-embedded pathological tumor tissue or/and fresh tumor tissue) and copies of the corresponding pathology reports and genetic diagnosis reports are sent to the designated screening laboratory for confirmation.

   - If no medical/diagnostic facility report demonstrates *PIK3CA* gene mutation in the patient's tumor tissue, the tumor tissue specimens (at least 10 unstained tumor tissue sections, or/and formalin-fixed paraffin-embedded tumor diseased tissue or/and fresh tumor tissue) and a copy of the corresponding pathology report must be forwarded to the designated screening laboratory for molecular screening. Each site can conduct the clinical screening of patients only when the *PIK3CA* genetic test result is available.

4.1.2 Clinical Screening Criteria for Inclusion

1. All of the following conditions must be met for patient enrollment: The patient signs the informed consent form (ICF) for clinical screening. The ICF must be signed before any study-specific operations.
2. Male or female patients ≥ 18 years old.
3. Phase Ia: patients with histopathologically or cytologically confirmed locally advanced or metastatic solid tumors who have failed or cannot tolerate standard treatment regimens or currently have no standard treatment regimen (including patients who reject any chemotherapy). [redacted]

[redacted]

4. In the screening phase, if agreed, patients can provide tumor tissue sections (unstained, approximately 4-30 sections); or/and formalin-fixed paraffin-embedded histopathological tumor samples (200 mm^3) and/or fresh tumor samples (20-150 mg/1-2 pieces of puncture biopsy tissue) and send them to the central laboratory for determination of responding tumor biomarkers.
   Note: Tumor tissue shall be obtained from radiotherapy-naïve sites.
5. An ECOG performance status score of 0 or 1.
6. Life expectancy ≥ 12 weeks.
7. Patients who can swallow tablets when enrolled in the study.
8. Patients with at least one measurable lesion assessed according to RECIST version 1.1.
   Note: A lesion previously treated by radiotherapy cannot be regarded as a target lesion, unless progression is documented after radiotherapy.
9. Patients' laboratory tests meet the following requirements to confirm adequate organ or hematopoietic system functions:
   Absolute neutrophil count (NEU) ≥ 1.5 × 10^9/L;
   Hemoglobin (HGB) ≥ 90 g/L (in the case of no blood transfusion in the past two weeks);
   Platelet (PLT) count < 90 × 10^9/L;
   Serum total bilirubin (TBIL) ≤ 1.5×ULN (if Gilbert syndrome is diagnosed, serum total bilirubin ≤ 3 × ULN is allowed but serum direct bilirubin (DBIL) must be ≤ 1×ULN);
   Aspartate aminotransferase (AST) and alanine aminotransferase (ALT) ≤ 2.5 × ULN (if there is liver metastasis, AST and ALT ≤ 5 × ULN are allowed);
   Serum creatinine (Scr) ≤ 1.5 × ULN; if there is an uncertain threshold, the creatinine clearance (Ccr) calculated should be ≥ 50 mL/min (calculated using the Cockcroft and Gault formula, see Appendix 8);
   International normalized ratio (INR) ≤ 1.5 or activated partial thromboplastin time (APTT) ≤ 1.5 × ULN;
   Blood potassium > 3.0 mmol/L;
   Fasting blood glucose (GLU) ≤ 126 mg/dL or ≤ 7.0 mmol/L;
   Glycated hemoglobin (HbA1c) ≤ 7.5%;
   Serum amylase (AMS) ≤ 1 × ULN;
   Serum lipase (LPS) ≤ 1 × ULN.
10. Patients who are able to follow the study procedures, restrictions and requirements at the investigator's discretion.
11. Both female and male patients of childbearing age must agree to use effective contraceptive measures from the date of signing the informed consent form to within 6 months after the last dose.

4.2 Exclusion Criteria

1. Patient's previous history of anti-tumor treatment complies with one of the following conditions:
    a. Patients who have received the last dose of any anti-cancer treatment (including chemotherapy, targeted therapy, biological therapy, or hormonal therapy, etc.) prior to the study drug administration less than 28 days or less than 5 half-lives (whichever is shorter) from the first dose of this study; Patients who have received traditional Chinese medicine for anti-tumor treatment (the indications on the package insert of the traditional Chinese medicine shall prevail) without a 14-day washout phase;
    b. Patients who have undergone major surgery within 28 days prior to the study drug administration or have not yet fully recovered from the previous surgery (in China, the definition of major surgery is based on grade 3 or 4 surgery defined in Management Measures for Clinical Application of Medical Technology implemented on May 1, 2009);
    c. Patients who have received radiotherapy (including whole brain radiotherapy) within 28 days prior to the study drug administration;
    d. Patients who have previously received and failed treatment with PI3K inhibitors, AKT inhibitors or mTOR inhibitors.
2. Patients have not yet recovered from the toxic reactions (except hair loss and pigmentation) caused by previous anti-tumor treatments (CTCAE > grade 1); Patients who have previously received platinum-based therapy, whose neurotoxicity needs to be restored to CTCAE ≤ grade 2; Patients who have not yet recovered to CTCAE grade 0-1 from the previous radiotherapy (except for permanent radiotherapy damage).
3. Patients with CNS metastases or malignant tumor-related epilepsy requiring clinical intervention.
    a. Patients with previously treated stable CNS metastases and asymptomatic central nervous system metastases can participate in the study. Patients must have completed topical treatment (including radiotherapy and/or surgery) for central nervous system metastasis ≥ 14 days prior to initiation of the study drug. Meanwhile, if the patient is receiving steroid treatment, the following criteria must be met:
        - The patient must be receiving a steady dose or is reducing the dose (≤ dexamethasone 4 mg/day or other steroids with the same anti-inflammatory effects).
        - The dose of steroids has not been increased for 14 days before the start of the study treatment.
4. Patients diagnosed with pancreatic cancer; Patients with concurrent malignancies or other malignancies within 5 years prior to the study recruitment (excluding properly treated basal or squamous cell carcinoma, non-melanoma skin cancer, or radically resected cervical carcinoma in situ).
5. Patients who have been treated with a granulocyte colony-stimulating factor within 14 days prior to the study drug administration.
6. Patients who have received a blood transfusion or platelet transfusion therapy within 14 days prior to the study drug administration.
   Note: Erythropoietin or erythropoietin treatment can be maintained if it has been used prior to recruitment.

7. Patients who are currently receiving medications known to have a risk of prolonging QT interval or inducing torsades ventricular tachycardia, and can neither stop the treatment nor switch to another drug prior to the start of the study drug administration.
8. Patients who are currently treated with warfarin or any other coumarin derivative.
9. Patients with known immediate or delayed hypersensitivity to the fellow drugs and excipients.
10. Patients who concurrently participate or have participated in another therapeutic clinical trial within 4 weeks prior to the first dose of the study drug. If the patient has participated in a non-interventional clinical trial (e.g., epidemiological studies) or is in the survival follow-up phase of an interventional clinical trial, the patient can be enrolled in this study.
11. Patients with hepatitis B virus (HBV) or hepatitis C virus (HCV) infection. All patients must receive HBV and HCV screening in the screening phase. HBsAG-positive patients can be enrolled in this study if HBV DNA is < 1,000 copies/ml or 200 IU/ml, but should take a monitoring of HBV DNA every 3 cycles throughout the study, and if necessary, the patient should receive antiviral treatment. HCV antibody-positive patients, if negative for HCV RNA tests, can be enrolled in this study.
12. Patients with human immunodeficiency virus (HIV) infection. All patients must receive HIV screening in the screening phase, and HIV-positive patients shall not be enrolled.
13. Patients' cardiac function and diseases meet one of the following conditions:
    a. During the screening phase, three electrocardiogram (ECG) measurements are performed at the study site, and the mean of the three measurements is calculated according to the instrument's QTc formula: male > 450 ms, and female QTc > 470 ms (corrected with the Fridericia's formula);
    b. Clinically significant arrhythmia, including but not limited to complete left bundle branch conduction abnormality, second-degree atrioventricular block, PR interval > 250 ms;
    c. Any risk factors that increase QTc prolongation, such as heart failure, hypokalemia, congenital QT prolongation syndrome, familial long QT syndrome or a family history of sudden death under 40 years of age, and administration of any medications prolonging the QT interval, etc.;
    d. ≥ Grade 3 congestive heart failure according to New York Heart Association (NYHA) classification;
    e. Unstable angina or new angina (within 3 months). Myocardial infarction occurring within 6 months prior to the study drug administration;
    f. Left ventricular ejection fraction (LVEF) < 50% based on echocardiography (ECHO) or multiple-gated acquisition scan (MUGA);
    g. Poorly controlled hypertension (defined as systolic blood pressure ≥ 140 mmHg and/or diastolic blood pressure ≥ 90 mmHg after optimal antihypertensive drug treatment) despite optimal drug intervention at the investigator's discretion;
    h. Patients who receive or need to receive placement of a pacemaker.
14. Arterial or venous thromboembolic events such as cerebrovascular accidents (including TIA), deep vein thrombosis or pulmonary embolism occurring within 6 months prior to the study drug administration.
15. Poorly healing wounds, ulcers or fractures.
16. Presence of serious clinically active infections (CTCAE > grade 2).

17. Presence of epilepsy that requires drug treatment.
18. Dysphagia, or ulcerative colitis, Crohn's disease; Or patients who have undergone small bowel resection (more than one-third of the small intestine has been resected); Or uncontrollable nausea, vomiting, diarrhea, malabsorption syndrome and other diseases which may significantly affect the administration or absorption of CYH33. Patients with a history of digestive tract perforation or obstruction may not be enrolled.
    Note: Patients with less than one-third of the small intestine resected can be enrolled in this study if their drug absorption is not affected at the investigator's discretion.
19. Patients with severe liver injury, end-stage liver diseases of Child-Pugh grade B and C.
20. Patients with a medical history or disease state of great clinical significance, or any disease state that is unstable or may affect the assessment of the study results or the safety or compliance of the subject at the investigator's discretion.
21. Pregnant or lactating women.
22. Patients with a history of acute or chronic pancreatitis or risk factors that may increase the risk of pancreatitis.

4.3 Criteria for Termination of Treatment

Patients have the right to withdraw from the study at any stage of the study. The investigator is permitted to terminate the treatment of patients due to the following or other reasons in advance. Patients may be discontinued from treatment in any study phase for any of the following reasons:

1. Disease progression
2. Death
3. Drug withdrawal for more than 21 days due to AE and any reason
4. DLT during the DLT observation phase
5. Pregnancy
6. Termination of treatment as determined by the investigator for the subject's best interests
7. Initiation of other anti-tumor therapy.
8. The patient or his/her legal representative requires withdrawal from the study
9. Lost to follow-up for more than 3 months
10. Serious violations to the study protocol: The patient does not meet the inclusion and exclusion criteria or has poor compliance. The patient's safety will be endangered if the patient continues to participate in the study at the discretion of investigator.
11. Termination of study upon the notification from the sponsor

4.3.1 Treatment of Patients who Discontinue Treatment

The reason for the termination of treatment should be recorded in the original medical record and eCRF, and the subsequent treatment after the termination of treatment should be recorded in the original medical record. If a patient prematurely discontinues treatment due to an AE, the investigator must follow the patient until the AE returns to normal or baseline, or until the investigator considers it unnecessary to follow the patient.

If a patient withdraws from the study or if the patient is unable to return for a visit, the investigator must confirm the primary reason for the patient's early withdrawal and record these information on the eCRF at the end of treatment. All patients who discontinue study treatment, including those who refuse to return for the last visit, will be contacted for safety evaluation at least 30 days after the last dose of study treatment.

[redacted]

4.3.2 Substitution of Patients who Discontinue Treatment

In the dose escalation part, due to non-DLT reasons, the patients who do not take the first dose as planned or the dose of the first cycle of continuous administration is less than 75% of the planned dose during DLT observation phase (within 28 days after the first dose, within 35 days after the first dose for patients receiving single dose, i.e., single dose phase and the first treatment cycle of continuous dose phase) should be replaced.

All study data will be included in the safety analysis for any reason of premature discontinuation.

4.4 Determination of Recommended Phase II Dose

The SMC will determine the RP2D in light of the Phase Ia safety, efficacy and PK/PD data. If necessary, the RP2D can be adjusted in the course of the Phase Ib study.

5 Study Drug and Study Method

5.1 Study Drug

CYH33 tablet is developed by Shanghai Institute of Materia Medica, Chinese Academy of Sciences. The sponsor, Shanghai Haihe Biopharma Co., Ltd., owns the clinical approval letter of CYH33 and all domestic rights, and serves as the research and development subject and sponsor of CYH33 clinical trials. CYH33 is available as scored tablets in strengths of 1 mg, 10 mg, and 50 mg for oral administration.

5.2 Packaging and Labeling of Study Drug

Drug label will be designed according to national requirements on drug label, and will include information on drug storage conditions, etc. but no patient's private information. Details of packaging and label template are shown in the clinical study file folder of the investigator.

5.3 Storage and Shipment of Study Drug

The sponsor will supply the study drug according to the recruitment plan of the site. The study drug will be shipped to the site by a third-party logistics company which is a qualified courier. Authorized site personnel will retain a copy of the signed shipping order and preserve it at the site.

The study drug should only be dispensed in this study. To fully control the distribution and use of study drug, the number of study drug should be recorded at each visit. At each on-site visit, the patient will need to return empty bottles and all unused drugs to the delegated site staff. All returned drugs cannot be redistributed. Throughout the study phase, all empty vials, returned drugs and other materials, are required to be recorded timely and precisely. The study drug will be collected and destroyed in a unified manner in accordance with relevant Chinese regulations.

5.4 Drug Management

The study drug should be kept in a secure area managed by specially-assigned personnel, protected from light, sealed and stored at normal temperature (10 - 30 °C). The investigator/pharmacist should be responsible for the storage, measurement of the temperature daily, and recording of the minimum and maximum temperature of each working day.

5.5 Drug Dispensing

All CYH33 tablets used in the study are dispensed to patients as prescribed by the investigator. Because the doses for patients in each dose group are different and CYH33 is available as scored tablets in strengths of 1 mg, 10 mg and 50 mg, the researchers will teach the subjects in person how to take the drug for the first time. Any change in dose must be recorded in the original medical record and CRF. The first dose after the dose change is given by researchers in person to teach the subjects how to take it.

5.6 Dose and Frequency of Administration

Single dose phase in phase Ia: Patients are orally administered one dose of CYH33 in the fasting state (fasting for at least 2 h before and after administration) and are observed for 7 days. If DLT does not occur, the patient enters into the continuous dose phase.

The necessity of a single dose phase or the length of the single dose phase for subsequent subjects will be decided by the SMC based on the PK data observed in the first 3 dose groups.

Phase Ia continuous dose phase and Phase Ib: Patients will take single or multiple doses of CYH33 daily in 28-day cycles under the fasting state (fasting at least 2 h before and after administration).

[redacted]

According to judgment of the investigator, if the patient obtains clinical benefit, the patient will take the study drug continuously until disease progression, unacceptable toxicity, death, or discontinuation study by investigator's decision or consent withdrawal of patients.

5.7 Method of Administration

Patients must take medicine in compliance with the following requirements:

1. Take medicine at the same time every day as much as possible, and during the PK blood collection phase, the patient must come to the hospital and take medicine under the instruction of the investigator or delegated site staff;
2. Each dose must be taken under fasting state (fasting for at least 2 h before and after administration) (e.g., the patient eats breakfast at 8:00 am, takes the drug at 10:00 am, and may not have lunch until 12:00 am); It is recommended to take CYH33 two hours before or after breakfast;
3. CYH33 tablets are administered with approximately 200 ml of warm water each time, and it is recommended to take the study drug within two minutes, with the time of swallowing the last tablet as the time of administration. It is recommended to finish drinking water within ten minutes;
4. Patients should swallow the whole tablet of study drug as much as possible;
5. During the single dose phase, if vomiting occurs within 15 minutes after administration, the drug should not be taken that day, but made up after three days. That day should be recorded as the administration phase. During the continuous dose phase, if vomiting occurs, it is not necessary to make up the dose until the time for the next dose; while it should be recorded in the eCRF;
6. If the patient misses dose in the morning, the missed dose can be made up before 22:00 pm on the same day. If the patient fails to make up the dose on the same day, the drug must be taken as required next time, but the missed dose will no longer be made up. Missed doses are recorded in the patient diary and in the eCRF by the investigator after review;

7. Patients should record the daily medication on the medication diary card (including episodes of vomiting at home), and return the diary card to the site staff when back to the hospital for visit.

On study days with fasting (overnight, 10 h) PK sampling prior to administration, the following additional guidelines should be followed:

8. Patients should be dosed in the clinic. Patients need permission to eat in the clinic;
9. On exam days involving both PK blood sampling and fasting glucose monitoring, patients must be fasting overnight and for at least 10 h prior to predose PK and fasting glucose collection. CYH33 is administered immediately;
10. On days of intensive PK blood sampling (i.e., Day 1 of the single dose phase, Day 1 (if any) and Day 28 of Cycle 1 of the continuous dose phase), no food should be consumed within four hours and no water should be consumed for at least one hour after CYH33 administration;
11. The predose PK sample collection time and CYH33 administration time must be accurately recorded in the eCRF. In addition, the last dosing time of CYH33 before PK sample collection must also be accurately recorded. If vomiting occurs within the first four hours of administration on the day of PK blood sample collection, the exact onset time of vomiting must be recorded. If vomiting occurs within the first four hours after administration on the day before PK sample collection, the exact onset time of vomiting must also be recorded, if possible;
12. During PK sample collection phase, avoid strenuous exercise, do not have tea, coffee and other caffeinated and alcoholic beverages, and do not smoke;
13. Please refer to the following order for visits at which PK blood sampling and other study procedures are required: study procedures, PK blood sampling before dosing, and then dosing.

The investigator should guide patients to take the study drug. All dose modifications for the study drug must be recorded in the specified drug record documents and input into eCRF. Diabetic patients receiving hypoglycemic drugs should be instructed by the investigator to make necessary adjustment of hypoglycemic regimen so as to avoid hypoglycemia.

5.8 Concomitant Medications

During the study phase, any drug (including supportive care) and non-drug therapies (including physical therapy or blood transfusion) other than the study drug must be recorded in the medical records and the CRF concomitant medication page. The anti-tumor therapies prior to enrollment, including medication, radiotherapy and surgery, should be recorded separately in the medical history.

If a patient takes nutritional supplements during the study, the investigator must be informed, and the sponsor and investigator should decide whether to record it as a concomitant medication.

5.9 Permitted and Prohibited Drugs and Non-drug Therapies

5.9.1 Permitted Drugs and Non-drug Therapies

1. Patients should receive sufficient supportive care during the study, including blood transfusion and blood products, antibiotic treatment, antiemetics, antidiarrheals, analgesics and other appropriate treatments consistent with guidelines in the institution.

2. Treatment with Bisphosphonates are supportive and permitted.

3. If enrolled patients are positive for HBV DNA, HBV DNA should be tested every 3 cycles. According to Guidelines for the Prevention, Care and Treatment of Persons with Chronic Hepatitis B Infection (2015): For HBeAg-positive patients with HBV DNA ≥ 20,000 IU/ml (equivalent to 105 copies/ml), and HBeAg negative patients with HBV DNA ≥ 2000 IU/ml (equivalent to 104 copies/ml), treatment with anti-HBV nucleoside drugs is required, and tenofovir and entecavir are preferred. Please refer to the guideline recommendations for details.

4. Low-dose aspirin therapy is allowed during the study. If anticoagulants containing heparin or low molecular weight heparin are required, it must first be discussed with the sponsor (with the exception of heparin lock).

5. If necessary, local radiotherapy is allowed for analgesic purposes or for lytic lesions at risk of fracture. If possible, such patients should undergo tumor assessment of lesions prior to radiotherapy to rule out disease progression. No dose adjustment of study drug is required during the radiotherapy.

6. If the patient requires the concomitant use of drugs involved in Appendix 11 that may induce torsades de pointes, the researchers may consider the concomitant use of these drugs. However, patients receiving concomitant medications should be closely monitored.

5.9.2 Prohibited Drugs and Non-drug Therapies

1 Patients should not receive other anti-cancer treatments (including but not limited to chemotherapy, radiotherapy, immunotherapy, biotherapy, other hormone therapy except hormone replacement therapy, surgery and/or tumor embolization) during the study treatment phase (from the first dose of the study drug to 30 days after the last dose). If a patient requires treatment with such medication, the patient must be withdrawn from the treatment phase prematurely.

2 Prophylactic use of hematopoietic colony-stimulating factors such as erythropoietin, granulocyte colony-stimulating factor (G-CSF), and granulocyte-macrophage colony-stimulating factor (GM-CSF) are not allowed. However, in acute events (e.g., acute myelosuppression with infection), hematopoietic growth factors may be administered at the discretion of the investigator, but the sponsor should be notified as soon as possible. Patients who start erythropoietin or erythropoietin prior to enrollment may continue the treatment at the discretion of the investigator.

3 Therapeutic doses of warfarin sodium or any other coumarin derivative anticoagulants are not allowed.

4 “Contraindicated QT-prolonging drugs" are not allowed. If the patient requires concomitant use of drugs listed in Appendix 10 “List of Contraindicated QT-prolonging Drugs", the study drug should be temporarily interrupted until the patient discontinues the use of QT-prolonging drugs.

5 Do not use herbal medications during the trial as potential drug interactions are always possible. These herbs include, but are not limited to: Hypericum perforatum extract, kava, ephedra, ginkgo biloba, dehydroepiandrosterone (DHEA), yohimbine, Qianlietong, and ginseng. Patients should stop taking these herbs at least 7 days before initiating the study treatment.

6 Hormonal contraception is contraindicated because it is not known whether hormones interact with CYH33.

7 CYH33 is metabolized by carboxylesterases, so foods and drugs that inhibit carboxylesterases are contraindicated, including alcohol, pyrethrins (pesticides), etc.

5.10 Overdosage

This study is the first-in-human study of CYH33, so there is insufficient data to demonstrate overdose of CYH33. Therefore, no range of overdose is defined in this project at present.

Currently, there is no known antidote for overdosage. Investigators are recommended to closely observe patients receiving doses exceeding scheduled dose and provide necessary supportive treatment and follow-up. The following measures are recommended for these patients:

- AEs/SAEs associated with overdosage must be recorded on relevant CRF AE/SAE pages.
- Overdose without AE/SAE should be recorded in overdose page of CRF, and the overdose and administration date should be recorded.

Once an event associated with overdosage is identified, the sponsor and CRO's relevant personnel must be notified within 24 h after awareness.

5.11 Treatment Compliance

The investigator should timely, promptly, and accurately record the number of study drug distributed to and returned by each patient and the actually administered dose and the specific dates, and the actually administered dose should be consistent with the dose specified in protocol. Compliance with drug treatment will be judged based on the number of drugs distributed to, returned and lost by the patients at the end of each treatment cycle and when the patients withdraw from the study. and the missed doses/overdose/lost drugs reported by patients as well.

The patients will be asked to return all the used and unused study drug bottles to the site at the end of treatment so as to evaluate their treatment compliance. All remaining materials and drugs must be returned to the sponsor at the end of study.

5.12 Principles for Treatment of Adverse Events During the Study

Among patients in the Phase Ia dose escalation part, if any DLT event stipulated in the protocol occurs within the DLT observation phase, i.e., within 28 days after the first dose (or within 35 days after the first dose for patients receiving a single administration, i.e. the

single dose phase plus Cycle 1 of the continuous dose phase), the drug should be immediately discontinued. The patient should complete the study and be withdrawn permanently in principle. The investigator may discuss with the sponsor whether to resume at the original dose or a reduced dose after discontinuation for DLT unless there is sufficient evidence that the patient has benefited from previous study treatment.

A maximum of two dose reductions (the lowest dose is the initial dose) is allowed for all patients (outside of Phase Ia DLT observation phase and Phase Ib). The dose reduction is determined by the investigator and sponsor based on the severity of AE and available safety and efficacy data, and re-escalation to the previous higher dose is not allowed after the dose reduction. If the administration is discontinued due to AE for more than 21 days, the patient should be permanently withdrawn from the study, unless there is enough evidence of treatment benefit. If the patient previously benefited from the treatment with CYH33, the investigator can discuss with the sponsor about whether the administration should be resumed 21 days after discontinuation.

Dose up-regulation is not allowed for the same patient within the first 4 treatment cycles. After 4 treatment cycles, if the patient has not experienced any study-drug related CTCAE ≥ grade 2 toxicity, and the investigator considers that a higher dose may benefit the patient, the dose can be up-regulated to a higher dose, and the new dose level after up-regulation must have gone through safety evaluations and not exceed the maximum tolerated dose (MTD).

Table 4 Criteria for Discontinuation, Resumption, Dose Reduction, and Permanent Discontinuation of CYH33

Recommended CYH33 dose modifications	
**Most Severe Toxicity (CTCAE Grade)**	**Recommended Dose Modifications**
**Hematology**	
**Neutropenia**	
Grade 1 (ANC < LLN - 1.5 x 10 ^{9}/L)<br>Grade 2 (ANC < 1.5 - 1.0 × 10 ^{9}/L)	Maintain the original dose
Grade 3 (ANC < 1.0 - 0.5 × 10 ^{9}/L)<br>Grade 4 (ANC < 0.5 × 10 ^{9}/L)	Withhold until recovery to ≤ Grade 1, and then:<br>Maintain the original dose if recovered within ≤ 7 days<br>Or if recovered within > 7 days, resume at the next lower dose
Febrile neutropenia (ANC <1.0 x 10 ^{9}/L with an oral temperature ≥ 38.3°C or body temperature ≥ 38.0°C on two consecutive occasions within two hours)	Discontinue<br>If the duration ≤ 7 days: Resume at a lower dose level.<br>If the duration > 7 days: Discontinue CYH33
**Thrombocytopenia**	
Grade 1 (PLT < LLN - 75 x 10 ^{9}/L)<br>Grade 2 (PLT < 75 - 50 × 10 ^{9}/L)	Maintain the original dose
Grade 3 (PLT < 50 - 25 × 10 ^{9}/L)	Withhold until recovery to ≤ Grade 1, and then:<br>Maintain the original dose if recovered within ≤ 7 days<br>If recovered at > 7 minutes, resume treatment at the next dose level.
Grade 4 (PLT < 25 × 10 ^{9}/L)	Withhold until recovery to ≤ Grade 1, then resume the treatment at a lower dose level.
**Kidney**	
**Blood creatinine**	

Recommended CYH33 dose modifications	
**Most Severe Toxicity (CTCAE Grade)**	**Recommended Dose Modifications**
<2 x ULN	Maintain the original dose
2 - 3 x ULN	Withhold until recovery to ≤ Grade 1, and then:<br>If recovered within ≤ 7 days, maintain the original dose to resume the treatment.<br>Patients should be instructed to increase their fluid intake before recovery to ≤ Grade 1.<br>If recovered at > 7 minutes, resume treatment at the next dose level.<br>Patients should be instructed to increase their fluid intake before recovery to ≤ Grade 1.
Grade 3 (>3.0 - 6.0 x ULN)	Discontinue CYH33 permanently
Grade 4 (> 6.0 × ULN)	Discontinue CYH33 permanently
**Liver**	
**Bilirubin**<br>(*For patients with Gilbert's syndrome, the following dose modifications are only applicable to changes in direct bilirubin levels)	
Grade 1 (> ULN - 1.5 x ULN)	Maintain the original dose, but monitor the liver function*
Grade 2 (> 1.5 - 3.0 × ULN) and ALT and AST ≤ 3.0 × ULN	Withhold until recovery to ≤ Grade 1, and then:<br>Maintain the original dose if recovered within ≤ 7 days<br>Or reduce the dose by one level if recovered within > 7 days
Grade 3 (> 3.0 - 10.0 × ULN) and ALT and AST ≤ 3.0 × ULN	Withhold until recovery to ≤ Grade 1, and then:<br>If recovered ≤ 7 days, reduce to a lower dose level<br>If recovered > 7 days, permanently discontinue CYH33
Grade 4 (> 10.0 × ULN)	Discontinue CYH33 permanently
**AST or ALT**	
Grade 1 (> ULN - 3.0 x ULN)	Maintain the original dose, but monitor the liver function*
Grade 2 (> 3.0 - 5.0 × ULN) and total bilirubin < 2.0 × ULN	Withhold until recovery to ≤ Grade 1, and then:<br>Maintain the original dose if recovered within ≤ 7 days<br>Or reduce the dose by one level if recovered within > 7 days
Grade 3 (> 5.0 - 20.0 × ULN) and total bilirubin < 2.0 × ULN	Withhold until recovery to ≤ Grade 1, and then:<br>Maintain the original dose if recovered within ≤ 7 days<br>Or reduce the dose by one level if recovered within > 7 days<br>Note: Except for baseline AST or ALT >3.0 - 5.0 ULN in patients with liver metastasis
Grade 4 (> 20.0 × ULN)	Withhold until recovery to ≤ Grade 1, and then resume at the next lower dose
**AST or ALT and Bilirubin**	
ALT or AST >3.0 × ULN and bilirubin >2.0 × ULN	Discontinue CYH33 permanently
*<u>**Liver function monitoring**</u><br>In the event of ALT, AST, or bilirubin increased ≥ **Grade 2**, liver function should be monitored **weekly** (or more frequently if clinically indicated) **until liver function recovers to ≤ Grade 1.**<br>In the event of ALT, AST, or bilirubin increased ≥ Grade 3 **,** liver function should be monitored **weekly** (or more frequently if clinically indicated) **until liver function recovers to ≤ Grade 1**; Resolved to ≤ Grade 1, liver function should be monitored every two weeks (more frequently as clinically indicated) **until the end of study treatment**.<br>Patients who discontinue the drug due to abnormal liver function should be monitored for liver function **weekly until liver function recovers to ≤ Grade 1 or reaches steady state** (without change in CTCAE grade for 4 consecutive weeks).	
**Fasting blood glucose (FBG)**	

Recommended CYH33 dose modifications	
**Most Severe Toxicity (CTCAE Grade)**	**Recommended Dose Modifications**
Grade 1 (> ULN - 160 mg/dL) [> ULN - 8.9 mmol/L]	Maintain the original dose and measure FBG weekly<br>Initiate or intensify appropriate antidiabetic therapy at the discretion of the investigator<br>Consider micromethod once daily for fasting or preprandial glucose monitoring<br>Measure FBG weekly for 8 consecutive weeks, and afterwards, biweekly
Grade 2 (> 160 - 250 mg/dL) [> 8.9 - 13.9 mmol/L]	**First occurrence:**<br>Maintain the original dose, repeat FBG within 24 h, and if not worsening to Grade 2 or above*:<br>Maintain the original dose<br>Initiate or intensify appropriate anti-diabetic therapy<br>Blood glucose monitoring by micromethod is recommended twice daily before breakfast and dinner. blood glucose at pre-lunch and bedtime may be additionally measured if necessary<br>If FBG does not return to ≤ Grade 1 within 14 days after initiation/intensification of appropriate antidiabetic therapy:<br>Discontinue CYH33<br>It is recommended that blood glucose monitoring (microdetermination) be performed twice daily, before breakfast and dinner, respectively. If necessarily indicated, additional pre-lunch and bedtime blood glucose measurements can be performed until FBG recovers to ≤ grade 1<br>Afterwards, CYH33 may be resumed at the next lower dose<br>Continue with the anti-diabetic therapy<br>Measure FBG weekly for 8 consecutive weeks, and afterwards, biweekly<br>* If there is a "worsening" of grade, recommendations for corresponding grades should be followed<br>**Second occurrence:**<br>Maintain the original dose, repeat FBG within 24 h, and if not worsening to Grade 2 or above*:<br>Discontinue CYH33<br>Initiate or intensify appropriate anti-diabetic therapy<br>It is recommended that blood glucose monitoring (microdetermination) be performed twice daily, before breakfast and dinner, respectively. If necessarily indicated, additional pre-lunch and bedtime blood glucose measurements can be performed until FBG recovers to ≤ grade 1<br>Afterwards, CYH33 may be resumed at the next lower dose<br>Continue with the anti-diabetic therapy<br>Measure FBG weekly for 8 consecutive weeks, and afterwards, biweekly<br>* If there is a "worsening" of grade, recommendations for corresponding grades should be followed
Grade 3 (> 250 - 500 mg/dL) [> 13.9 - 27.8 mmol/L]	**First occurrence:**

Recommended CYH33 dose modifications	
**Most Severe Toxicity (CTCAE Grade)**	**Recommended Dose Modifications**
	Terminate CYH33 immediately, initiate or intensify appropriate antidiabetic therapy, and repeat FBG within 24 h. If not worsening to Grade 3 or higher*:<br>Continuous discontinuation of CYH33<br>Blood glucose monitoring by micromethod is recommended four times daily before breakfast, lunch, dinner and bedtime until FBG recovers to ≤ Grade 1, and if recovered within 7 days, resume CYH33 at the original dose level. If recovered >7 days, reduce to a lower dose level<br>Continue with the anti-diabetic therapy<br>Measure FBG weekly for 8 consecutive weeks, and afterwards, biweekly<br>**Second occurrence:**<br>The treatment process is the same as the first occurrence, but the resumption of CYH33 is reduced to a lower dose level<br>* If there is a "worsening" of grade, recommendations for corresponding grades should be followed
Grade 4 (> 500 mg/dL) [> 27.8 mmol/L]	Terminate CYH33 immediately, initiate or intensify appropriate antidiabetic therapy, and repeat FBG within 24 h. If Grade 4 is confirmed:<br>Discontinue CYH33 permanently<br>Consider endocrinology consultation<br>Measure FBG weekly for 8 consecutive weeks, and afterwards, biweekly
**Pancreas**	
Grade 1 or 2 asymptomatic amylase or lipase elevated (1.0 - 2.0 x ULN)	Maintain the current dose
Grade 3 or higher asymptomatic amylase or lipase elevated (2.0 - 5.0 x ULN)	Withhold until recovery to ≤ Grade 2 and maintain the dose level if recovered ≤ 7 days; If recovered > 7 days, reduce to a lower dose level
Symptomatic (severe pain, vomiting, indication for medical intervention, life-threatening consequences) amylase or lipase elevated (any grade)	Discontinue CYH33 permanently
**Phototoxicity**	
Grade 1	Maintain the original dose<br>Initiate/intensify appropriate symptomatic treatment
Grade 2	Withhold until recovery to ≤ Grade 1, and then resume at the next lower dose
Grade 3 or 4	Discontinue CYH33 permanently
**Rash**	
Grade 1	Maintain the original dose<br>Consider to initiate appropriate symptomatic treatment (e.g., antihistamines, topical steroids)
Grade 2	Maintain the original dose<br>Initiate/intensify appropriate symptomatic treatment (e.g., antihistamines, topical steroids)
Grade 3	Withhold until recovery to ≤ Grade 1, and then:<br>Maintain the original dose if recovered within ≤ 7 days

Recommended CYH33 dose modifications	
**Most Severe Toxicity (CTCAE Grade)**	**Recommended Dose Modifications**
	If recovered >7 days (in the presence of appropriate symptomatic treatment), permanently discontinue CYH33
Grade 4	Discontinue CYH33 permanently
**Asthenia**	
Grade 1 or 2	Maintain the original dose
Grade 3	Withhold until recovery to ≤ Grade 1, and then:<br>Maintain the original dose if recovered within ≤ 7 days<br>Or reduce the dose by one level if recovered within > 7 days
Grade 4	Discontinue CYH33 permanently
**Other non-hematological adverse events**	
Grade 1 or 2	Maintain the original dose
Grade 3	Withhold until recovery to ≤ Grade 1, and then resume at the next lower dose
Grade 4	Discontinue CYH33 permanently

5.13 Monitoring and Management of Hyperglycemia in the Study

Insulin is the only hypoglycemic hormone in the body, involved in the regulation of carbohydrate, protein and lipid metabolism in the body, which acts by binding to the insulin receptor on the target tissue. PAM pathway covers several of the most important nodal molecules of insulin signaling pathway, so the use of PAM inhibitors brings about metabolic abnormalities, such as insulin resistance and elevated blood glucose.

Khurum K retrospectively compared the incidence of hyperglycemia in 341 patients in 12 Phase 1 clinical studies of PAM inhibitors conducted from 2007 to 2012 and found that 298 patients (87.4%) had elevated blood glucose, including 217 (72.8%) CTCAE G1, 61 (20.5%) CTCAE G2, and 20 (6.7%) CTCAE G3. It also found that predictors of Grade G3 or higher AEs included: Diabetes patients younger than 65 years; Maximum blood glucose greater than 8.5 mmol/L during C1 and use of AKT or PI3K/mTOR dual inhibitor (Khurum K. et al. The Oncologist 2016; 21:855-860). Another report at the same study site compared 18 PAMi Phase 1 clinical trials (387 cases) with 10 other Phase 1 clinical trials (non-PAMi, 109 cases) in advanced solid tumors (between 2008 and 2013). The results showed that the incidence of elevated blood glucose was not significantly different between the two groups (86.6% vs 80.7%), but severe hyperglycemia (G3-4) was significantly more common in the PAMi group than in the control group (6.7% vs 0%, P = 0.005). None of the patients with severe hyperglycemia developed acute complications of diabetes (diabetic ketoacidosis or hyperosmolar coma) after treatment with antidiabetic drugs, mainly metformin. (Geuna E. et al. British Journal of Cancer 2015; 113, 1541-1547). According to the 2012 NCI The PAM Task Force of the NCI Investigational Drug Steering Committee recommendations for the management of blood glucose in clinical studies of PAM inhibitors, for patients with elevated blood glucose between 125 - 160 mg/dL (CTCAE Grade 1) in the study, once daily fasting or preprandial blood glucose monitoring with micro-method can be considered, mainly including diet and exercise intervention, and hypoglycemic drugs when necessary; For asymptomatic patients with FBG between 160 - 250 mg/dL (CTCAE G2) and 250 - 500 mg/dL (CTCAE G3), micro-method blood glucose monitoring is performed twice daily before breakfast and dinner, blood glucose before lunch and at bedtime may be additionally measured if necessary, and oral medication and/or insulin regimen adjustment is actively performed; For patients with symptomatic CTCAE Grade 3 or asymptomatic FBG >500 mg, intravenous fluids should be considered, and blood glucose monitoring should be initiated four times daily to consider intensive insulin control; For patients with FBG above

500 mg/dL and combined symptoms, immediate endocrinologist consultation is required to give emergency treatment. Generally speaking, the academic community generally believes that the elevated blood glucose caused by PAMi is controllable. The main purpose of glycemic control in the study is to reduce the risk of acute complications of diabetes caused by acute hyperglycemia. The investigators can actively invite endocrinologists to manage blood glucose based on clinical judgment. J Clin Oncol 2012; 30:2919-2928

5.14 Female subjects and female partners of male patients must use highly effective contraception during the study and within 6 months after the last dose of study drug.

Any of the following can be defined as a highly effective contraception:

1. Absolute abstinence: This method should be compatible with the patient's preferred and usual lifestyle. Regular abstinence (e.g., calendar, ovulation, temperature signs, post-ovulation methods) and coitus interruptus are not accepted.
2. Sterilization: Patients have undergone oophorectomy with or without hysterectomy or tubal ligation at least six weeks previously. If oophorectomy is performed only, it may be applicable if the fertility status of the female patient has been confirmed by follow-up hormone level evaluation.
3. Male partner sterilization (there are appropriate post-vasectomy documentation of the absence of sperm in the ejaculation). [For female patients participating in the study, the vasectomized male partner should be the only sexual partner.]
4. The following combined approaches:
   a. Placement of intrauterine device (IUD) or intrauterine system (IUS)
   b. Barrier methods:
   - Contraceptive cap (plus spermicidal gel/cream) plus male condom
   - Diaphragm (plus spermicidal gel/cream) plus male condom

Note: Hormonal contraception (e.g., oral, injected, or implanted) are not allowed as the likelihood that the study drug may reduce the efficacy of hormonal contraception cannot be ruled out.

6 Study Procedures

6.1 Phase Ia

6.1.1 Molecular Screening Phase (Prior to the Clinical Screening Phase)

It is applicable to Phase Ia expansion part. All patients must provide the informed consent for molecular screening prior to any specific study assessments and procedures.

The complete medial history should be recorded at screening:

- Demographics including date of birth and sex

- Past history of cancer, including diagnosis of tumor and date of diagnosis
- Provide diagnostic evidence for *PIK3CA* gene mutations:

  Patients with a diagnostic report of *PIK3CA* gene mutation

  - Genetic diagnosis report
  - Tumor tissue specimens (at least 10 unstained tumor tissue sections, or/and formalin-fixed paraffin-embedded tumor pathology tissue or/and fresh tumor tissue)
  - Tumor pathology report

  Patients without a diagnostic report of *PIK3CA* gene mutation

  - Tumor tissue specimens (at least 10 unstained tumor tissue sections, or/and formalin-fixed paraffin-embedded tumor pathology tissue or/and fresh tumor tissue)
  - Tumor pathology report

6.1.2 Clinical Screening/Baseline Phase (From Day -28 to Day -1)

Before initiation of any study-specific evaluations or procedures, a written ICF for clinical screening must be obtained from each of the subjects.

The complete medial history should be recorded at screening:

- Demographic data: including date of birth, gender, race/ethnicity; alcohol history and smoking history;
- Previous medical history (all past medical histories including those occur before signing the ICF, and those considered associated with this study except for the indications).
- Past history of cancer, including date of diagnosis, start/end date of prior treatment regimen, best treatment outcome assessed, and date of disease progression; History of radiotherapy should include start/end date, site of radiotherapy. Previous significant operations (such as bronchoscopy, aspiration biopsy and other diagnostic or therapeutic invasive operations) should be recorded in eCRFs, including start and end dates, name of the operation and site where it is performed.
- If agreed, patients can provide tumor tissue sections (unstained, approximately 4 - 30 sections) prior to enrollment; or/and formalin-fixed paraffin-embedded histopathological tumor samples (200 mm^3) and/or fresh tumor samples (1 or 2 punch biopsy tissues 20 - 150 mg), which will be sent to the designated central laboratory for the determination of tumor biomarkers for response evaluation.

Stored formalin-fixed paraffin-embedded (FFPE) tumor tissue: paraffin-embedded blocks are highly recommended; however, if not available, approximately 4 - 30 paraffin-impregnated unstained sections should be provided for biomarker study at the designated central laboratory.

Fresh tumor biopsy tissues: 1 or 2 punch biopsy tissues of about 20 - 150 mg need to be collected from each patient, mainly including tumor tissues, rather than large normal tissues, stroma or necrotic tissues, which will be stored in liquid nitrogen or -80°C refrigerator immediately after collection and transported to the designated central laboratory immediately.

Note: The test items are determined according to the type of specimen collected and the quantity of accepted specimens. If an amount of tumor tissue samples satisfying the requirement of tumor biomarker exploration in this study has been collected, the collection can be stopped.

- Complete physical examination: Including height, body weight, head, eyes, ears, nose, larynx, neck, heart, thorax (including lungs), abdomen, limbs, skin, lymph nodes, nervous system and general condition of the patients.
- Vital signs: blood pressure, pulse rate, respiratory rate and body temperature; Blood pressure measurement should be performed on the patient after 5 minutes of rest in a sitting position.
- ECOG score: It is recommended that ECOG evaluation be performed by the same investigator throughout the study. See Appendix 5 for more details.
- Laboratory tests: specific laboratory tests include hematology, blood biochemistry (fasting for 10 h), urinalysis, coagulation function test, serum virology test and serum pregnancy test (if appropriate).
  - Hematology: red blood cell (RBC) count, HGB, hematocrit (HCT), MCH, MCHC, MCV, platelet count, WBC count and differential count including neutrophil count, lymphocyte count, monocyte count, basophil count and eosinophil count.
  - Blood biochemistry: including blood urea nitrogen/urea, creatinine, sodium, potassium, magnesium, chlorine, bicarbonate, calcium, phosphorus, blood sugar, total bilirubin, direct bilirubin, ALT, AST, alkaline phosphatase (ALP), lactic dehydrogenase (LDH), total cholesterol, total protein, albumin, fasting blood glucose (fasting for 10 h), HBA1c;
  - Serum amylase and lipase (LPS);
  - Urinalysis: including specific gravity, pH value, urine glucose, urine protein, ketone body, urine erythrocytes, urine leukocytes. If quantitative urine protein is ≥ 2+, quantitative 24-hour urine protein test will be performed.
  - Coagulation test (PT, INR and APTT).

- Serum pregnancy test (if appropriate)
- Serum virology: including tests on HBVsAg, HCV and HIV.

If HBsAg is positive, HBV DNA should be tested and repeated every 3 treatment cycles.

If anti-HCV antibody test reports positive, HCV RNA test needs to be performed.

This test can be exempted if a virological test report (with the normal reference intervals) issued by a formal medical institution within 28 days prior to the first dose (including prior to the signing of the ICF) is available.

- If there is an equivocal cut-off, creatinine clearance will be calculated using the Cockcroft and Gault formula
- Doppler Echocardiography or multiple uptake gated acquisition scan (MUGA).

  Throughout the study, it is recommended that the same technician uses the same instrument to complete a specific patient's LVEF assessment whenever possible
- 12-lead electrocardiogram (ECG).

  In rest and supine positions, 12-ECG is performed in triplicate to calculate the QTc interval (corrected using Fridericia's formula, see Appendix 9), and then calculate the mean value.
- Each ECG output should include at least 6 complex peaks, and is repeated once every 5 minutes, for 3 times in total. Patients should rest for at least 10 minutes before the start of ECG and be recorded in a supine position.
- Tumor evaluation: Tumor will be evaluated according to RECIST Version 1.1. See Appendix 6 for specific evaluation criteria. CT or MRI scan should be performed for thorax, abdomen, pelvis and any other sites with suspected tumor lesions at screening. Recording of target lesions: Number of lesions, sites, description, maximum diameter of each lesion and minimum diameter of lymph node, sum of diameters of all target lesions;
- Concomitant medication/treatment.
- AE/SAE assessment: AEs/SAEs will be recorded from the signing of informed consent until 30 days after the end of treatment (last dose). If a subject starts other systemic anti-tumor treatments, including but not limited to chemotherapy, radiotherapy, targeted therapy, immunotherapy, etc., except palliative radiotherapy for local alleviation of symptoms, within 30 days after the end of treatment, collection of AEs/SAEs will end at the start date of other systemic anti-tumor treatments.

6.1.3 Single Dose Phase (Day 1 to Day 7)

- Complete physical examination (only on Day 1).

- Vital signs (only on Day 1).
- ECOG score (only on Day 1).
- Laboratory tests: laboratory tests in the single dose phase should be performed within 7 days prior to Day 1 of the single dose phase (if relevant laboratory findings in the screening phase are not older than 7 days prior to Day 1 of the single dose phase, such findings can be used).
- Serum pregnancy test (if appropriate)
- Blood glucose monitoring (fasting for 10 h for fasting blood glucose): Peripheral circulating (fingertip) blood may be collected for test.
    - Day 1 (pre-dose, at 1 h and 24 h post-dose);
- 12-lead ECG (only on Day 1).
- UCG or MUGA (D1 can be exempted if the screening results are available within 14 days from D1).
- Concomitant medication/treatment.
- AE and SAE evaluations.
- DLT evaluation.
- PK blood sampling (Day 1 of single dose phase: pre-dose (within 15 minutes pre-dose), 0.5, 1, 2, 4, 8, 12, 24, 36, 48 and 72 h post-dose;).
- Dispensing and returning of study drug (only on Day 1).
- Blood sampling for pharmacodynamic biomarkers (within 2 h pre-dose, if available)

6.1.4 Continuous Dose Phase (First Cycle ± 2 Days; ± 3 Days of Cycle 2 and Each Subsequent Cycle)

- Complete physical examination: clinically indicated.
- Vital signs and ECOG score: Days 1, 8, 15 and 22 of Cycle 1, as well as Day 1 of each subsequent cycle.
- Laboratory tests (including hematology, blood biochemistry, urinalysis, coagulation function test, serum amylase and serum lipase): Days 1, 8, 15 and 22 of Cycle 1 and Day 1 of each subsequent cycle. (Screening lab findings may be used if the screening lab tests are still within 7 days prior to the first dose. Coagulation test: if the screening results are not clinically significant, the test does not need to be repeated on the day of the first dose and can be performed thereafter as clinically indicated. In addition, biochemistry test should be performed once on D1 and D15 in each treatment cycle from the second cycle. After four treatment cycles, it can be detected once on D1 in each treatment cycle according to clinical needs, until completion of early termination/end of treatment visit.)

- Fasting blood glucose monitoring (fasting for 10 h): Fasting blood glucose is included in blood biochemistry, and there is no need to additionally take blood to detect blood glucose; Peripheral circulating (fingertip) blood may be collected for test on days when blood chemistry is not monitored (pre-dose on C1D2, C2D8 and C2D22, etc.). For DLT assessment or if necessary, fasting blood glucose test should be repeated within 24 h. Unscheduled visits may be added if necessary.
    - Predose on days 1, 2, 8, 15 and 22 of Cycle 1;
    - Pre-dose on Days 1, 8, 15, and 22 of Cycle 2
    - Pre-dose on Day 1 of each subsequent treatment cycle;
    - With the adjustment of single dose phase, the blood sampling site for fasting blood glucose will be adjusted accordingly.
- 12-lead ECG: Days 1, 8, 15 and 22 of Cycle 1, as well as Day 1 of each subsequent cycle.
- Concomitant medication/treatment.
- AE and SAE evaluations.
- DLT evaluation (evaluation will be performed on C1D28, and a visit will be performed on C2D1 to review whether the potential AE qualifies as DLT, and to determine whether case replacement is required). Note: It is applicable to Phase Ia dose escalation and dose expansion part.

  PK blood sampling (if applicable, single dose phase: C1D1: predose (within 15 minutes before administration)); C1D8: pre-dose (within 15 min before administration); C1D15: pre-dose (within 15 min before administration); C1D28: pre-dose (within 15 min before administration), and 0.5, 1, 2, 3, 4, 6, 8, 12 and 24 h post-dose. If there is no single dose phase, directly enter the continuous dose phase: C1D1: predose (within 15 minutes before administration), 0.5, 1, 2, 3, 4, 6, 8, 12 and 24 h postdose; C1D8: pre-dose (within 15 min before administration); C1D15: pre-dose (within 15 min before administration); C1D28: pre-dose (within 15 min before administration), as well as 0.5, 1, 2, 3, 4, 6, 8, 12 and 24 h post-dose. With the adjustment of single dose phase, the PK blood sampling site are adjusted accordingly. Notes: It is applicable to all subjects in Phase Ia dose escalation part and some subjects in Phase Ia dose expansion part. It will be conducted in the phase Ia dose expansion part at the dose levels in some subjects selected by the SMC in light of the need of the PK study.
- Serum pregnancy test (Day 1 of each cycle, if applicable).
- Doppler Echocardiography or MUGA scan: Every 8 weeks (±7 days), or clinically indicated.
- Tumor assessment: performed every 6 weeks (± 7 days) from Day 1 of the continuous dose phase or as clinically indicated.

- Dispensing and returning of study drug: on Day 1 of each cycle.
- Blood sampling for pharmacodynamic biomarkers [predose (within 2 h) on C1D1, 4 h (± 30 min) postdose on C2D8, and CR, PR, and PD by tumor assessment, 8 ml of peripheral venous blood is collected and sent to the designated central laboratory. The samples for CR, PR and PD shall be no older than 7 days after the last tumor assessment]. (if any)

6.1.5 Early Termination/End of Treatment (Within 7 Days after End of the Last Dose ± 3 Days)

- Complete physical examination.
- Vital signs.
- ECOG score.
- Laboratory tests (including hematology, blood biochemistry, urinalysis, coagulation function, serum amylase and serum lipase test)
- 12-lead ECG.
- Concomitant medication/treatment.
- AE and SAE evaluations.
- Serum pregnancy test (if appropriate)
- Tumor assessment.
- Returning of study drug.

These test items (except for AE/SAE assessment and concomitant medication, concomitant therapy, drug recovery) can be exempted if the early withdrawal/end of treatment visit is within 14 days of the last visit assessment

6.1.6 Safety Visit (Within 30 Days ± 7 Days after the Last Dose)

- Complete physical examination.
- Vital signs.
- ECOG score.
- Laboratory tests (including hematology, blood biochemistry, urinalysis, coagulation function, serum amylase and serum lipase test)
- 12-lead electrocardiogram
- Concomitant medication/treatment.

- AE and SAE evaluations. If a subject starts other systemic anti-tumor treatments, including but not limited to chemotherapy, radiotherapy, targeted therapy, immunotherapy, etc., except palliative radiotherapy for local alleviation of symptoms, within 30 days after the end of treatment, collection of AEs/SAEs will end at the start date of other systemic anti-tumor treatments.
- Tumor assessment
- Other anti-tumor treatments

If a patient starts subsequent anti-tumor therapy within 30 days after the last dose, the safety follow-up visit should, if possible, be completed prior to the start of the new anti-tumor therapy.

[redacted]

[redacted]

[redacted]

[redacted]

[redacted]

[redacted]

[redacted]

[redacted]

[redacted]

[redacted]

[redacted]

[redacted]

[redacted]

[redacted]

[redacted]

[redacted]

[redacted]

[redacted]

[redacted]

[redacted]

[redacted]

[redacted]

[redacted]

[redacted]

[redacted]

[redacted]

[redacted]

[redacted]

[redacted]

[redacted]

[redacted]

[redacted]

[redacted]

[redacted]

[redacted]

[redacted]

[redacted]

[redacted]

[redacted]

[redacted]

[redacted]

[redacted]

[redacted]

[redacted]

[redacted]

[redacted]

[redacted]

[redacted]

[redacted]

[redacted]

[redacted]

[redacted]

[redacted]

[redacted]

7 Study Assessment

7.1 Safety Assessment

7.1.1 Safety Endpoints

- Number and proportion of subjects with DLT: Number and proportion of subjects with DLT occurring within 28 days after the first dose (within 35 days after the first dose in single dose phase).
- The incidence, type and severity of TEAE, which will be graded per NCI-CTCAE 4.03. Study-related AEs, SAEs, study-related SAEs, ≥ Grade 3 AEs and AEs leading to permanent discontinuation.
- The incidence and cause of death events within 30 days after the last dose.
- Safety laboratory tests categorized by NCI-CTCAE (Version 4.03).
- Vital signs, ECG, complete physical examination and ECOG performance status score.

7.1.2 Adverse Event Assessment

7.1.2.1 Definition of Adverse Event

- An adverse event (AE) is defined as any untoward medical occurrence (e.g., unfavorable and unanticipated sign, symptom, or disease (or worsening of pre-existing condition), abnormal laboratory findings) after signing informed consent form. Therefore, there may or may not be a temporal or causal relationship between the adverse event and the study drug. For patients with unknown *PIK3CA* gene mutation status who signed the informed consent form for molecular screening, AEs occurring after signing of ICF for molecular screening are only recorded on the AE page of eCRF if they meet the criteria for serious adverse events in 7.1.2.2 and are considered by the investigator to be causally related to study procedures (e.g., invasive procedures such as biopsy). After signing the informed consent for clinical screening, all AEs that meet the definition in this section should be recorded on the AE page of CRF. Events qualifying for the definition of AE include: Exacerbation of preexisting chronic or intermittent disease, including increased frequency and/or increased severity
- A disease newly discovered or diagnosed after study treatment, even though the disease might have existed before the start of the study
- Clinically significant signs, symptoms or clinical sequelea suspected resulting from drug interaction
- Clinically significant signs, symptoms or clinical sequelea suspected resulting from study treatment or overdosage of concomitant medication (the overdose will not be reported as AE/SAE).

“Lack of efficacy” or “Failure to reach the expected pharmacological effect” is not reported as AE or SAE. However, any clinical significant signs and symptoms and/or clinical sequelea caused by “lack of efficacy”, will be reported as AE or SAE if the definition of AE or SAE is satisfied.

Events that fail to meet definition of AE include:

- Medical or surgical procedure (e.g., endoscopy, appendectomy) scheduled before participation in this study.
- Circumstances where adverse medical events have not occurred (admission due to convenient social and/or environmental factors).
- Predictable fluctuations of the preexisting disease or condition which has already existed at the start of study and has not worsened during the study phase.
- Tumor progression or deterioration during the study (including new metastases and death due to disease progression) should be considered as a part of efficacy evaluation, and should not be reported as an adverse event or serious adverse event, unless the rate of progression is beyond our expectation.

7.1.2.2 Definition of Serious Adverse Event

An AE at any dose level is considered “serious” if it is:

a. Leading to death

b. Life-threatening

   Notes: The term "life-threatening" in the definition of "serious" refers to an event in which the patient was at risk of death at the time of the event; it does not refer to an event which hypothetically might have caused death if it were more severe.

c. Required or prolonged hospitalization

   Notes: In general, hospitalization signifies that the subject has been detained (usually involving at least an overnight stay) at the hospital or emergency ward for observation and/or treatment that would not have been appropriate in the physician's office or out-patient setting. A complication that occurred during hospitalization. If a complication prolongs hospitalization or satisfy any other serious criteria, the event is serious. When in doubt as to whether “hospitalization” occurred or was required, the AE should be considered serious.

   Hospitalization for elective treatment or a pre-existing condition that did not worsen from baseline is not considered as an AE.

d. Leading to permanent or significant disability/incapacity, or

Notes: The term disability means a substantial disruption of a person’s ability to conduct normal life functions. This definition is not intended to include experiences of relatively minor medical significance such as uncomplicated headache, nausea, vomiting, diarrhea, influenza, and accidental trauma (e.g. sprained ankle) which may interfere with or prevent everyday life functions but do not constitute a substantial disruption.

e. Congenital anomaly/birth defect

f. Medically significant or requiring intervention to prevent any of the above consequences, e.g., important medical events that may not be immediately life-threatening or result in death or hospitalization but may jeopardize the patient or may require medical or surgical intervention to prevent the other consequences listed in the definition above. These events should also be considered serious. Examples of such events are intensive treatment in an emergency room or at home for allergic bronchospasm, blood dyscrasias or convulsions that do not result in hospitalization.

7.1.2.3 Laboratory Test Abnormality and Other Safety Assessment Abnormality Reported as AE and SAE

Any abnormal laboratory findings (hematology, clinical biochemistry, urinalysis) that are considered clinically significant by the investigator, or other safety assessment (e.g., ECG, radiological scanning, vital sign measurement), including events that worsened from baseline and events of clinical significance by the investigator’s medical and scientific judgment will be reported as AE or SAE as per the definitions given.

In addition, any laboratory test abnormality or other safety assessment abnormality resulting in the medical or surgical intervention (including permanent discontinuation of study treatment, dose reduction and/or dosing interruption/delayed administration) will be recorded as AE or SAE.

Any newly diagnosed primary cancer must be reported as SAE.

7.1.2.4 Handling and Reporting of Adverse Events (AEs)

According to regulations of Chinese GCP, for all AEs occurred in the course of clinical study, regardless of the allocation of treatment group, or whether its causality to the study drug, the following measures should be taken:

1. The investigator should immediately take appropriate protective measures to ensure the patient’s safety, and experts should make a diagnosis and state reasons.
2. In case of study termination, the investigator should also regularly examine subjects and the date of study termination (the date when the dosing of the medicinal product is stopped), reasons for termination, and the detailed process should be recorded on eCRF.
3. The investigator should follow up all occurred AEs until any of the following situation occurs:
   - AE is relieved or improved to baseline status;
   - No further anticipated improvement will present according to the investigator;
   - Death of patients;

- Patient lost to follow-up;
- The end of the study.

Any AE should be recorded on eCRF in detail and reported in the clinical study report. The following variables will be collected for each AE:

- AE Term
- Onset and end time of AE
- CTCAE Grade
- An SAE or not
- Causality with the study drug
- Action taken for the study drug
- Outcome.

For SAEs, the following parameters will be further collected:

- Date of AE judged as SAE
- Date of the investigator aware of SAE
- Rationale for SAE
- Admission date and discharge date
- Possible cause of death, date of death, whether autopsy is performed, and other relevant important information of death
- Relationship to study procedures
- Causality assessment in relation to Other medication
- Description of AE.

AEs/SAEs are recorded from the signing of informed consent until 30 days after the last dose of study drug.

7.1.2.4.1 Causal Relationship between Adverse Events and Study Drug

The causal relationship between an AE and study drug is classified into two types as follows:

Related:

Adverse events that are considered related or possibly related to the investigational product. An adverse event is considered "related" if two or more of the following criteria are met:

1. The occurrence of an adverse event has a reasonable temporal relationship to drug

use.

2. An adverse event cannot be reasonably explained by known patient disease state, environmental or toxic factors, or other therapies used by the patient.
3. The adverse reactions will disappear or be alleviated after the dose is stopped or reduced. (There are some important exceptions, however, in which certain drug-related adverse reactions do not disappear even after drug withdrawal; such as: (1) myelosuppression, (2) tardive dyskinesia.)
4. The adverse event is consistent with the suspected drug reaction pattern.
5. Adverse event reappears upon re-challenge.

Not related:

Adverse events that are not considered related to the investigational drug. An adverse event is considered "unrelated" if two or more of the following criteria are met:

1. The occurrence of an adverse event does not have a reasonable temporal relationship to drug use.
2. The adverse event is clearly caused by the patient's disease state, environmental or toxic factors, or other concomitant therapies used by the patient.
3. The adverse event is not consistent with the suspect drug reaction patterns.
4. The AE is no longer present or worsening upon re-challenge.
5. The event is only caused by external factors (disease, environment, etc.).

7.1.2.4.2 Handling and Reporting of Serious Adverse Events (SAEs)

According to the regulations of Chinese GCP, for all SAEs that occurred in the course of the clinical study, in addition to treatment strategies for AE in Section 5.12, the following measures should also be taken:

1. The investigator should report to the CRA by telephone within 24 h, enter the adverse reaction page in eCRF within 24 h, and report to NMPA, drug regulatory authorities of relevant provinces, autonomous regions and municipalities directly under the central government, health administrative authorities, the sponsor and Ethics Committee in a written form, sign and date the report, and NMPA sends the SAE form to the sponsor for reference.

2. The investigator should provide additional information (the autopsy report, medical records of termination phase and other necessary information) of related SAEs (including death or adverse drug reactions which have been reported) as requested by the sponsor, medical institution and Ethics Committee.

3. The sponsor and the investigator should quickly evaluate the occurred SAE, and take necessary measures to ensure the safety and interests of subjects and report to the regulatory authorities, and other investigators of clinical study involving the same drug in a timely manner.

4. All SAEs should be followed up until resolution or improvement to baseline level, death or loss of contact. To ensure patient safety, every SAE, regardless of suspected causal relationship to study drug, that occurs from the signing of informed consent form to 30 days after the patient discontinues study drug must be reported according to Item 1 in this section. SAEs occurring more than 30 days after discontinuation of study drug will be reported to the sponsor only when the investigator considers a possible causal relationship between the event and study drug.

7.1.2.5 Pregnancy

For patient safety considerations, if a patient or the patient's spouse becomes pregnant during the treatment with study drug, it must be reported to the sponsor within 24 h after being informed. Female subjects should be discontinued immediately, and at the same time, the pregnancy must be followed to determine the pregnancy outcome, including spontaneous or induced abortion, details of delivery, congenital malformations, or maternal or neonatal comorbidities (assess the newborn within three months of birth).

In the event of pregnancy, the investigator should fill in the pregnancy report form, and record the details of pregnancy and its follow-up visit. If an SAE occurs during the pregnancy, SAE must also be reported and recorded on the SAE form.

7.1.2.6 Overdosage

If the patient develops symptoms due to overdosage (total daily dosage or use of the drug for a number of days more than that specified in protocol), the overdose and all related symptoms should be recorded on the eCRF AE page, and promptly reported to the CRO and the sponsor in no later than 24 h in accordance with the procedures of Section 5.10. Asymptomatic overdose should also be recorded on the overdose page of eCRF and reported in an expedited manner within 24 h. In the event of serious consequences or symptoms following overdosage are consistent with SAE assessment criteria, then the overdosage should be reported as SAE.

7.1.3 Laboratory Tests

All the following laboratory tests should be performed in the accredited local laboratories of the hospitals. Laboratory tests for unscheduled visits may be performed at a local laboratory. The normal ranges of all laboratory test items, and the update during the study should be recorded in the corresponding pages of eCRF.

Hematology: HGB, MCH, MCHC, MCV, platelet count, WBC count and differential count including neutrophil count, lymphocyte count, monocyte count, basophil count and eosinophil count.

Blood biochemistry: including fasting blood glucose, HBAlc, blood urea nitrogen/urea, creatinine, sodium, potassium, magnesium, chlorine, calcium, phosphorus, total bilirubin, direct bilirubin, ALT, AST, ALP, LDH, total cholesterol, total protein, and albumin.

Serum amylase and lipase (LPS).

Coagulation test includes PT, APTT and INR.

Urinalysis: Specific gravity, pH, urine glucose, urine protein, ketone body and urine red blood cells, urine white blood cells. If quantitative urine protein is ≥ 2+, quantitative 24-hour urine protein test will be performed.

7.1.4 Other Safety Evaluation

Physical examination: including height (only at screening), body weight, head, eyes, ears, nose, larynx, neck, heart, thorax (including lungs), abdomen, limbs, skin, lymph nodes, nervous system and the patient's general condition.

Vital signs: including blood pressure, pulse rate, respiratory rate and body temperature. Blood pressure measurement should be performed on the patient after 5 minutes of rest in a sitting position.

ECOG performance status score: It is recommended that ECOG performance status evaluation should be performed by the same investigator throughout the study.

ECG: It will be performed before measurement of blood pressure.

NYHA classification: Doppler Echocardiography or MUGA scan will be used to examine left ventricular ejection fraction. The above tests will only be performed at screening.

7.2 Efficacy Evaluation

Tumor assessment will be performed according to RECIST 1.1 (See Appendix 6 for specific evaluation criteria). CT or MRI can be used for tumor imaging at the investigator's discretion. However, the evaluation method, machines and technical parameters used should remain consistent throughout the study; Contrast agents should be used if not contraindicated. Imaging results will be interpreted by site investigators or radiologists. Reader should not be changed throughout the study. If tumor evaluation has been performed within 28 days prior to the first dose using the same method and machine in the same hospital, the results can be used as baseline tumor evaluation. Baseline tumor assessment should include thorax, abdomen, pelvis and any other suspected sites of tumor lesions. If clinically indicated, appropriate method can be used to examine any other known or sites with suspected lesion, for example, bone scan or neck CT scan.

Recording of target lesions: Number, sites and description of lesions, maximum diameter of each lesion (except lymph nodes), minimum diameter of lymph node and sum of diameters of all target lesions.

From C1D1 of the continuous dose phase, tumor assessment will be performed on the patients every 6 weeks (± 7 days) by means of imaging until disease progression, unacceptable toxicity, death, or discontinuation study by investigator's decision or consent withdrawal of patients. The investigator may schedule additional imaging examinations based on the patient's clinical condition. Confirmatory assessment must be completed within 6 weeks after efficacy is evaluated as CR or PR (per RECIST Version 1.1). If the patient discontinues treatment due to AE or other reasons, tumor evaluation will still be performed as scheduled. If the patient withdraws from the study due to disease progression, it is not necessary to repeat imaging assessment at the last visit. For patients with suspected disease progression before the next scheduled assessment, unscheduled tumor assessment should be performed.

7.3 Pharmacokinetic Assessment

7.3.1 Blood Sample Collection

To detect plasma concentrations and PK profiles of unchanged CYH33 and its potential metabolite (I27) in patients in Phase Ia, plasma samples are collected at the following time points:

If there is a single dose phase:

Single dose phase

- Day 1: pre-dose (within 15 mins before administration), and at 0.5, 1 2, 4, 8 and 12 h post-dose.
- Day 2: 24 h and 36 h post-dose.
- Day 3: 48 h and 72 h post-dose.

Continuous dose phase

- Cycle 1 Day 1 (C1D1): pre-dose (within 15 min before administration);
- C1D8: pre-dose (within 15 minutes before administration).
- C1D15: pre-dose (within 15 minutes before administration).
- C1D28: pre-dose (within 15 min before administration), and at 0.5, 1, 2, 3, 4, 6, 8, 12 and 24 h post-dose. Among these time points, the 24-hour blood samples on C1D28 will be collected within 15 min prior to the dosing on C2D1.

The necessity of a single dose phase or the length of the single dose phase for subsequent subjects will be decided by the SMC based on the PK data observed in the first 3 dose groups. With the change of single dose phase, SMC decision is allowed to make corresponding change to the blood collection site in the subsequent continuous dose phase.

If there is no single dose phase, subjects will directly enter the continuous dose phase:

PK blood collection points: C1D1: pre-dose (within 15 min before administration), and 0.5, 1, 2, 3, 4, 6, 8, 12 and 24 h post-dose; C1D8: pre-dose (within 15 min before administration); C1D15: pre-dose (within 15 min before administration); C1D28: pre-dose (within 15 min before administration), and 0.5, 1, 2, 3, 4, 6, 8, 12 and 24 h post-dose.

Among these points, the 24 h blood samples on C1D1 and C1D28 will be collected within 15 min before administration on C1D2 and C2D1.
The PK study will be carried out in the Phase Ia dose escalation part, and will be conducted at selected dose levels in some subjects in the PhaseIa dose expansion part.

2 mL venous blood will be collected from upper limb at each time point of blood draw, centrifuged at 3000 g for 10 min. Upper layer of plasma is transferred to 2 labeled cryogenic vials, marked using a permanent marker. The collected plasma samples should be stored in a refrigerator at ≤ -70°C within 30 mins.

During the study, no more than about 48 mL (with single dose phase) or 44 mL (without single dose phase, direct continuous dose phase) blood samples should be collected from

each patient for PK analysis. Indwelling needles may be used by the site to collect blood samples as appropriate. See separate laboratory manual for specific procedures of blood collection, storage conditions and transport instructions.

Moreover, in case of overdosage, hepatic toxicity (if appropriate) or ocular phototoxicity, additional 2 ml venous blood will be collected to determine drug concentration.

For details regarding the PK blood collection schedule and the volume of blood collection, see Appendix 2 and Appendix 4.

Plasma concentrations of CYH33 and its primary metabolite (I27) will be determined by the designated central laboratory. Results will be presented in the form of a biological analysis report.

The actual time of blood collection for each blood sample and all issues related to the collection and handling of blood samples should be recorded in the CRF.

7.3.2 Pharmacokinetic Endpoints

Plasma PK parameters of CYH33 and its metabolites in the dose groups will be calculated using non-compartment model. PK parameters include:

Phase Ia:

1. Single dose phase:
   $AUC_{0-\infty}$, AUC_{0-last}, $AUC_{(0-24h)}$, C_{max}, t_{max}, $t_{1/2}$, CL/F, V_z/F.
2. Continuous dose phase:
   $AUC_{(0-24h)}$, C_{max}, t_{max}, ARC_{max}, $ARAUC_{(0-24h)}$, LI.

7.3.3 Blood Sample Handling and Transport

The following information should be clearly indicated for plasma samples:

- Protocol No. (CYH33-101)
- Patient Number
- Theoretical sample collection cycle and time
- Actual sample collection time
- Two collection tubes with barcode identification.

Contents on sample label should be inerasable. Two tubes of samples from each collection point are sent on dry ice to the designated central laboratory and stored at ≤ -70°C. Before completion of the final study report, all remaining samples will be stored at ≤ -70°C, and can only be destroyed after the confirmation by sponsor.

[redacted]

[redacted]

[redacted]

[redacted]

[redacted]

[redacted]

[redacted]

[redacted]

[redacted]

[redacted]

[redacted]

[redacted]

[redacted]

[redacted]

[redacted]

[redacted]

[redacted]

[redacted]

[redacted]	[redacted]	[redacted]	[redacted]	[redacted]	[redacted]
[redacted]	[redacted]	[redacted]	[redacted]	[redacted]	[redacted]
[redacted]	[redacted]	[redacted]	[redacted]	[redacted]	

[redacted]

8 Data Management

8.1 Data Entry

Electronic data capture (EDC) system will be used for this study. Electronic case report forms (eCRFs) are used to record all protocol-required and investigator-collected data. The sponsor or its designated team will develop the eCRF according to the specific needs of study and will train the investigator or its designated personnel on the eCRF. After completion of the subject's medical records, the investigator or designee of his/her team enters the data at the site. After entry, data will be transferred from the site to the study database via the Internet. For all patients who signed the ICF, the investigator or authorized staff must carefully record the items in the eCRF in details; All data in the eCRF must be checked with the patient's source data to ensure the correctness.
The investigator should complete the eCRF in strict accordance with the instructions for completion.

8.2 Database Lock

When the following conditions are satisfied, the data can be locked:

1. All data have been entered into database;
2. All cleaning work has been completed and all queries have been resolved;
3. SDV and electronic signature of all data have been completed;
4. Medical coding is completed;
5. Data consistency check between external data and trial database has been completed;
6. The analysis population has been defined and evaluated.

The locked data files will not be revised further without the authorization of the sponsor.

After the database is locked, the investigator will receive a CD containing the subject data of his/her site, which will be incorporated in the study file.

9 Statistics and Statistical Analysis

Specific statistical methods will be detailed in the separate Statistical Analysis Plan. Descriptive statistical analysis will be mainly applied in this study. All statistical analyses will be calculated by the sponsor or its designated team using SAS statistical analysis software.

In general, continuous variables (such as age) will be statistically described using the observed value, mean, median, standard deviation, quartiles, minimum and maximum; Categorical variables will be statistically described using the frequency and percentage of each category. The final analysis of study will be based on data of the patients collected during the entire study phase.

9.1 Sample Size

There are approximately 60 evaluable patients in Phase Ia [redacted]. The actual number of subjects and dose escalation level will be discussed and decided by SMC based on the obtained safety data and/or PK/PD modeling simulation data.

9.2 Analysis Sets

DLT analysis set (only applicable to Phase Ia): patients who experienced DLT within 28 days after the first dose (within 35 days after the first dose for patients receiving a single administration), or patients who have used >=75% of the study drug within 28 days of Cycle 1 of the continuous dose phase as planned.

Full analysis set: enrolled patients who have taken at least one dose of CYH33. The full analysis set will be applied to efficacy assessments.

Evaluable analysis set: patients who have taken at least one dose of CYH33 with baseline tumor assessment data and data of post-baseline tumor assessments at least once.

Safety analysis set: patients who have taken at least one dose of CYH33.

PK analysis set: including patients who have received at least one dose of CYH33 and have at least one evaluable PK data. Subjects with protocol violations seriously affecting the PK assessment results will be excluded from the PK analysis set.

PD analysis set: including all patients who have received one dose of CYH33 and have evaluable PD data (without major protocol violation that may affect the PD data analysis). PD analysis set will be applied to the primary efficacy assessment of PD data.

9.3 Safety Analysis

Safety will be evaluated by summarizing DLTs, AEs, changes in laboratory test results and changes in vital signs.

In Phase Ia, all TEAEs (treatment-emergent AEs), i.e., AE occurring during the treatment phase (from the first dose to the last dose + 37 days) or AE that is present at baseline but worsen after the administration of the drug, will be summarized.

AEs will be summarized by system organ class and preferred term during the single dose phase, and treatment-related AEs, SAEs, treatment-related SAEs, ≥ Grade 3 AEs, and AEs leading to drug withdrawal will be summarized.

Changes in laboratory test results will be summarized according to grading by the NCI-CTCAE version 4.03 criteria. For laboratory indicators, the maximum toxicity occurring in the study will be summarized using number and percentage. Changes in vital sign and ECOG scores will be compared with baseline levels and descriptive statistical analysis will be performed.

In phase Ia study, results of safety data analysis and PK data analysis will be regularly submitted to the SMC independent of the study to evaluate the safety of the study drug. The specific submission time will depend on project progress.

9.4 Efficacy Analysis

Study ORR is defined as the proportion of patients with CR or PR according to RECIST V1.1, where CR and PR should be confirmed by a second tumor assessment within 42 ± 7 days. ORR (proportion of patients with CR and PR) will be descriptively analyzed and 90% Clopper -Pearson confidence interval will be calculated. Survival analysis will be performed for PFS and DoR by using the Kaplan-Meier curve according to the investigator's assessment results, and descriptive statistical analysis will be performed for DCR and CBR. [redacted]

Exploratory analyses will also be performed for the following subgroups, if possible:

- Age: >65 years or <65 years
- Pathological stage: IIIb, IIIc, IV and others
- Surgical history: Yes, No
- History of chemoradiotherapy: Yes, No

9.5 PK Analysis

PK data will be analyzed using PK set based on the collected individual concentration-time data of CYH33 from the single dose phase to the continuous dose phase. Non-compartmental model in Phoenix® WinNonlin 6.4 or higher (Pharsight Corp., Certara, Princeton, New Jersey, United States) or SAS® 9.2 or higher (SAS Institute, Inc., Cary, North Carolina, United States) will be used for analysis of PK parameters. Actual blood sample collection time will be used in calculation of final plasma concentration. PK analysis will strictly follow the Standard Operating Procedures (SOPs) of the sponsor or its designated party. PK parameters include:

Phase Ia:

1. Single dose phase:
   $AUC_{0-\infty}$, AUC_{0-last}, $AUC_{(0-24h)}$, C_{max}, t_{max}, $t_{1/2}$, CL/F and V_z/F.
2. Continuous dose phase:
   $AUC_{(0-24h)}$, C_{max}, t_{max}, ARC_{max}, $ARAUC_{(0-24h)}$ and LI.

Tabulation and descriptive statistics will be used to summarize individual and mean plasma concentrations of CYH33 and its metabolites at each sampling point, including arithmetic mean, minimum, median, maximum, standard deviation, coefficient of variation and geometric coefficient of variation. Moreover, scatter plot will be provided for individual and mean plasma concentrations of CYH33 at each sampling point. PK parameters will be statistically analyzed by arithmetic mean, geometric mean, median, reference range, standard deviation and coefficient of variation. Details will be provided in the statistical analysis plan. If other PK parameters have to be calculated, details will also be provided in statistical plan.

Preliminary analysis data will be sent to relevant personnel responsible for PK methodological validation, and interpretation of results . The final results will be transferred to data management system.

[redacted]

[redacted]

[redacted]

[redacted]

9.7 Interim Analysis

Not applicable.

9.8 Final Analysis

The primary analysis will be performed after the last patient in Ib has completed the 6-month treatment to summarize the safety and efficacy data. After the database is locked, the efficacy data and safety data are further analyzed.

10 Study Management

This study will be performed in strict compliance with laws and regulations on clinical studies in China, including GCP, Declaration of Helsinki (Version 2013) and other regulations, as well as the study protocol. Specific study procedures will be subject to standard operating procedures of each participating site.

10.1 Ethical Considerations

The study protocol, ICF, eCRF and other relevant materials must be submitted to the IEC for review and approval before the start of this study. IEC will review and approve these materials in strict compliance with relevant laws and regulations, and issue the approval document after approval. The study can only be started only after the approval document is obtained from IEC.

During the study, any revision of protocol must also be reviewed and approved by IEC before implementation.

10.2 Informed Consent

The investigator or designated representative will be responsible for explaining study background, pharmacological features of study drug, study protocol and benefits and risks of participation in the study to each patient, the patient's legal representative or a fair witness, and obtain written informed consent form signed by the patient or legal representative and study physician before the patient is included in the study (before screening examination).

The final ICF text should contain the following contents: study objectives, study procedures, patient's obligations, foreseeable benefits of participation to patients, and foreseeable risks and inconveniences; treatment available to the patient and appropriate insurance

compensation in the event of study-related injury; access to study data and confidentiality of patient information. ICF should obtain written approval from relevant regulatory authority according to regulations and be written in language that is readable to patients.

The patient or legal representative, and the investigator or representative administering the process of informed consent must sign and date ICF. An original copy of the ICF should be kept by the investigator and patient respectively. If new important data involved in investigational drug are found, ICF must be revised and then submitted to relevant regulatory authorities in written form for review and approval. The informed consent should be re-obtained.

10.3 Compensation for Health Damage of Subjects

If a subject experiences an injury causally related to participation in this study, the sponsor will bear the treatment expense through the insurance bought for this clinical trial and offer corresponding economic compensation in accordance with relevant national laws and regulations. For damage due to medical malpractice or failure to comply with the study protocol, the sponsor will not provide compensation.

10.4 Recording and Retention of Study Data

In order to ensure the evaluation and supervision by NMPA and the sponsor, the investigator should agree to retain all the study materials, including confirmed records of all subjects (by which all recorded data can be effectively checked, such as eCRF and original records in hospital), all original ICFs signed by the subjects, all eCRFs, detailed drug dispensing records. The documents should be retained for 5 years after the conclusion of the trial or to the time of the sponsor's notice on destroying the data.

All materials regarding this clinical study are proprietary to the sponsor. Without prior explicit written permission of the sponsor, the investigator may not provide such materials to any third party in any form, except for as required by the NMPA.

10.5 Return or Destruction of Investigational Product(s)/Therapeutic Products

After the conclusion of the study, all clinical investigational products/therapeutic products should be returned to the sponsor, or destroyed in the study site according to the written instruction of the sponsor.

The destruction of investigational products/therapeutic products in the study site should be performed in the presence of representative of the sponsor or by appropriate personnel delegated by the sponsor. If the destruction of investigational products/therapeutic products is performed by the delegated personnel, a formal signed certificate of destruction should be filled out and submitted to the sponsor.

10.6 Quality Control and Quality Assurance

In order to ensure the quality of this study, the clinical study protocol will be discussed and developed together by the sponsor and/or CRO and the investigators prior to the start of this study. GCP training will be provided for relevant study personnel involved in the study.

The management of the study drug (including receipt, storage, dispensing and return) at each site must be conducted according to SOP.

10.6.1 Monitoring and Auditing

10.6.1.1 Monitoring

CRAs authorized by the sponsor have the right to consult eCRF, ICF, and all of the original data at any time.

CRA will be responsible for formulating the plans and procedures needed for monitoring this study. Before study starts, the on-site visit will be conducted. Regular monitoring visits should be conducted during study execution. If necessary, the monitoring can be conducted by phone, fax or e-mail, as a supplement of on-site inspection.

Before the study starts, the investigator will be informed of the expected frequency of monitoring visit. In addition, during the study, the investigators will be notified in advance prior to each monitoring visit. The purpose of visits is to ensure that the clinical study is carried out in strict compliance with the study protocol; completeness and accuracy of CRF, and can be confirmed from source documents.

The CRA should verify that all CRFs are completed correctly and completely, and are consistent with the original data; It should be also confirmed that any errors or omissions are corrected or indicated, and signed and dated by the investigator. At each visit, the investigator should cooperate with the CRA closely for the purpose of review and confirmation of the eCRFs, drug supply and inventory records, drug dispensing and return records and any other scheduled additional documentation.

The sponsor or authorized person of the sponsor may review the study quality, and the reviewers have the right to inspect all study-related medical records, the investigators' folders and correspondence, and ICFs.

10.6.2 Audits and Inspections

The auditor may perform an audit of this study at the appropriate time to ensure that this study is conducted in compliance with the relevant national laws and regulations and the protocol. After audit, the auditor will provide a written report to describe the identified issues. Related personnel (investigators and the CRA) should take appropriate and document corrective actions.

10.7 Amendments to Study Protocol

Any important changes to the protocol must be approved by the sponsor and the investigator in writing, and the amendments should be submitted to IEC for review and approval, and should be filed in accordance with local regulations.

10.8 Protocol Violation

Once the subject who deviates obviously from certain criteria in protocol is inadvertently included into the group, the subject should be withdrawn from the study.

The investigator should ask the patient at every visit whether there is concomitant medication since the last visit, in order to determine whether there is protocol violation in this treatment and the information should be recorded at the same time.

10.9 Study Termination

The sponsor retains the right to stop the study at any time for medical reasons or any other reasons. For early termination or suspension of study, the sponsor shall immediately notify the investigator of termination or suspension of the study, and explain the reasons. The sponsor or the investigator shall immediately notify the IEC of the termination or suspension of the study as well according to relevant laws and regulations, and the written statement should be provided.

The investigator has the right to determine whether the study should be stopped. If the investigator terminates or suspends the study without obtaining the sponsor's consent in advance, the investigator should notify the sponsor and IEC immediately, and provide a detailed written statement for the sponsor and the IEC on the study termination or suspension. The study records must be retained.

10.10 Study Report

After the conclusion of this study, the sponsor and the investigators will summarize the results objectively, and conduct statistical analysis on study data with appropriate statistical methods, and perform objective assessment on the drug safety based on the results. A written study report will be developed after obtaining the sponsor's approval upon review.

10.11 Confidentiality and Publication of Study Results

The investigator should keep information and data of this study confidential. Without the consent of the sponsor, study-related results or data shall not be cited or published.

The sponsor has the right to publish the study-related information or data, or submit them to NMPA. If the sponsor needs to include the investigator's name in a published paper, publication or advertisement, the consent of the investigator should be obtained.

11 References

1. Chen, W., et al., Cancer statistics in China, 2015. CA Cancer J Clin, 2016. **66**(2): p. 115-32.
2. Shikha SS，Wei NY，Frank A，et al．Targeting the PI3KAkt signaling pathway in gastric carcinoma A reality for personalized medicine．World J Gastroenterol，2015，21(43) : 12261-12273．
3. Paul AH，Colleen LM,Ramachandran M．Allosteric modulation of Ras and the PI3K/AKt /mTOR pathway: emerging therapeutic opportunities．Frontiers in Physiology，2014，(5) : 478．
4. Cancer Genome Atlas Network. Comprehensive molecular portraits of human breast tumour. Nature，2012，490（7418）：61-70.
5. Cancer Genome Atlas Research Network. Comprehensive molecular profiling of lung adenocarcinoma. Nature，2014，511（7511）：543-550.
6. Cancer Genome Atlas Research Network，Kandoth C，Schultz N，et al. Integrated genomic characterization of endometrial carcinoma . Nature，2013，497（7447）：67-73.
7. Hollander MC，Blumenthal GM，Dennis PA. PTEN loss in the continuum of common cancers，rare syndromes and mouse models. Nat Rev Cancer，2011，11（4）：289-301.
8. Carpten JD，Faber AL，Horn C，et al. A transforming mutation in the pleckstrin homology domain of AKT1 in cancer. Nature，2007，448（7152）：439-444.
9. Riener MO, BawoM M, Chvien PA．Rare *PIK3CA* hotspot mutations in carcinomas of the biliary tract．Genes Chronmsomes Cancer，2008，47(5)：363-367．
10. Pandurangan AK，Esa NM. Potential targets for prevention of colorectal cancer：a focus on PI3K/Akt/mTOR and Wnt pathways . Asian Pac J Cancer Prev，2013，14（4）：2201-2205.
11. 2012 Chinese Cancer Registry Annual Report
12. Shanghai Haihe Biopharma Co., Ltd., Investigator's Brochure for CYH33, Version 1.0.
13. Center for Drug Evaluation, National Medical Products Administration, Guidelines for Clinical Trials of Antitumor Drugs.
14. CPMP/ICH, *ICH Topic E9 – Statistical Principles for Clinical Trials: Consensus guideline finalised (step 4)* 1998.
15. Dejan Juric, et.al; Abstract CT-01: BYL719, a next generation PI3K alpha specific inhibitor: Preliminary safety, PK, and efficacy results from the first-in-human study; 1538-7445.AM2012-CT-01.
16. IA Mayer, and et. al;A Phase Ib Study of Alpelisib (BYL719), a PI3Kα-specific Inhibitor, with Letrozole in ER+/HER2-Negative Metastatic Breast Cancer; Clin Cancer Res. 2016 Apr 28. pii: clincanres.0134.2016.
17. Josep Tabernero and et.al; Combination of Encorafenib and Cetuximab With or Without Alpelisib in Patients With Advanced BRAF-Mutant (BRAFm) Colorectal Cancer: Phase 2 Results（poster European Society for Medical Oncology18th World Congress on Gastrointestinal Cancer June 29–July 2, 2016 Barcelona, Spain）.
18. Data from C-bioportal-2019-01.
19. Guo W, Wang SJ, Yang S, Lynn H, Ji Y.A Bayesian interval dose-finding design addressingOckham's razor: mTPI-2. Contemp Clin Trials. 2017 Jul; 58:23-33.

12 Appendices

Appendix 1.1 List of Phase Ia Assessments (Single Dose Phase + Continuous Dose Phase)

	Clinical screening phase D-28-D-1	Single-dose phase[1]		Continuous dose phase[1]												Early termination/end of treatment Within ≤ 7 days after the last dose of the study drug (± 3 days)	Safety Follow-up 30 days after the last dose of study drug (± 7 days)
				C1						C2				C3 and subsequent cycles			
		D1	D2-D7	C1D1 (± 2 days)	C1D2	C1D8 (± 2 days)	C1D15 (± 2 days)	C1D22 (± 2 days)	C1D28 (± 2 days)	C2D1 (± 3 days)	C2D8	C2D15 (± 3 days)	C2D22 (± 3 days)	CXD1 (± 3 days)	CXD15 (± 3 days)		
ICF for clinical screening	X																
Demographics	X																
Inclusion/exclusion criteria evaluation	X																
Past medical history	X																
Tumor history	X																
Serum β HCG test for women of childbearing age	X	X[15]		X[15]						X				X		X[14]	
Tumor assessment (RECIST Version 1.1)[2]	X			Every 6 weeks ± 7 days from Day 1 of the continuous dose phase													
Complete physical examination[3]	X	X		X						X				X		X[14]	X

	Clinical screening phase D-28-D-1	Single-dose phase[1]		Continuous dose phase[1]												Early termination/end of treatment Within ≤ 7 days after the last dose of the study drug (± 3 days)	Safety Follow-up 30 days after the last dose of study drug (± 7 days)
				C1						C2				C3 and subsequent cycles			
		D1	D2-D7	C1D1 (± 2 days)	C1D2	C1D8 (± 2 days)	C1D15 (± 2 days)	C1D22 (± 2 days)	C1D28 (± 2 days)	C2D1 (± 3 days)	C2D8	C2D15 (± 3 days)	C2D22 (± 3 days)	CXD1 (± 3 days)	CXD15 (± 3 days)		
Vital signs and ECOG score [4]	X	X		X		X	X	X		X				X		X[14]	X
Echocardiography or MUGA[5]	X	X [15]		X [15]	Every 8 weeks ± 7 days from Day 1 of the continuous dose phase												
12-lead electrocardiogram	X	X		X		X	X	X		X				X		X[14]	X
Hematology[6]	X	X[16]		X[16]		X	X	X		X				X		X[14]	X
Blood biochemistry[19]	X	X[16]		X[16]		X	X	X		X		X		X	X	X[14]	X
HbA1c	X																
Blood glucose [17]	X	X		X	X	X	X	X		X	X	X	X	X		X[14]	X
Serum virology[7]	X																
Coagulation test (PT, APTT, INR) [8]	X	X[16]		X[16]												X[14]	X
Serum amylase and lipase (LPS)	X	X[16]		X[16]		X	X	X		X				X		X[14]	X
Urinalysis[9]	X	X[16]		X[16]		X	X	X		X				X		X[14]	X
Concomitant medication/treatment[10]	X	X															X

<table>
<tr><th rowspan="3"></th><th rowspan="3">Clinical screening phase
D-28-D-1</th><th colspan="2" rowspan="2">Single-dose phase[1]</th><th colspan="12">Continuous dose phase[1]</th><th rowspan="3">Early termination/end of treatment
Within ≤ 7 days after the last dose of the study drug
(± 3 days)</th><th rowspan="3">Safety Follow-up
30 days after the last dose of study drug
(± 7 days)</th></tr>
<tr><th colspan="6">C1</th><th colspan="4">C2</th><th colspan="2">C3 and subsequent cycles</th></tr>
<tr><th>D1</th><th>D2-D7</th><th>C1D1
(± 2 days)</th><th>C1D2</th><th>C1D8
(± 2 days)</th><th>C1D15
(± 2 days)</th><th>C1D22
(± 2 days)</th><th>C1D28
(± 2 days)</th><th>C2D1
(± 3 days)</th><th>C2D8</th><th>C2D15
(± 3 days)</th><th>C2D22
(± 3 days)</th><th>CXD1
(± 3 days)</th><th>CXD15
(± 3 days)</th></tr>
<tr><td>AE and SAE evaluation[11]</td><td>X</td><td colspan="15">X</td><td>X</td></tr>
<tr><td>DLT evaluation</td><td></td><td colspan="2">X</td><td colspan="6">X</td><td></td><td></td><td></td><td></td><td></td><td></td><td></td><td></td></tr>
<tr><td>PK blood collection[12]</td><td></td><td>X</td><td>X</td><td>X</td><td></td><td>X</td><td>X</td><td></td><td>X</td><td></td><td></td><td></td><td></td><td></td><td></td><td></td><td></td></tr>
<tr><td>Tumor tissue samples[13]</td><td>X</td><td></td><td></td><td></td><td></td><td></td><td></td><td></td><td></td><td></td><td></td><td></td><td></td><td></td><td></td><td></td><td></td></tr>
<tr><td>Blood collection for PD biomarker exploration [18]</td><td></td><td>X</td><td></td><td>X</td><td></td><td></td><td></td><td></td><td></td><td></td><td>X</td><td></td><td></td><td></td><td></td><td></td><td></td></tr>
<tr><td>Dispensing and returning of study drug</td><td></td><td>X</td><td></td><td>X</td><td></td><td></td><td></td><td></td><td></td><td>X</td><td></td><td></td><td></td><td>X</td><td></td><td>X</td><td></td></tr>
</table>

Abbreviations: β-hCG = beta-human chorionic gonadotropin; AE = adverse event; ALT = alanine aminotransferase; APTT = activated partial thromboplastin time; AST = aspartate aminotransferase; CR = complete response; CT = computed tomography; CXDX = Day X of Cycle X; DLT = dose-limiting toxicity; ECOG = Eastern Cooperative Oncology Group; EGFR = epidermal growth factor receptor; EGFR-TKI = epidermal growth factor receptor tyrosine kinase inhibitor; HBV = hepatitis B virus; HBsAg = hepatitis B surface antigen; HCV = hepatitis C virus; HGB = hemoglobin; HIV = human immunodeficiency virus; INR = international normalized ratio; MCH = mean corpuscular hemoglobin; MCHC = mean corpuscular hemoglobin concentration; MCV = mean corpuscular volume; MRI = magnetic resonance imaging; MUGA = multiple-gated acquisition scan; PK = pharmacokinetic; PR = partial response; PT = prothrombin time; RECIST = Response Evaluation Criteria in Solid Tumors; SAE= serious adverse event; PI3Kα = phosphatidylinositol kinase α; WBC=white blood cell.

Notes:

1. The single dose phase lasts 7 days. The necessity of a single dose phase or the length of the single dose phase for subsequent subjects will be determined by based on the PK data observed in the first 3 dose groups; In the continuous dose phase of the study, one cycle is 28 days.
2. The tumor is evaluated according to RECIST (Version 1.1). CT or MRI can be used for tumor imaging at the investigator's discretion. However, the evaluation method, machines and technical parameters used should remain consistent throughout the study; Contrast agents should be used if not contraindicated. Imaging results will be interpreted by site investigators or radiologists. Reader should not be changed throughout the study. If tumor evaluation has been performed within **28** days

prior to the first dose using the same method and machine in the same hospital, the results can be used as baseline tumor evaluation. Baseline tumor assessment should include thorax, abdomen, pelvis and any other suspected sites of tumor lesions. For patients with bone metastasis, bone scan should be performed to follow lesion. Imaging examination will be performed for tumor assessment every 6 weeks (±7 days) from Day 1 of continuous dose phase until disease progression, unacceptable toxicity, withdrawal of consent or death. The investigator may schedule additional imaging examinations based on the patient's clinical condition. Confirmatory assessment must be completed within 6 weeks (42 days ± 7 days) after efficacy is evaluated as complete response (CR) or PR (per RECIST Version 1.1).If the patient discontinues treatment due to AE or other reasons, tumor evaluation will still be performed as scheduled. If the patient withdraws from the study due to disease progression, it is not necessary to repeat imaging assessment at the last visit. For subjects with suspected disease progression before the next scheduled assessment, unscheduled tumor evaluation should be performed once. If a subject is withdrawn from the clinical study for non-PD reasons, the tumor assessment will not be performed if the last tumor assessment is not older than 28 days.

3. Physical examination: including height (only at screening), body weight, head, eyes, ears, nose, larynx, neck, heart, thorax (including lungs), abdomen, limbs, skin, lymph nodes, nervous system and the patient's general condition. In addition to screening phase and Day 1 of treatment phase, additional physical examination will be performed as clinically indicated in the continuous dose phase.
4. Vital signs: including blood pressure, pulse rate, respiratory rate and body temperature. Blood pressure measurement should be performed on the patient after 5 minutes of rest in a sitting position.
5. During the continuous dose phase, echocardiography or MUGA will be performed every 8 weeks (±7 days) or as clinically indicated. This test can be exempted in the screening phase if echocardiography or MUGA has been performed no older than 28 days (including prior to the informed consent) prior to the first dose, using the same method and machine in the same hospital.
6. Complete blood cell count includes: RBC, HGB, MCH, MCHC, MCV, platelet count, WBC count and differential count, neutrophil count, lymphocyte count, monocyte count, basophil count and eosinophil count.
7. Serum virology, including HBsAg, anti-HCV antibody, HIV, HBV DNA and HCV RNA. Hepatitis B surface antigen and anti-HCV antibody tests should be performed. HBV DNA test should be performed if HBsAg test is positive; in addition, HBV DNA test is required every 3 treatment cycles to guide appropriate anti-HBV drug therapy. If anti-HCV antibody test is positive, HCV RNA test should be performed. This test can be exempted if a virological test report (with the normal reference intervals) issued by a formal medical institution within 28 days prior to the first dose (including prior to the signing of the ICF) is available.
8. Coagulation test, including PT, APTT and INR, is required at both the screening visit and the end of treatment visit; If the screening results are not clinically significant, the test does not need to be repeated on the day of the first dose and can be performed thereafter as clinically indicated.
9. Urinalysis items include specific gravity, pH value, urine glucose, urine protein, urine cast, urine creatinine, acetone body and blood cells. If the results of the above tests within 7 days prior to Day 1 are available, it is not necessary to repeat prior to the first dose on Day 1. If quantitative urine protein is ≥ 2+, quantitative 24-hour urine protein test will be performed.
10. Concomitant medication/treatment should be documented from the date of the ICF being signed during the screening phase to 30 days after the last dose of the study drug.
11. AE/SAE should be documented from the date of the ICF being signed during the screening phase to 30 days the last dose of the study drug.
12. PK blood collection should be performed with reference to Appendix 2 PK blood sample collection schedule. Time points of PK blood collection prior to dosing: fasting for 10 h is required before blood collection, and water consumption is not prohibited; Continuous PK blood sampling points: (Day 1 of single dose phase, C1D28 of continuous dose phase), food should not be consumed within 4 h, and water should not be consumed for at least 1 h after administration. The blood collection time points may be adjusted subsequently based on modifications made in the single dose phase.
13. If agreed, patients can provide tumor tissue sections (unstained, approximately -4 - 30 sections) prior to enrollment; or/and formalin-fixed paraffin-embedded histopathological tumor tissue blocks (200 mm^3) and/or fresh tumor samples (1 or 2 punch biopsy tissues 20 - 150 mg), which will be sent to the designated central

laboratory for the determination of tumor biomarkers for response evaluation. The test items are determined according to the type of specimen collected and the quantity of accepted specimens. If an amount of tumor tissue samples satisfying the requirement of tumor biomarker exploration in this study has been collected, the collection can be stopped.

14. These tests can be exempted if the early withdrawal/end of treatment visit is within 14 days after the last treatment visit.
15. This test may be waived on D1 if the screening results are not older than 14 days prior to D1.
16. If the results of the screening phase are within 7 days of D1, this test may be exempted on D1. The test has been performed on the day before the first dose and does not need to be performed on that day. If the screening results are not older than 7 days prior to D1, this test can be waived on D1, and does not need to be repeated if the patient underwent the test one day prior to the first dose.
17. Blood glucose is measured on Day 1 of the single dose phase (pre-dose, and 1 h and 24 h post-dose, respectively); as well as pre-dose on Days 1, 2, 8, 15 and 22 of Cycle of the continuous dose phase; To be performed pre-dose on Days 1, 8, 15, and 22 of Cycle 2, and pre-dose on Day 1 of each subsequent treatment cycle. For measurement of fasting blood glucose, 10 h of fasting is required. On dates of blood biochemistry, blood glucose may be included, without needing additional blood draw; On dates without blood biochemistry (pre-dose on C1D2, C2D8 and C2D22, etc.), blood may be collected from fingertips for testing if venous blood is not collected. The blood collection time points may be adjusted subsequently based on modifications made in the single dose phase. For DLT assessment or if necessary, fasting blood glucose test should be repeated within 24 h. Unscheduled visits may be added if necessary.
18. Blood sampling for pharmacodynamic biomarkers, including at baseline, should be performed before administration for patients with single dose phase (within 2 h), otherwise 8 ml of peripheral venous blood should be collected before CID1 administration (within 2 h), 4 h after C2D8 administration (± 30 min), and at CR, PR and PD, and sent to the designated central laboratory to determine biomarkers related to the treatment response. Blood samples for CR, PR, and PD should be collected no older than 7 days prior to that CR, PR or PD evaluation. Blood samples for PD biomarker evaluation will be collected at the dose level jointly determined by the investigator and the sponsor in light of the safety, efficacy and PK data, and blood collection will be stopped once the collected blood samples for PD biomarker evaluation have met the requirement of PD investigation in this study.
19. Complete blood biochemistry includes blood urea nitrogen/urea, creatinine, sodium, potassium, magnesium, chlorine, bicarbonate, calcium, phosphorus, blood sugar, total bilirubin, direct bilirubin, ALT, AST, ALP, LDH, total cholesterol, total protein, and albumin. Ten (10) h of fasting is required for blood biochemistry. This test will be performed on CXD1 and CXD15 of each treatment cycle, respectively from Cycle 2, and then after 4 cycles, may be performed on CXD1 of each cycle as clinically indicated, until completion of the early termination/end of treatment visit.

Appendix 1.2 List of Phase Ia Assessments (Continuous Dose Phase)

	Molecular Screening phase[20]	Clinical screening phase D-28-D-1	Continuous dose phase[1]												Early termination/end of treatment Within ≤ 7 days after the last dose of the study drug (± 3 days)	Safety Follow-up 30 days after the last dose of study drug (± 7 days)
			C1						C2				C3 and subsequent cycles			
			C1D1 (± 2 days)	C1D2	C1D8 (± 2 days)	C1D15 (± 2 days)	C1D22 (± 2 days)	C1D28 (± 2 days)	C2D1 (± 3 days)	C2D8	C2D15 (± 3 days)	C2D22 (± 3 days)	CXD1 (± 3 days)	CXD15 (± 3 days)		
ICF for molecular screening	X															
PIK3CA genetic diagnosis report [21]	X															
Collection of tumor tissues for molecular screening [21]	X															
Tumor pathological diagnosis report [21]	X															
ICF for clinical screening		X														
Demographics	X	X														
Inclusion/exclusion criteria evaluation		X														
Past medical history		X														
Tumor history	X	X														

	Molecular Screening phase[20]	Clinical screening phase D-28-D-1	Continuous dose phase[1]												Early termination/end of treatment Within ≤ 7 days after the last dose of the study drug (± 3 days)	Safety Follow-up 30 days after the last dose of study drug (± 7 days)
			C1						C2				C3 and subsequent cycles			
			C1D1 (± 2 days)	C1D2	C1D8 (± 2 days)	C1D15 (± 2 days)	C1D22 (± 2 days)	C1D28 (± 2 days)	C2D1 (± 3 days)	C2D8	C2D15 (± 3 days)	C2D22 (± 3 days)	CXD1 (± 3 days)	CXD15 (± 3 days)		
Serum β HCG test for women of childbearing age		X	X[15]						X				X		X[14]	
Tumor assessment (RECIST Version 1.1)[2]		X	Every 6 weeks ± 7 days from Day 1 of the continuous dose phase													
Complete physical examination[3]		X	X						X				X		X[14]	X
Vital signs and ECOG score [4]		X	X		X	X	X		X				X		X[14]	X
Echocardiography or MUGA[5]		X	Every 8 weeks ± 7 days from Day 1 of continuous dose phase [15]													
12-lead electrocardiogram		X	X		X	X	X		X				X		X[14]	X
Hematology[6]		X	X[16]		X	X	X		X				X		X[14]	X
Blood biochemistry[19]		X	X[16]		X	X	X		X		X		X	X	X[14]	X
HbA1c		X														
Blood glucose [17]		X	X	X	X	X	X		X	X	X	X	X		X	X
Serum virology[7]		X														

	Molecular Screening phase[20]	Clinical screening phase D-28-D-1	Continuous dose phase[1]												Early termination/end of treatment Within ≤ 7 days after the last dose of the study drug (± 3 days)	Safety Follow-up 30 days after the last dose of study drug (± 7 days)
			C1						C2				C3 and subsequent cycles			
			C1D1 (± 2 days)	C1D2	C1D8 (± 2 days)	C1D15 (± 2 days)	C1D22 (± 2 days)	C1D28 (± 2 days)	C2D1 (± 3 days)	C2D8	C2D15 (± 3 days)	C2D22 (± 3 days)	CXD1 (± 3 days)	CXD15 (± 3 days)		
Coagulation test (PT, APTT, INR) [8]		X	X[16]												X[14]	X
Serum amylase and lipase (LPS)		X	X[16]		X	X	X		X				X		X[14]	X
Urinalysis[9]		X	X[16]		X	X	X		X				X		X[14]	X
Concomitant medication/treatment[10]		X	X													X
AE and SAE evaluation[11]		X	X													X
DLT evaluation			X													
PK blood collection[12]			X		X	X		X								
Tumor tissue samples[13]		X														
Blood collection for PD biomarker exploration [18]			X							X						
Dispensing and returning of study drug			X						X				X		X	

Abbreviations: β-hCG = beta-human chorionic gonadotropin; AE = adverse event; ALT = alanine aminotransferase; APTT = activated partial thromboplastin time; AST = aspartate aminotransferase; CR = complete response; CT = computed tomography; CXDX = Day X of Cycle X; DLT = dose-limiting toxicity; ECOG = Eastern Cooperative Oncology Group; EGFR = epidermal growth factor receptor; EGFR-TKI = epidermal growth factor receptor tyrosine kinase inhibitor; HBV = hepatitis B virus; HBsAg =

hepatitis B surface antigen; HCV = hepatitis C virus; HGB = hemoglobin; HIV = human immunodeficiency virus; INR = international normalized ratio; MCH = mean corpuscular hemoglobin; MCHC = mean corpuscular hemoglobin concentration; MCV = mean corpuscular volume; MRI = magnetic resonance imaging; MUGA = multiple-gated acquisition scan; PK = pharmacokinetic; PR = partial response; PT = prothrombin time; RECIST = Response Evaluation Criteria in Solid Tumors; SAE= serious adverse event; PI3Kα = phosphatidylinositol kinase α; WBC=white blood cell.

Notes:

1. In the continuous dose phase of the study, one cycle is 28 days.
2. The tumor is evaluated according to RECIST (Version 1.1). CT or MRI can be used for tumor imaging at the investigator's discretion. However, the evaluation method, machines and technical parameters used should remain consistent throughout the study; Contrast agents should be used if not contraindicated. Imaging results will be interpreted by site investigators or radiologists. Reader should not be changed throughout the study. If tumor evaluation has been performed within **28** days prior to the first dose using the same method and machine in the same hospital, the results can be used as baseline tumor evaluation. Baseline tumor assessment should include thorax, abdomen, pelvis and any other suspected sites of tumor lesions. For patients with bone metastasis, bone scan should be performed to follow lesion. Imaging examination will be performed for tumor assessment every 6 weeks (±7 days) from Day 1 of continuous dose phase until disease progression, unacceptable toxicity, withdrawal of consent or death. The investigator may schedule additional imaging examinations based on the patient's clinical condition. Confirmatory assessment must be completed within 6 weeks (42 days ± 7 days) after efficacy is evaluated as complete response (CR) or PR (per RECIST Version 1.1).If the patient discontinues treatment due to AE or other reasons, tumor evaluation will still be performed as scheduled. If the patient withdraws from the study due to disease progression, it is not necessary to repeat imaging assessment at the last visit. For subjects with suspected disease progression before the next scheduled assessment, unscheduled tumor evaluation should be performed once. If a subject is withdrawn from the clinical study for non-PD reasons, the tumor assessment will not be performed if the last tumor assessment is not older than 28 days.
3. Physical examination: including height (only at screening), body weight, head, eyes, ears, nose, larynx, neck, heart, thorax (including lungs), abdomen, limbs, skin, lymph nodes, nervous system and the patient's general condition. In addition to screening phase and Day 1 of treatment phase, additional physical examination will be performed as clinically indicated in the continuous dose phase.
4. Vital signs: including blood pressure, pulse rate, respiratory rate and body temperature. Blood pressure measurement should be performed on the patient after 5 minutes of rest in a sitting position.
5. During the continuous dose phase, echocardiography or MUGA will be performed every 8 weeks (±7 days) or as clinically indicated. This test can be exempted in the screening phase if echocardiography or MUGA has been performed no older than 28 days (including prior to the informed consent) prior to the first dose, using the same method and machine in the same hospital.
6. Complete blood cell count includes: RBC, HGB, MCH, MCHC, MCV, platelet count, WBC count and differential count, neutrophil count, lymphocyte count, monocyte count, basophil count and eosinophil count.
7. Serum virology, including HBsAg, anti-HCV antibody, HIV, HBV DNA and HCV RNA. Hepatitis B surface antigen and anti-HCV antibody tests should be performed. HBV DNA test should be performed if HBsAg test is positive; in addition, HBV DNA test is required every 3 treatment cycles to guide appropriate anti-HBV drug therapy. If anti-HCV antibody test is positive, HCV RNA test should be performed. This test can be exempted if a virological test report (with the normal reference intervals) issued by a formal medical institution within 28 days prior to the first dose (including prior to the signing of the ICF) is available.
8. Coagulation test, including PT, APTT and INR, is required at both the screening visit and the end of treatment visit; If the screening results are not clinically significant, the test does not need to be repeated on the day of the first dose and can be performed thereafter as clinically indicated.
9. Urinalysis items include specific gravity, pH value, urine glucose, urine protein, urine cast, urine creatinine, acetone body and blood cells. If the results of the above tests within 7 days prior to Day 1 are available, it is not necessary to repeat prior to the first dose on Day 1. If quantitative urine protein is ≥ 2+, quantitative 24-hour urine protein test will be performed.

10. Concomitant medication/treatment should be documented from the date of the ICF being signed during the screening phase to 30 days after the last dose of the study drug.
11. AE/SAE should be documented from the date of the ICF being signed during the screening phase to 30 days the last dose of the study drug.
12. PK blood collection should be performed with reference to Appendix 2 PK blood sample collection schedule. Time points of PK blood collection prior to dosing: fasting for 10 h is required before blood collection, and water consumption is not prohibited; Continuous PK blood sampling points: (C1D1 and C1D28 of continuous dose phase), food should not be consumed within 4 h, and water should not be consumed for at least 1 h after administration. The blood collection time points may be adjusted subsequently based on modifications made in the single dose phase. The PK study will be carried out in the Phase Ia dose escalation part, and will be conducted at selected dose levels in some subjects in the Phase Ia dose expansion part.
13. If agreed, patients can provide tumor tissue sections (unstained, approximately -4 - 30 sections) prior to enrollment; or/and formalin-fixed paraffin-embedded histopathological tumor tissue blocks (200 mm^3) and/or fresh tumor samples (1 or 2 punch biopsy tissues 20 - 150 mg), which will be sent to the designated central laboratory for the determination of tumor biomarkers for response evaluation. The test items are determined according to the type of specimen collected and the quantity of accepted specimens. If an amount of tumor tissue samples satisfying the requirement of tumor biomarker exploration in this study has been collected, the collection can be stopped.
14. These tests can be exempted if the early withdrawal/end of treatment visit is within 14 days after the last treatment visit.
15. This test may be waived on D1 if the screening results are not older than 14 days prior to D1.
16. If the results of the screening phase are within 7 days of D1, this test may be exempted on D1. The test has been performed on the day before the first dose and does not need to be performed on that day. If the screening results are not older than 7 days prior to D1, this test can be waived on D1, and does not need to be repeated if the patient underwent the test one day prior to the first dose.
17. Blood glucose is measured on Day 1 of the single dose phase (pre-dose, and 1 h and 24 h post-dose, respectively); as well as pre-dose on Days 1, 2, 8, 15 and 22 of Cycle of the continuous dose phase; To be performed pre-dose on Days 1, 8, 15, and 22 of Cycle 2, and pre-dose on Day 1 of each subsequent treatment cycle. For measurement of fasting blood glucose, 10 h of fasting is required. On dates of blood biochemistry, blood glucose may be included, without needing additional blood draw; On dates without blood biochemistry (pre-dose on C1D2, C2D8 and C2D22, etc.), blood may be collected from fingertips for testing if venous blood is not collected. The blood collection time points may be adjusted subsequently based on modifications made in the single dose phase. For DLT assessment or if necessary, fasting blood glucose test should be repeated within 24 h. Unscheduled visits may be added if necessary.
18. Blood sampling for pharmacodynamic biomarkers includes baseline blood collection before CID1 administration (within 2 h), 8 ml of peripheral venous blood 4 h after C2D8 administration (± 30 min), and at CR, PR and PD, and sent to the designated central laboratory to determine biomarkers related to the treatment response. Blood samples for CR, PR, and PD should be collected no older than 7 days prior to that CR, PR or PD evaluation. Blood samples for PD biomarker evaluation will be collected at the dose level jointly determined by the investigator and the sponsor in light of the safety, efficacy and PK data, and blood collection will be stopped once the collected blood samples for PD biomarker evaluation have met the requirement of PD investigation in this study.
19. Complete blood biochemistry includes blood urea nitrogen/urea, creatinine, sodium, potassium, magnesium, chlorine, bicarbonate, calcium, phosphorus, blood sugar, total bilirubin, direct bilirubin, ALT, AST, ALP, LDH, total cholesterol, total protein, and albumin. Ten (10) hours of fasting is required for blood biochemistry. This test will be performed on CXD1 and CXD15 of each treatment cycle, respectively from Cycle 2, and then after 4 cycles, may be performed on CXD1 of each cycle as clinically indicated, until completion of the early termination/end of treatment visit.
20. Inclusion criteria for molecular screening are applicable to patients with PIK3CA gene mutation included in Phase Ia dose expansion part. Molecular screening phase refers to the phase before clinical screening.
21. Evidence of abnormal PIK3CA gene mutation can be obtained from pathology and genetic diagnosis reports of the patient's tumor tissue provided by medical/diagnostic facilities. Patients may enter the clinical screening and subsequent study phases, while tumor tissue specimens (at least 10 unstained tumor

tissue sections; or/and formalin-fixed paraffin-embedded pathological tumor tissue or/and fresh tumor tissue) and copies of the corresponding pathology reports and genetic diagnosis reports to the designated screening laboratory for confirmation. If there is no medical/diagnostic report supporting that the patient has PIK3CA gene mutations, tumor tissue specimens (at least 10 unstained tumor tissue sections; or/and formalin-fixed paraffin-embedded pathological tumor tissue or/and fresh tumor tissue) and copies of the corresponding pathology reports and genetic diagnosis reports must be sent to the designated screening laboratory for molecular screening. Each site can conduct the clinical screening of patients only when the PIK3CA genetic test result is available.

Appendix 2 PK Blood Sample Collection Schedule for Phase Ia

Dosing phase: single dose phase and continuous dose phase

Scheduled study visits[1]	Blood collection time points (hours)[2]	Time window (minute)
Day 1 of the single dose phase	Pre-dose	15
	0.5	5
	1	5
	2	10
	4	10
	8	10
	12	15
Day 2 of the single dose phase	24	30
Day 2 of the single dose phase	36	30
Day 3 of the single dose phase	48	30
Day 3 of the single dose phase	72	30
C1D1	Pre-dose	15
C1D8	Pre-dose	15
C1D15	Pre-dose	15
C1D28	Pre-dose	15
	0.5	5
	1	5
	2	10
	3	10
	4	10
	6	10
	8	10
	12	15
	24[4]	15

Administration Phase: Continuous Dose Phase

Scheduled study visits[1]	Blood collection time points (hours)[2]	Time window (minute)
C1D1	Pre-dose	15
	0.5	5
	1	5
	2	10
	3	10
	4	10
	6	10
	8	10
	12	15
	24[4]	15
C1D8	Pre-dose	15
C1D15	Pre-dose	15
C1D28	Pre-dose	15
	0.5	5
	1	5
	2	10
	3	10
	4	10
	6	10

	8	10
	12	15
	24[4]	15

Abbreviations: CRF = Case Report Form;
Notes:
1. The investigator must accurately record the blood collection time and the patient's dosing time in the CRF. In the event of overdose, hepatotoxicity (if applicable) or optometric event (if applicable), 2 mL of whole blood will be additionally collected to determine the concentration of study drug.
2. 2 mL whole blood will be collected each time.
3. Time points of PK blood collection prior to dosing: fasting for 10 h is required before blood collection, and water consumption is not prohibited; Continuous PK blood sampling points (Day 1 of single dose phase, C1D1 (if any), C1D28 of continuous dose phase), food should not be consumed within 4 h, and water should not be consumed for at least 1 h after administration.
4. The 24-h blood sample on C1D1 (if available) and C1D28 will be collected within 15 min before C1D2 and C2D1 administration, respectively.
5. The PK study will be carried out in the Phase Ia dose escalation part, and will be conducted at selected dose levels in some subjects in the Phase Ia dose expansion part.

[redacted]

	[redacted]	[redacted]	[redacted]				[redacted]	[redacted]	[redacted]
			[redacted]	[redacted]	[redacted]	[redacted]			
[redacted]	[redacted]								
[redacted]	[redacted]								
[redacted]	[redacted]								
[redacted]	[redacted]								
[redacted]		[redacted]							
[redacted]	[redacted]	[redacted]							
[redacted]		[redacted]							
[redacted]		[redacted]							
[redacted]	[redacted]	[redacted]							
[redacted]		[redacted]	[redacted]		[redacted]	[redacted]	[redacted]		
[redacted]		[redacted]	[redacted]						

	[redacted]	[redacted]	[redacted]				[redacted]	[redacted]	[redacted]
			[redacted]	[redacted]	[redacted]	[redacted]			
[redacted]		[redacted]	[redacted]		[redacted]	[redacted]	[redacted]	[redacted]	
[redacted]		[redacted]	[redacted]		[redacted]	[redacted]	[redacted]	[redacted]	
[redacted]		[redacted]	[redacted]						
[redacted]		[redacted]	[redacted]		[redacted]	[redacted]	[redacted]	[redacted]	
[redacted]		[redacted]	[redacted]		[redacted]	[redacted]	[redacted]	[redacted]	
[redacted]		[redacted]	[redacted]		[redacted]	[redacted]	[redacted]	[redacted]	
[redacted]		[redacted]							
[redacted]		[redacted]	[redacted]	[redacted]	[redacted]	[redacted]	[redacted]	[redacted]	
[redacted]		[redacted]							
[redacted]		[redacted]	[redacted]				[redacted]	[redacted]	
[redacted]		[redacted]	[redacted]		[redacted]	[redacted]	[redacted]	[redacted]	
[redacted]		[redacted]	[redacted]		[redacted]	[redacted]	[redacted]	[redacted]	
[redacted]		[redacted]	[redacted]						
[redacted]		[redacted]	[redacted]						

	[redacted]	[redacted]	[redacted]				[redacted]	[redacted]	[redacted]
			[redacted]	[redacted]	[redacted]	[redacted]			
[redacted]		[redacted]							
[redacted]			[redacted]	[redacted]					
[redacted]			[redacted]		[redacted]	[redacted]	[redacted]		
[redacted]								[redacted]	[redacted]

[redacted]

[redacted]

[redacted]

[redacted]

Appendix 4 Estimation of Blood Volume to Be Collected (Phase Ia/Ib)

**Time point**	**Assessment parameters**	**Total blood volume (mL)**
**Phase Ia**		
Screening phase	Serum Pregnancy Test (if appropriate)	2
	HBV, HCV, HIV, HBV DNA and HCV RNA tests [1]	2-4
	Coagulation testing	2
	Blood chemistry	5
	Hematology panel	2.5
	HbA1c	2
	Serum amylase	2
	Serum lipase	2
	Total	19.5 or 21.5
**Single dose phase**		
D1	Coagulation testing	2
	Blood chemistry	5
	Serum amylase	2
	Serum lipase	2
	Hematology panel	2.5
	PK[2]	2 × 7= 14
	Blood collection for PD biomarker exploration [4]	8
	Total	35.5
D2	PK[2]	2 × 2= 4
	Total	4
D3	PK[2]	2 × 2= 4
	Total	4
**Continuous dose phase**		
C1D1	Serum Pregnancy Test (if appropriate)	2
	Blood chemistry	5
	Serum amylase	2
	Serum lipase	2
	Hematology panel	2.5
	Coagulation testing	2
	PK[2] (if there is a single dose phase)	2
	PK[2]	20
	Blood collection for PD biomarker exploration [4]	8
	Total	25.5 or 43.5
C1D8	Blood chemistry	5
	Serum amylase	2
	Serum lipase	2
	Hematology panel	2.5
	Coagulation test (if appropriate)	2
	PK[2]	2

	Total	15.5
C1D15	Blood chemistry	5
	Serum amylase	2
	Serum lipase	2
	Hematology panel	2.5
	Coagulation test (if appropriate)	2
	PK[2]	2
	Total	15.5

C1D22	Blood chemistry	5
	Serum amylase	2
	Serum lipase	2
	Hematology panel	2.5
	Coagulation test (if appropriate)	2
	Total	13.5
C1D28	PK[2]	2× 10
	Total	20
C2D1	Serum Pregnancy Test (if appropriate)	2
	Blood chemistry	5
	Serum amylase	2
	Serum lipase	2
	Hematology panel	2.5
	Coagulation test (if appropriate)	2
	Total	15.5
C2D8	Blood collection for PD biomarker investigation	8
	Total	8
C2D15	Biochemistry test	5
	Total	5
CXD1	Serum Pregnancy Test (if appropriate)	2
	Blood chemistry	5
	Serum amylase	2
	Serum lipase	2
	Hematology panel	2.5
	Coagulation test (if appropriate)	2
	Total	15.5
CXD15	Biochemistry test	5
	Total	5
Complete response	Blood collection for PD biomarker investigation	8
Partial response	Blood collection for PD biomarker investigation	8
Neoplasm progression	Blood collection for PD biomarker investigation	8
	Total	24
End-of-treatment visit[3]	Serum Pregnancy Test (if appropriate)	2

	Blood chemistry	5
	Serum amylase	2
	Serum lipase	2
	Hematology panel	2.5
	Coagulation testing	2
	Total	15.5
Safety follow-up visits	Blood chemistry	5
	Serum amylase	2
	Serum lipase	2
	Hematology panel	2.5
	Coagulation testing	2
	Total	13.5
[redacted]		
[redacted]	[redacted]	[redacted]
	[redacted]	[redacted]
	[redacted]	[redacted]
	[redacted]	[redacted]
	[redacted]	[redacted]
	[redacted]	[redacted]
	[redacted]	[redacted]
	[redacted]	[redacted]
	[redacted]	[redacted]
[redacted]	[redacted]	[redacted]
	[redacted]	[redacted]
	[redacted]	[redacted]
	[redacted]	[redacted]
	[redacted]	[redacted]
	[redacted]	[redacted]
	[redacted]	[redacted]
	[redacted]	[redacted]
[redacted]	[redacted]	[redacted]
	[redacted]	[redacted]
[redacted]	[redacted]	[redacted]
	[redacted]	[redacted]
	[redacted]	[redacted]
	[redacted]	[redacted]
	[redacted]	[redacted]
	[redacted]	[redacted]
	[redacted]	[redacted]
[redacted]	[redacted]	[redacted]
[redacted]	[redacted]	[redacted]

[redacted]	[redacted]	[redacted]
	[redacted]	[redacted]
[redacted]	[redacted]	[redacted]
	[redacted]	[redacted]
	[redacted]	[redacted]
	[redacted]	[redacted]
	[redacted]	[redacted]
	[redacted]	[redacted]
	[redacted]	[redacted]
[redacted]	[redacted]	[redacted]
	[redacted]	[redacted]
	[redacted]	[redacted]
	[redacted]	[redacted]
	[redacted]	[redacted]
	[redacted]	[redacted]

Abbreviations: CXD1 = Cycle X Day 1; CRF = Case Report Form; HBV = Hepatitis B Virus; HCV = Hepatitis C Virus; HIV = human immunodeficiency virus; PK = pharmacokinetics

Notes:

1. HBV, HCV and HIV tests will be performed according to local clinical practice. HBV DNA test should be performed if HBsAg test is positive. If anti-HCV antibody test is positive, HCV RNA test should be performed.
2. For PK blood sample collection schedule, see sections Pharmacokinetic Assessment and Appendix 2.The investigator must accurately record the blood collection time and the patient's dosing time in the CRF.
3. Patients withdrawing from this study prematurely are required to provide blood samples before withdrawal for blood biochemistry, hematology and coagulation test.
4. Phase Ia baseline blood sampling for pharmacodynamic biomarkers: For patients with single dose phase, blood should be collected before single dose, otherwise it should be performed before CID1 administration.

Appendix 5 Eastern Cooperative Oncology Group (ECOG) Performance Status

**Grade**	**ECOG**
0	Fully active, able to perform all normal activity without restriction
1	Restricted in physically strenuous activity but ambulatory and able to carry out work of a light or sedentary nature, e.g., light house work, office work
2	Ambulatory and capable of all self-care but unable to carry out any work activities. Up and about more than 50% of waking hours
3	Capable of only limited self-care, confined to bed or chair more than 50% of waking hours
4	Completely disabled; cannot carry on any self-care; totally confined to bed or chair
5	Death

Appendix 6 Response Evaluation Criteria in Solid Tumors (RECIST1.1)

Response Evaluation Criteria in Solid Tumors Version 1.1

(Response Evaluation Criteria in Solid Tumors RECIST Version 1.1)

Since there is no publication of official Chinese version of RECIST 1.1 available at present, this internal translated Chinese version is only for reference. For more detailed information, please refer to the original English Version at http://ctep.cancer.gov/protocolDevelopment/docs/recist_guideline.pdf.

Abstract

Background Introduction

Assessment of the change in tumor burden is an important feature of the clinical evaluation of cancer therapeutics. Both tumor shrinkage (objective response) and disease progression are useful endpoints in clinical trials. Since RECIST was published in 2000, many investigators, cooperative groups, industry and government authorities have adopted these criteria in the assessment of treatment outcomes. However, a number of questions and issues have arisen which have led to the development of a revised RECIST (Version 1.1). Evidence for changes, summarized in separate papers in this special issue, has come from assessment of a large data warehouse (> 6500 patients), simulation studies and literature reviews.

Highlights of Revised RECIST 1.1

Major changes include:

Number of lesions to be assessed: based on evidence from numerous trial databases merged into a data warehouse for analysis purposes, the number of lesions required to assess tumor burden for response determination has been reduced from a maximum of 10 to a maximum of five in total (and from five to two per organ, maximally).

Assessment of pathological lymph nodes is now incorporated: nodes with a short axis of ≥ 15mm are considered measurable and assessable as target lesions. The short axis measurement should be included in the sum of lesions in calculation of tumor response. Nodes that shrink to < 10 mm short axis are considered normal.

Confirmation of response is required for trials with response as primary endpoint but is no longer required in randomized, controlled studies since the control arm serves as an appropriate means of interpretation of data. Disease progression is clarified in several aspects: in addition to the previous definition of progression in target disease (radius) of 20% increase in sum, a 5 mm absolute increase is now required as well to guard against over calling PD when the total sum is very small. Furthermore, there is guidance offered on what constitutes ‘unequivocal progression’ of non-measurable/non-target disease, a source of confusion in the original RECIST guideline. Finally, a section on detection of new lesions, including the interpretation of FDG-PET scan assessment is included. Imaging guidance: the revised RECIST includes a new imaging appendix with updated recommendations on the optimal anatomical assessment of lesions.

Future work:

A key question considered by the RECIST Working Group in developing RECIST 1.1 was whether it was appropriate to move from anatomic unidimensional assessment of tumor burden to either volumetric anatomical assessment or to functional assessment with PET or MRI. It was concluded that, at present, there is not sufficient standardization or evidence to abandon anatomical assessment of tumor burden. The only exception to this is in the use of FDG-PET imaging as an adjunct to determination of progression. As is detailed in the final paper in this special issue, the use of these promising newer approaches requires appropriate clinical validation studies.

Key words: Response evaluation criteria; Solid tumors; Guidance

1. Background

1.1. History of RECIST Criteria

Assessment of the change in tumor burden is an important feature of the clinical evaluation of cancer therapeutics. Both tumor shrinkage (objective response) and time to the development of disease progression are important endpoints in cancer clinical trials. The use of tumor regression as the endpoint for phase II trials screening new agents for evidence of anti-tumor effect is supported by years of evidence. suggesting that, for many solid tumors, agents which produce tumor shrinkage in a proportion of patients have a reasonable (albeit imperfect) chance of subsequently demonstrating an improvement in overall survival or other time to event measures in randomized phase III studies. At the current time objective response carries with it a body of evidence greater than for any other biomarker supporting its utility as a measure of promising treatment effect in phase II screening trials. Furthermore, in both phase II and phase III clinical trials of drug development, clinical trials in advanced disease settings are increasingly utilizing time to progression (or PFS) as an endpoint upon which efficacy conclusions are drawn, which is also based on anatomical measurement of tumor size.

However, both of these tumor endpoints, objective response and time to disease progression, are useful only if based on widely accepted and readily applied standard criteria based on anatomical tumor burden. In 1981, World Health Organization (WHO) first published tumor response criteria, mainly for use in trials where tumor response is the primary endpoint. The WHO criteria introduced the concept of an overall assessment of tumor burden by summing the products of bidimensional lesion measurements and determined response to therapy by evaluation of change from baseline while on treatment. However, in the decades that followed their publication, cooperative groups and pharmaceutical companies that used the WHO criteria often ‘modified’ them to accommodate new technologies or to address areas that were unclear in the original document. This led to confusion in interpretation of trial results. This led to confusion in interpretation of trial results and in fact, the application of varying response criteria was shown to lead to very different conclusions about the efficacy of the same regimen. In response to these problems, an International Working Party was formed in the mid-1990s to standardize and simplify response criteria.

New criteria, known as RECIST (Response Evaluation Criteria in Solid Tumors), were published in 2000. Key initial RECIST features include determination of the smallest measurable lesion size, and a description of the number of lesions followed (up to 10; maximum 5 per organ), use of one-dimensional rather than two-dimensional, overall evaluation of tumor burden. These criteria have subsequently been widely adopted by academic institutions, cooperative groups, and industry for trials where the primary

endpoints are objective response or progression. In addition, regulatory authorities accept RECIST as an appropriate guideline for these assessments.

2. Purpose of this Guidance:

This guidance describes a standard approach to solid tumor measurement and definitions for objective assessment of change in tumor size for use in adult and paediatric cancer clinical trials. It is expected these criteria will be useful in all trials where objective response is the primary study endpoint, as well as in trials where assessment of stable disease, tumor progression or time to progression analyses are undertaken, since all of these outcome measures are based on an assessment of anatomical tumor burden and its change on study. There are no assumptions in this paper about the proportion of patients meeting the criteria for any of these endpoints which will signal that an agent or treatment regimen is active: those definitions are dependent on type of cancer in which a trial is being undertaken and the specific agent(s) under study. Protocols must include appropriate statistical sections which define the efficacy parameters upon which the trial sample size and decision criteria are based. In addition to providing definitions and criteria for assessment of tumor response, this guideline also makes recommendations regarding standard reporting of the results of trials that utilize tumor response as an endpoint.

While these guidelines may be applied in malignant brain tumor studies, there are also separate criteria published for response assessment in that setting. This guideline is not intended for use for studies of malignant lymphoma since international guidelines for response assessment in lymphoma are published separately.

Finally, many oncologists in their daily clinical practice follow their patients' malignant disease by means of repeated imaging studies and make decisions about continued therapy on the basis of both objective and symptomatic criteria. It is not intended that these RECIST guidelines play a role in that decision making, except if determined appropriate by the treating oncologist.

3. Measurability of Tumor at Baseline

3.1 Definition

At baseline, tumor lesions/lymph nodes are categorized as measurable or non-measurable as follows:

3.1.1 Measurable Tumor Lesions: Must be accurately measured in at least one dimension (longest diameter in the plane of measurement is to be recorded) with a minimum size of:

- 10 mm by CT scan (CT scan slice thickness no greater than 5 mm).
- 10 mm caliper measurement by clinical examination (lesions which cannot be accurately measured with calipers should be recorded as non-measurable)
- 20 mm by chest X-ray.

Malignant Lymph Nodes: To be considered pathologically enlarged and measurable, a lymph node must be ≥ 15mm in short axis when assessed by CT scan (CT scan slice thickness recommended to be no greater than 5 mm). At baseline and in follow-up, only the short axis will be measured and followed. See also notes below on 'Baseline documentation of target and non-target lesions' for information on lymph node measurement.

3.1.2 Non-measurable (Tumors)

All other lesions, including small lesions (longest diameter <10mm or pathological lymph nodes with ≥10 to <15mm short axis) as well as truly non-measurable lesions. Lesions regarded as actually unmeasurable include: Meningeal disease, ascites, pleural or pericardial effusion, inflammatory breast disease, lymphangitic involvement of skin or lung, abdominal masses/abdominal organomegaly identified by pharmacological exam that is not measurable by reproducible imaging techniques.

3.1.3 Special Considerations Regarding Lesion Measurability

Bone lesions, cystic lesions, and lesions previously treated with local therapy require particular comment:

Bone lesions:

- Bone scan, PET scan or plain films are not considered adequate imaging techniques to measure bone lesions. However, these techniques can be used to confirm the presence or disappearance of bone lesions.
- Lytic bone lesions or mixed lytic-blastic lesions, with identifiable soft tissue components, that can be evaluated by cross sectional imaging techniques such as CT or MRI can be considered as measurable lesions if the soft tissue component meets the definition of measurability described above.
- Osteoblastic bone lesions are non-measurable.

Cystic lesions:

- Lesions that meet the criteria for radiographically defined simple cysts should not be considered as malignant lesions (neither measurable nor non-measurable) since they are, by definition, simple cysts.
- 'Cystic lesions' thought to represent cystic metastases can be considered as measurable lesions, if they meet the definition of measurability described above. However, if noncystic lesions are present in the same patient, these are preferred for selection as target lesions.

Lesions with prior local treatment:

- Tumor lesions situated in a previously irradiated area, or in an area subjected to other loco-regional therapy, are usually not considered measurable unless there has been demonstrated progression in the lesion. Study protocols should detail the conditions under which such lesions would be considered measurable.

3.2 Specifications by Methods of Measurements

3.2.1 Measurement of Lesions

All measurements should be recorded in metric notation, using calipers if clinically assessed. All baseline evaluations should be performed as close as possible to the treatment start and never more than 4 weeks before the beginning of the treatment.

3.2.2 Method of Measurement

The same method of assessment and the same technique should be used to characterize each reported lesion at baseline and during follow-up. Imaging based evaluation should always be done rather than clinical examination unless the lesion(s) being followed cannot be imaged.

Clinical lesions: Clinical lesions will only be considered measurable when they are superficial and ≥10mm diameter as assessed using calipers (e.g. skin nodules). For the case of skin lesions, documentation by color photography including a ruler to estimate the size of the lesion is suggested. As noted above, when lesions can be evaluated by both clinical

exam and imaging, imaging evaluation should be undertaken since it is more objective and may also be reviewed at the end of the study.

Chest X-ray: Chest CT is preferred over chest X-ray, particularly when progression is an important endpoint, since CT is more sensitive than X-ray, particularly in identifying new lesions. However, lesions on chest X-ray may be considered measurable if they are clearly defined and surrounded by aerated lung.

CT, MRI: CT is the best currently available and reproducible method to measure lesions selected for response assessment. This guideline has defined measurability of lesions on CT scan based on the assumption that CT slice thickness is 5mm or less. As is described in Appendix II, when CT scans have slice thickness greater than 5 mm, the minimum size for a measurable lesion should be twice the slice thickness. MRI is also acceptable in certain situations (e.g. for body scans). More details concerning the use of both CT and MRI for assessment of objective tumor response evaluation are provided in Appendix II.

Ultrasonography: ultrasound is not useful in assessment of lesion size and should not be used as a method of measurement. Ultrasound examinations cannot be reproduced in their entirety for independent review at a later date and, because they are operator dependent, it cannot be guaranteed that the same technique and measurements will be taken from one assessment to the next. If new lesions are identified by ultrasound in the course of the study, confirmation by CT or MRI is advised. If there is concern about radiation exposure at CT, MRI may be used instead of CT in selected instances.

Endoscopy, laparoscopy: The utilization of these techniques for objective tumor evaluation is not advised. However, they can be useful to confirm complete pathological response when biopsies are obtained or to determine relapse following complete response or surgical resection.

Tumor markers: Tumor markers alone cannot be used to assess objective tumor response. If markers are initially above the upper normal limit, however, they must normalize for a patient to be considered in complete response. Because tumor markers are disease specific, instructions for their measurement should be incorporated into protocols on a disease specific basis. Specific guidelines for both CA-125 response (in recurrent ovarian cancer) and PSA response (in recurrent prostate cancer), have been published. In addition, the Gynecologic Cancer Intergroup has developed CA125 progression criteria which are to be integrated with objective tumor assessment for use in first-line trials in ovarian cancer.

Cytology, histology: These techniques can be used to differentiate between PR and CR in rare cases if required by protocol (for example, residual lesions in tumor types such as germ cell tumors, where known residual benign tumors can remain). When effusions are known to be a potential adverse effect of treatment (e.g. with certain taxane compounds or angiogenesis inhibitors), the cytological confirmation of the neoplastic origin of any effusion that appears or worsens during treatment can be considered if the measurable tumor has met criteria for response or stable disease in order to differentiate between response (or stable disease) and progressive disease.

4. Tumor Response Evaluation

4.1 Assessment of Overall Tumor Burden and Measurable Disease

To assess objective response or future progression, it is necessary to estimate the overall tumor burden at baseline and use this as a comparator for subsequent measurements. Only patients with measurable disease at baseline should be included in protocols where objective tumor response is the primary endpoint. Measurable disease is defined by the presence of at least one measurable lesion. In studies where the primary endpoint is tumor progression (either time to progression or proportion with progression at a fixed date), the

protocol must specify if entry is restricted to those with measurable disease or whether patients having non-measurable disease only are also eligible.

4.2 Baseline Documentation of 'Target' and 'Non-target' Lesions

When more than one measurable lesion is present at baseline all lesions up to a maximum of five lesions total (and a maximum of two lesions per organ) representative of all involved organs should be identified as target lesions and will be recorded and measured at baseline (this means in instances where patients have only one or two organ sites involved a maximum of two and four lesions respectively will be recorded).

Target lesions must be selected on the basis of their size (lesions with the longest diameter), be representative of all involved organs, but in addition should be those that lend themselves to reproducible repeated measurements. It may be the case that, on occasion, the largest lesion does not lend itself to reproducible measurement, in which circumstance the next largest lesion which can be measured reproducibly should be selected.

Lymph nodes merit special mention since they are normal anatomical structures which may be visible by imaging even if not involved by tumor. Pathological nodes which are defined as measurable and may be identified as target lesions must meet the criterion of a short axis of ≥15mm by CT scan. Only the short axis of these nodes will contribute to the baseline sum. The short axis of the node is the diameter normally used by radiologists to judge if a node is involved by solid tumor. Nodal size is normally reported as two dimensions in the plane in which the image is obtained (for CT scan this is almost always the axial plane; for MRI the plane of acquisition may be axial, saggital or coronal). The smallest of these measures is the short axis. For example, an abdominal node which is reported as being 20 mm × 30 mm has a short axis of 20 mm and qualifies as a malignant, measurable node. In this example, 20 mm should be recorded as the node measurement. All other pathological nodes (those with short axis ≥ 10mm but < 15 mm) should be considered non-target lesions. Nodes that have a short axis < 10mm are considered non-pathological and should not be recorded or followed.

A sum of the diameters (longest for non-nodal lesions, short axis for nodal lesions) for all target lesions will be calculated and reported as the baseline sum diameters. If lymph nodes are to be included in the sum, then as noted above, only the short axis is added into the sum. The baseline sum diameters will be used as reference for baseline disease level.

All other lesions including pathological lymph nodes should be identified as non-target lesions and require no measurements but should also be recorded at baseline. Measurements are not required and these lesions should be followed as 'present', 'absent', or in rare cases 'unequivocal progression'. In addition, it is possible to record multiple target lesions involving the same organ as a single item (e.g. 'multiple enlarged pelvic lymph nodes' or 'multiple liver metastases').

4.3 Efficacy Assessment Criteria

This section provides the definitions of the criteria used to determine objective tumor response for target lesions.

4.3.1 Evaluation of Target Lesions

Complete Response (CR): Disappearance of all target lesions. Any pathological lymph nodes (whether target or non-target) must have short axis <10 mm.

Partial Response (PR): At least a 30% decrease in the sum of diameters of target lesions, taking as reference the baseline sum diameters.

Progressive Disease (PD): At least a 20% increase in the sum of diameters of target lesions, taking as reference the smallest sum on study (this includes the baseline sum if that is the smallest on study). In addition to the relative increase of 20%, the sum must also demonstrate an absolute increase of at least 5 mm. (Note: the appearance of one or more new lesions is also considered progression).

Stable Disease (SD): Neither lesion shrinkage is qualified with PR nor lesion expansion is qualified with PD, taking as reference the sum of diameters in smallest lesions during the study.

4.3.2 Special Notes on the Assessment of Target Lesions

Lymph nodes for Target Lesions:

Actual short axis measurement should be recorded (measured in the same anatomical plane as the baseline examination), even if the nodes regress to below 10mm on study. This means that when lymph nodes are included as target lesions, the 'sum' of lesions may not be zero even if complete response criteria are met, since a normal lymph node is defined as having a short axis of <10mm. Case report forms or other data collection methods may therefore be designed to have target nodal lesions recorded in a separate section where, in order to qualify for CR, each node must achieve a short axis <10mm. For PR, SD and PD, the actual short axis measurement of the nodes is to be included in the sum of target lesions.

Target lesions that become 'too small to measure':

While on study, all lesions (nodal and non-nodal) recorded at baseline should have their actual measurements recorded at each subsequent evaluation, even when very small (e.g. 2mm).

However, sometimes lesions or lymph nodes which are recorded as target lesions at baseline become so faint on CT scan that the radiologist may not feel comfortable assigning an exact measure and may report them as being 'too small to measure'. When this occurs, it is important that a value be recorded on the case report form. If it is the opinion of the radiologist that the lesion has likely disappeared, the measurement should be recorded as 0 mm. If the lesion is believed to be present and is faintly seen but too small to measure, a default value of 5mm should be assigned (Note: It is less likely that this rule will be used for lymph nodes since they usually have a definable size when normal and are frequently surrounded by fat such as in the retroperitoneum; However, if a lymph node is present but the signal is too weak to measure, the default value of 5 mm can also be recorded).

This default value is derived from the 5mm CT slice thickness (but should not be changed with varying CT slice thickness). The measurement of these lesions is potentially non-reproducible, therefore providing this default value will prevent false responses or progressions based upon measurement error. To reiterate, however, if the radiologist is able to provide an actual measure, that should be recorded, even if it is below 5mm.

Lesions that split or coalesce on treatment

As noted in Appendix II, when non-nodal lesions 'fragment', the longest diameters of the fragmented portions should be added together to calculate the target lesion sum. Similarly, as lesions coalesce, a plane between them may be maintained that would aid in obtaining

maximal diameter measurements of each individual lesion. If the lesions have truly coalesced such that they are no longer separable, the vector of the longest diameter in this instance should be the maximal longest diameter for the 'coalesced lesion'.

4.3.3 Assessment of Non-target Lesions

This section provides the definitions of the criteria used to determine the tumor response for the group of non-target lesions. While some non-target lesions may actually be measurable, they need not be measured and instead should be assessed only qualitatively at the time points specified in the protocol.

Complete response (CR): disappearance of all non-target lesions and normalization of tumor marker level. All lymph nodes must be non-pathological in size (< 10 mm short axis).

Non-CR/Non-PD: Persistence of one or more non-target lesion(s) and/or maintenance of tumor marker level above the normal limits.

Progressive disease (PD): Unequivocal progression of existing non-target lesions. (Note: appearance of one or more new lesions is also considered progression).

4.3.4 Special Notes on Assessment of Progression of Non-target Disease

The concept of progression of non-target disease requires additional explanation as follows: When the patient also has measurable disease, to achieve 'unequivocal progression' on the basis of the non-target disease, there must be an overall level of substantial worsening in non-target disease such that, even in presence of SD or PR in target disease, the overall tumor burden has increased sufficiently to merit discontinuation of therapy. A modest increase in the size of one or more non-target lesions is usually not sufficient to quality for unequivocal progression status. The designation of overall progression solely on the basis of change in non-target disease in the face of SD or PR of target disease will therefore be extremely rare.

When the patient has only non-measurable non-target disease: This circumstance arises in some phase III trials when it is not a criterion of study entry to have measurable disease. The same general concepts apply here as noted above, however, in this instance there is no measurable disease assessment. Because worsening in non-target disease cannot be easily quantified (by definition: if all lesions are truly non-measurable), a useful test that can be applied when assessing patients for unequivocal progression is to consider if the increase in overall disease burden based on the change in non-measurable disease is comparable in magnitude to the increase that would be required to declare PD for measurable disease : i.e., an increase in tumor burden representing an additional 73% increase in 'volume' (which is equivalent to a 20% increase diameter in a measurable lesion). Another example is peritoneal exudation from "trace" to "large"; lymphangiopathy from "local" to "extensive dissemination"; or described in the protocol as "sufficient to change the therapy". Some illustrative examples include pleural effusions from trace to large, lymphatic involvement spreading from the primary site to distant sites, or may be described in the protocol as "a change necessarily required in terms of treatment". If 'unequivocal progression' is seen, the patient should be considered to have had overall PD at that point. While it would be ideal to have objective criteria to apply to non-measurable disease, the very nature of that disease makes it impossible to do so, therefore the increase must be substantial.

4.3.5 New Lesions

The appearance of new malignant lesions predicts disease progression; Therefore, it is very important to evaluate some new lesions. There are no specific criteria for the identification of new radiographic lesions; however, the finding of a new lesion should be unequivocal: i.e. not attributable to differences in scanning technique, change in imaging modality or findings thought to represent something other than tumor (for example, some 'new' bone lesions may be simply healing or flare of preexisting lesions). This is particularly important when the patient's baseline lesions show partial or complete response. For example, necrosis of a liver lesion may be reported on a CT scan report as a 'new' cystic lesion, which it is not.

A lesion identified on a follow-up study in an anatomical location that was not scanned at baseline is considered a new lesion and will indicate disease progression. An example of this is the patient who has visceral disease at baseline and while on study has a CT or MRI brain ordered which reveals metastases. The patient's brain metastases are considered to be evidence of PD even if he/she did not have brain imaging at baseline.

If a new lesion is equivocal, for example because of its small size, continued therapy and follow-up evaluation will clarify if it represents truly new disease. If repeat scans confirm there is definitely a new lesion, then progression should be declared using the date of the initial scan.

While FDG-PET response assessments need additional study, it is sometimes reasonable to incorporate the use of FDG-PET scanning to complement CT scanning in assessment of progression (particularly possible 'new' disease). New lesions on the basis of FDG-PET imaging can be identified according to the following algorithm:

Negative FDG-PET at baseline, with a positive FDG-PET at follow-up is a sign of PD based on a new lesion.

No FDG-PET at baseline and a positive FDG-PET at follow-up:

If the positive FDG-PET at follow-up corresponds to a new site of disease confirmed by CT, this is PD.

If the positive FDG-PET at follow-up is not confirmed as a new site of disease on CT, additional follow-up CT scans are needed to determine if there is truly progression occurring at that site (if so, the date of PD will be the date of the initial abnormal FDG-PET scan).

If the positive FDG-PET at follow-up corresponds to a preexisting site of disease on CT that is not progressing on the basis of the anatomic images, this is not PD.

4.4 Evaluation of Best Overall Response

The best overall response is the best response recorded from the start of the study treatment until the end of treatment taking into account any requirement for confirmation. On occasion a response may not be documented until after the end of therapy so protocols should be clear if post-treatment assessments are to be considered in determination of best overall response. Protocols must specify how any new therapy introduced before progression will affect best response designation. The patient's best overall response assignment will depend on the findings of both target and non-target disease and will also take into consideration the appearance of new lesions. Furthermore, depending on the

nature of the study and the protocol requirements, it may also require confirmatory measurement. Specifically, in non-randomized trials where response is the primary endpoint, confirmation of PR or CR is needed to deem either one the best overall response.

4.4.1 Time Point Response

It is assumed that at each protocol specified time point, a response assessment occurs. A summary of the overall response at each time point for patients who have measurable disease at baseline is provided in Table 1.

When patients have non-measurable (therefore non-target) disease only, Table 2 is to be used.

4.4.2 Missing Assessments and Non-evaluable Designation

When no imaging/measurement is done at all at a particular time point, the patient is not evaluable (NE) at that time point. If only a subset of lesion measurements is made at an assessment, usually the case is also considered NE at that time point, unless a convincing argument can be made that the contribution of the individual missing lesion(s) would not change the assigned time point response. This would be most likely to happen in the case of PD. For example, if a patient had a baseline sum of 50mm with three measured lesions and at follow-up only two lesions were assessed, but those gave a sum of 80 mm, the patient will have achieved PD status, regardless of the contribution of the missing lesion.

4.4.3 Best Overall Response: all time points

The best overall response is determined once all the data for the patient is known.

Best response determination in trials where confirmation of complete or partial response IS NOT required: Best response in these trials is defined as the best response across all time points (for example, a patient who has SD at first assessment, PR at second assessment, and PD on last assessment has a best overall response of PR). When SD is believed to be best response, it must also meet the protocol-specified minimum time from baseline. If the minimum time is not met when SD is otherwise the best time point response, the patient's best response depends on the subsequent assessments. For example: a patient who has evaluation of SD in the first cycle, PD in the second cycle but does not meet minimum duration for SD, the best overall response is PD. Similarly if the patient is lost to follow-up after the evaluation of SD in the first cycle and it is considered as not evaluable.

Best response determination in trials where confirmation of complete or partial response IS required: Complete or partial responses may be claimed only if the criteria for each are met at a subsequent time point as specified in the protocol (generally 4 weeks later). In this circumstance, the best overall response can be interpreted as in Table 3.

4.4.4 Special Notes on Response Assessment

When nodal disease is included in the sum of target lesions and the nodes decrease to 'normal' size (<10 mm), they may still have a measurement reported on scans. This measurement should be recorded even though the nodes are normal in order not to overstate progression should it be based on increase in size of the nodes. As noted earlier, this means that patients with CR may not have a total sum of 'zero' on the case report form (CRF).

In trials where confirmation of response is required, repeated 'NE' time point assessments may complicate best response determination. The analysis plan for the trial must address

how missing data/assessments will be addressed in determination of response and progression. For example, in most trials it is reasonable to consider a patient with time point responses of PR-NE-PR as a confirmed response.

Patients with a global deterioration of health status requiring discontinuation of treatment without objective evidence of disease progression at that time should be reported as 'symptomatic deterioration'. Every effort should be made to assess objective progression even after discontinuation of treatment. Symptomatic deterioration is not a descriptor of an objective response: it is the reason for stopping study therapy. The objective response status of such patients is to be determined by evaluation of target and non-target disease as shown in Tables 1–3.

Conditions that define early progression, early death and unevaluability are study specific and should be clearly described in each protocol (depending on treatment duration, treatment cycle).

In some circumstances it may be difficult to distinguish local disease from normal tissue. When the evaluation of complete response depends upon this determination, it is recommended that biopsy be performed to investigate the local disease before assigning a status of complete response. FDG-PET may be used to upgrade a response to a CR in a manner similar to a biopsy in cases where a residual radiographic abnormality is thought to represent fibrosis or scarring. The use of FDG-PET in this circumstance should be prospectively described in the protocol and supported by disease-specific medical literature for the indication. However, it must be acknowledged that both approaches may lead to false positive CR due to limitations of FDG-PET and biopsy (resolution/sensitivity).

Table 1 Time Point Response: Patients with Target (+/- Non-target) Lesions

Target lesions	Non-target lesions	New lesions	Overall response
CR	CR	No	CR
CR	Non-CR/non-PD	No	PR
CR	NE	No	PR
PR	Non-PD or not all evaluated	No	PR
SD	Non-PD or not all evaluated	No	SD
Not all evaluated	Non-PD	No	NE
PD	Any	Yes or No	PD
Any	PD	Yes or No	PD
Any	Any	yes	PD
CR = complete response	PR = partial response	SD = stable disease	PD = progression of disease<br>NE = not evaluated

Table 2 Time Point Response: Patients with Non-target Disease Only

Non-target lesions	New lesions	Overall response
CR	No	CR
Non-CR/non-PD	No	Non-CR/non-PD
Not all evaluated	No	NE
Unequivocal PD	Yes or No	PD
Any	yes	PD

Notes: ‘Non-CR/non-PD’ is preferred over ‘stable disease’ for non-target disease since SD is increasingly used as endpoint for assessment of efficacy in some trials so to assign this category when no lesions can be measured.

Treatment for equivocal findings of progression (e.g., very small indeterminate new lesions; cystic degeneration or necrosis of pre-existing lesions) may continue until the next assessment. If progression is confirmed at the next scheduled assessment, the date of progression should be the earlier date when progression was suspected.

Table 3 Best overall response when confirmation of CR and PR required

Overall response - First time point	At Subsequent Time Points Overall response	Best overall response
CR	CR	CR
CR	PR	SD, PD or PR[a]
CR	SD	SD provided minimum criteria for SD duration met, otherwise, PD
CR	PD	SD provided minimum criteria for SD duration met, otherwise, PD
CR	NE	SD provided minimum criteria for SD duration met, otherwise NE
PR	CR	PR
PR	PR	PR
PR	SD	SD
PR	PD	SD provided minimum criteria for SD duration met, otherwise, PD
PR	NE	SD provided minimum criteria for SD duration met, otherwise NE
NE	NE	NE

Notes: CR = complete response; PR = partial response; SD = stable disease; PD = progressive disease; and NE = unevaluable. a: If a CR is truly met at first time point, then any disease seen at a subsequent time point, even disease meeting PR criteria relative to baseline, makes the disease PD at that point (since disease must have reappeared after CR). Best response would depend on whether minimum duration for SD was met. However, sometimes 'CR' may be claimed when subsequent scans suggest small lesions were likely still present and in fact the patient had PR, not CR at the first time point. Under these circumstances, the original CR should be changed to PR and the best response is PR.

4.5 Frequency of Tumor Re-evaluation

Frequency of tumor re-evaluation while on treatment should be protocol-specific and adapted to the type and schedule of treatment. However, in the context of phase II studies where the beneficial effect of therapy is not known, follow-up every 6–8 weeks (timed to coincide with the end of a cycle) is reasonable. Smaller or greater time intervals than these could be justified in specific regimens or circumstances. The protocol should specify which organ sites are to be evaluated at baseline (usually those most likely to be involved with metastatic disease for the tumor type under study) and how often evaluations are repeated. Normally, target and non-target lesions are evaluated at each assessment. In selected circumstances, certain non-target organs may be evaluated less frequently. For example, bone scans may need to repeated only when CR is identified in target disease is or when progression in bone is suspected.

After the end of the treatment, the need for repetitive tumor evaluations depends on whether the trial has as a goal the response rate or the time to an event (progression/death). If 'time

to an event' (e.g. time to progression, disease-free survival, progression-free survival) is the main endpoint of the study, then routine scheduled re-evaluation of protocol specified sites of disease is warranted. In randomized comparative trials in particular, the scheduled assessments should be performed as identified on a calendar schedule (for example: every 6 - 8 weeks on treatment or every 3 - 4 months after treatment) and should not be affected by delays in therapy, drug holidays or any other events that might lead to imbalance in a treatment arm in the timing of disease assessment.

4.6 Confirmatory Measurement/Duration of Response

4.6.1 Confirmation

In non-randomized clinical studies where response is the primary endpoint, confirmation of PR and CR is required to ensure responses identified are not the result of measurement error. This will also permit appropriate interpretation of results in the context of historical data where response has traditionally required confirmation in such trials. However, in all other circumstances, i.e. in randomized trials (phase II or III) or studies where stable disease or progression is the primary endpoint, confirmation of response is not required since it will not add value to the interpretation of trial results. However, elimination of the requirement for response confirmation may increase the importance of central review to protect against bias, in particular in studies which are not blinded.

In the case of SD, measurements must have met the SD criteria at least once after study entry at a minimum interval (in general not less than 6 - 8 weeks) that is defined in the study protocol.

4.6.2 Duration of Overall Response

The duration of overall response is measured from the time measurement criteria are first met for CR/PR (whichever is first recorded) until the first date that recurrent or progressive disease is objectively documented (taking as reference for progressive disease the smallest measurements recorded on study). The duration of overall complete response is measured from the time measurement criteria are first met for CR until the first date that recurrent disease is objectively documented.

4.6.3 Duration of Stable Disease

Stable disease is measured from the start of the treatment (in randomized trials, from date of randomization) until the criteria for progression are met, taking as reference the smallest sum on study (if the baseline sum is the smallest, this is the reference for calculation of PD). The clinical relevance of the duration of stable disease varies in different studies and diseases. If the proportion of patients achieving stable disease for a minimum period of time is an endpoint of importance in a particular trial, the protocol should specify the minimal time interval required between two measurements for determination of stable disease.

Notes: The duration of response and stable disease as well as PFS are influenced by the frequency of follow-up after baseline evaluation. It is not in the scope of this guideline to define a standard follow-up frequency. The frequency should take into account many parameters including disease types and stages, treatment cycle and standard practice. However, these limitations of the precision of the measured endpoint should be taken into account if comparisons between trials are to be made.

4.7 Progression-free Survival/Time to Progression (PFS/TTP)

4.7.1 Phase II Clinical Trials

This guideline is focused primarily on the use of objective response endpoints for phase II trials. In some circumstances, 'response rate' may not be the optimal method to assess the potential anticancer activity of new agents/regimens. In such cases, PFS or the proportion progression-free (PPF) at landmark time points, might be considered appropriate alternatives to provide an initial signal of biologic effect of new agents. It is clear, however, that in an uncontrolled trial, these measures are subject to criticism since an apparently promising observation may be related to biological factors such as patient selection and not the impact of the intervention. Thus, phase II screening trials utilizing these endpoints are best designed with a randomized control. Exceptions may exist where the behavior patterns of certain cancers are so consistent (and usually consistently poor), that non-randomized trial is justifiable. However, in these cases it will be essential to document with care the basis for estimating the expected PFS or PPF in the absence of an active control.

For the endpoints evaluation, independent assessment and result reporting concerning the Phase III trials, please see the English version.

Appendix 7 New York Heart Association (NYHA) Functional Classification

Grade	Physical activity	Quiescent condition	Symptoms (fatigue, palpitation, asthma or angina)
I	Unlimited	Asymptomatic	Not caused by general physical activity
II	Slightly Limited	Asymptomatic	Caused by ordinary physical activity
III	Significantly limited	Asymptomatic	Caused by less than ordinary physical activity
IV	Unable	Symptomatic	Increased after any physical activities

Appendix 8 Cockcroft and Gault formula

FOR SERUM CREATININE CONCENTRATION (SCr) IN MG/DL[a]

Cl_{cr} for males (mL/min) $$\frac{(140\text{-age})(\text{weight}^{b})}{(72)\ (\text{SCr})}$$

CL_{cr} for females (mL/min) $$\frac{(0.85)(140\text{-age})(\text{weight}^{b})}{(72)\ (\text{SCr})}$$

FOR SERUM CREATININE CONCENTRATION (SCr) IN µMOL/L[a]

Cl_{cr} for males (mL/min) $$\frac{(140\text{-age})(\text{weight}^{b})}{(0.81)(\text{SCr})}$$

CL_{cr} for females (mL/min) $$\frac{(0.85)(140\text{-age})(\text{weight}^{b})}{(0.81)(\text{SCr})}$$

Legends:

- Age is age.
- Weight is the weight in kilograms.
- Obese patients: The following formula (lean mass formula) is used when a patient has BMI >30:
  1. Ccr (male) = $[(137\text{-age}) \times (0.285 \times \text{weight (kg)} + 12.1 \times \text{height}^{2}\text{ (m)})]/51 \times \text{Scr}$ (mg/dl)
  2. Ccr (female) = $[(146\text{-age}) \times (0.287 \times \text{weight (kg)} + 9.74 \times \text{height}^{2}\text{ (m)})]/60 \times \text{Scr}$ (mg/dl)

Notes:

1. Blood creatinine: 1 umol/L = 88.4 mg/dl
2. BMI = weight (kg)/height ^{2}(m)

Appendix 9 Fridericia's formula

$$QT_F = \frac{QT}{\sqrt[3]{RR}}$$

QT is defined as the interval between the start of Q wave and the end of T wave

RR is defined as the interval between the occurrence of QRS complex and the occurrence of the next QRS complex.

Appendix 10. List of Prohibited QT Interval-Prolonging Medications

Drug	QT risk (*)
Amiodarone	Known risk of TdP
Arsenic trioxide	Known risk of TdP
Astemizole	Known risk of TdP
bepridil	Known risk of TdP
Chloroquine	Known risk of TdP
Chlorpromazine	Known risk of TdP
Cisapride	Known risk of TdP
Disopyramide	Known risk of TdP
Dofetilide	Known risk of TdP
domperidone	Known risk of TdP
droperidol	Known risk of TdP
Halofantrine	Known risk of TdP
Haloperidol	Known risk of TdP
Ibutilide	Known risk of TdP
Levomethadone	Known risk of TdP
Mesoridazine	Known risk of TdP
Methadone	Known risk of TdP
Pentamidine	Known risk of TdP
pimozide	Known risk of TdP
Probucol	Known risk of TdP
Procainamide	Known risk of TdP
Quetiapine	Possible risk of TdP
Quinidine	Known risk of TdP
Sotalol	Known risk of TdP
Sparfloxacin	Known risk of TdP
tacrolimus	Possible risk of TdP
Terfenadine	Known risk of TdP
Thioridazine	Known risk of TdP
vardenafil	Possible risk of TdP

For medications that are known to prolong QT interval or be potentially associated with torsade de pointes (TdP), please visit: http://www.crediblemeds.org/everyone/composite-list-all-qtdrugs/?rf=All

Appendix 11 List of QT Interval-Prolonging Drugs to be Used with Caution

**Drug**	**QT risk (*)**
Alfuzosin	Possible risk of TdP
Amantadin	Possible risk of TdP
amitriptyline	Conditional risk of TdP
Azithromycin	Possible risk of TdP
hydrate	Possible risk of TdP
citalopram	Conditional risk of TdP
Clomipramine	Conditional risk of TdP
clozapine	Possible risk of TdP
Desipramine	Conditional risk of TdP
diphenhydramine	Conditional risk of TdP
Dolasetron	Possible risk of TdP
Doxepin	Conditional risk of TdP
Dronedarone	Possible risk of TdP
Felbamate	Possible risk of TdP
Flecainide	Possible risk of TdP
Fluoxetine	Conditional risk of TdP
Foscarnet Sodium	Possible risk of TdP
Fosphenytoin	Possible risk of TdP
Galanthamine	Conditional risk of TdP
Gatifloxacin	Possible risk of TdP
Gemifloxacin	Possible risk of TdP
Granisetron	Possible risk of TdP
Imipramine	Conditional risk of TdP
Indapamide	Possible risk of TdP
Isradipine	Possible risk of TdP
Levofloxacin	Possible risk of TdP
Lithium	Possible risk of TdP
MEXITIL	Conditional risk of TdP
Moexipril /HCTZ	Possible risk of TdP

Moxifloxacin	Possible risk of TdP
Nicardipine	Possible risk of TdP
Nortriptyline	Conditional risk of TdP
Octreotide	Possible risk of TdP
Ofloxacin	Possible risk of TdP
Ondansetron	Possible risk of TdP
Oxytocin	Possible risk of TdP
Paliperidone	Possible risk of TdP
Paroxetine	Conditional risk of TdP
Perfluoropropane Lipid Microspheres	Possible risk of TdP
protriptyline	Conditional risk of TdP
Ranolazine	Possible risk of TdP
Risperidone	Possible risk of TdP
Roxithromycin*	Possible risk of TdP
Sertindole	Possible risk of TdP
Sertraline	Conditional risk of TdP
Solifenacin	Conditional risk of TdP
tizanidine	Possible risk of TdP
Trazodone	Conditional risk of TdP
Compound Sulfamethoxazole	Conditional risk of TdP
Trimipramine	Conditional risk of TdP
Venlafaxine	Possible risk of TdP
Ziprasidone	Possible risk of TdP
For medications that are known to prolong QT interval or be potentially associated with torsade de pointes (TdP), please visit: http://www.crediblemeds.org/everyone/composite-list-all-qtdrugs/?rf=All	

Supplementary Note 2

Statistical Analysis Plan

A multi-center, open-label, single-arm dose escalation and expansion Phase I clinical study to evaluate the safety, tolerability, pharmacokinetic characteristics and preliminary efficacy of CYH33 in patients with advanced solid tumors

**Sponsor:**	**Haihe Biopharma Co.,Ltd.**
**Protocol No.:**	***CYH33-101***
**Version:**	**0.8**
**Version Date:**	**June 25, 2021**
**Author:**	**Xiaolin Liu**

Signature Page

A multi-center, open-label, single-arm dose escalation and expansion Phase I clinical study to evaluate the safety, tolerability, pharmacokinetic characteristics and preliminary efficacy of CYH33 in patients with advanced solid tumors

Protocol No.: ***CYH33-101, Version 5.0, dated September 24, 2019***

Version: **5.0**

Written by

____

Xiaolin Liu Date:____________

Senior Biostatistician

Parexel International

Reviewed by

____

Sean Chang Date:____________

Associate Director, Biostatistics

Parexel International

Approved by

____

Jerry Wu Date:____________

Senior Director, Biostatistics and Data Science

Haihe Biopharma Co.,Ltd.

Amendment History

Version	*Date*	*Author*	*Description*
1.0	2019-XX-XX	XX	XX

Table of Contents

Page No.

1. Introduction 8
2. Study Objectives 8
   2.1 Primary Objectives 8
   2.2 Secondary Objectives 8
   2.3 Exploratory Objectives 8
3. Study Plan 9
   3.1 Study Design 9
   3.2 Sample Size 12
4. Study Endpoints and Endpoint Measures 14
   4.1 Study Endpoints 14
   4.2 Subgroup Analyses 16
5. Statistical Analysis Assumptions 16
6. Definition of Variables 16
   6.1 DLT Analysis Set (only applicable to Phase Ia) 18
   6.2 Full Analysis Set (FAS) 18
   6.3 Efficacy Evaluable Analysis Set (EAS) 19
   6.4 Safety Set (SS) 19
   6.5 PK Analysis Set 19
   6.6 PD Analysis Set 19
7. Analysis Plan 19
   7.1 Interim Analysis 19
   7.2 Primary Analyses 19
   7.3 Final Analysis 19
8. Statistical Analysis Methods 19
   8.1 General Principles 19
   8.2 Patient Disposition 20
   8.3 Protocol Violation 20

8.4 Analysis Set 21
8.5 Demographic and Other Baseline Characteristics 21
8.6 Efficacy Analysis 23
8.6.1 Objective Response Rate (ORR), Disease Control Rate (DCR) and Clinical Benefit Rate (CBR) 23
8.6.2 Progression-Free Survival (PFS), Duration of Response (DoR) and Overall Survival (OS) 23
8.6.3 Subgroup Analyses 24
8.7 Safety Analysis 24
8.7.1 Dose-limiting Toxicity (DLT) 24
8.7.2 Adverse Events 24
8.7.3 Laboratory Tests 26
8.7.4 Other Safety Tests 27
8.7.5 Medication Information and Treatment Compliance 27
8.7.6 Prior and Concomitant Medications 28
8.8 Additional Analysis 28
8.8.1 PK Analysis 28
8.8.2 Biomarker Analysis 28
9. Analyses Different From those Described in the Study Protocol 28
10. References 29
11. Appendices 29

Tables

Table 1.Imputation Rules for Partial or Missing Start Dates 30
Table 2. Grading of Select Laboratory Parameters 32

Appendices

Appendix 1.Handling of Dates, Incomplete Dates and Missing Dates for Adverse Events and Concomitant Medications 30
Appendix 2.Handling of Dates, Incomplete Dates and Missing Dates for Adverse Events and Concomitant Medications 32

Abbreviations

Abbreviations	Full text
AE	Adverse event
$ARAUC_{(0\text{-}24h)}$	Accumulation ratio of area under concentration-time curve from time 0 to 24 h
ARC_{max}	Accumulation ratio of maximum plasma concentration
$AUC_{(0\text{-}24h)}$	Area under concentration-time curve from time 0 to 24 h
$AUC_{0\text{-}\infty}$	Area under concentration-time curve from time 0 (before administration) to infinity (∞)
$AUC_{0\text{-}last}$	Area under concentration-time curve from time point 0 (before administration) to last time point with measurable concentration
AUC0-t	Area under the concentration-time curve from time 0 (before administration) to the last time point with measurable concentration
CBR	Clinical benefit rate
CL/F	Apparent clearance
C_{max}	Maximum plasma concentration
CR	Complete response
DCR	Disease control rate
DLT	Dose-limiting toxicity
DoR	Duration of response
ECG	Electrocardiogram
ECHO	Echocardiograms
ECOG	Eastern Cooperative Oncology Group
CRF	Case Report FormV
HIV	Human immunodeficiency virus
ICF	Informed Consent Form
ICH-E9	Statistical Principles for Clinical Trials (Chinese Version)
LLN	Lower limit of normal
MTD	Maximum tolerated dose
MUGA	Multiple uptake gated acquisition scan
NCI CTCAE	National Cancer Institute-Common Terminology Criteria for Adverse Events
NYHA	New York Heart Association
ORR	Objective response rate
OS	Overall survival
PD	Pharmacodynamics
PFS	Progression free survival
PK	Pharmacokinetics
PR	Partial response
RECIST	Response Evaluation Criteria in Solid Tumors

SAE	Serious adverse event
SAP	Statistical Analysis Plan
SD	Stable disease
SMC	Data Safety Monitoring Committee
$t_{1/2}$	Elimination half-life
TEAE	Treatment-emergent adverse event
TESAE	Treatment-emergent serious adverse event
t_{max}	Time to maximum plasma concentration
ULN	Upper limit of normal
Vz/F	Apparent volume of distribution

1. Introduction

The Statistical Analysis Plan (SAP) details the statistical methods used to analyze the study data. The analytical methods are based on the CYH33-101 study protocol, version 5.0, dated 29 May 2020, and the electronic case report form (eCRF), version 3.0, dated 09 December 2019. This plan describes the analysis methods of interim analysis, primary analysis, and final analysis, as well as the determination of sample size, indicators and populations, transformation and operation of expected data, descriptive analysis, statistical modeling and other details.

Biomarker analyses will be described in a separate analysis plan.

2. Study Objectives

2.1 Primary Objectives

- Phase Ia: To determine the safety, tolerability and maximum tolerated dose (MTD) of oral CTH33 monotherapy in patients with advanced solid tumors who have failed or cannot tolerate standard treatment or currently have no standard treatment, and determine the recommended Phase 2 dose (RP2D).

[redacted]

2.2 Secondary Objectives

- Phase Ia: To assess the preliminary efficacy of oral CYH33 monotherapy in patients with advanced solid tumors who have failed or cannot tolerate standard treatment or currently have no standard treatment.
- Phase Ia: To determine the pharmacokinetic (PK) characteristics of CYH33 and its metabolite (I27) after single and continuous oral administration of CYH33.

[redacted]

[redacted]

[redacted]

2.3 Exploratory Objectives

- To explore the relationship between the efficacy of CYH33 treatment and potential tumor biomarkers.

3. Study Plan

3.1 Study Design

This is a multi-center, open-label, single-arm Phase I clinical study of dose escalation and expansion of oral CYH33 monotherapy in patients with advanced solid tumors who have failed or cannot tolerate standard treatment or currently have no standard treatment. The study population consists of patients with advanced solid tumors who have failed or cannot tolerate standard treatment or currently have no standard therapy in Phase Ia dose escalation part,patients with PIK3CA-mutated advanced solid tumors who have failed or cannot tolerate standard treatment or currently have no standard therapy in Phase Ia dose expansion part. [redacted] Patients must sign written informed consent and provide an unstained, radiation-naïve tumor tissue section, or/and formalin-fixed paraffin-embedded block or/and fresh tumor tissue to the central laboratory for confirming *PIK3CA* gene mutation status prior to screening.

This study consists of a Phase Ia dose escalation part, Phase Ia dose expansion part. In Phase Ia, one or more safe and considerably effective dose will be selected for the Phase Ia expansion study. DLT events will continue to be observed in the DLT observation phase. At any dose level or if necessary, the SMC can decide to enter the Ia expansion phase at this dose level to further investigate the safety, tolerability, and efficacy of this dose level combined with the effective dose predicted with the PK/PD model. The subjects enrolled in the Phase Ia dose expansion part are patients with PIK3CA-mutated advanced solid tumors. The total number of patients at each dose level will be decided by the SMC based on the proportions of patients with CR, PR and SD, as well as the safety and tolerability. The recommended Phase II dose (RP2D) will be determined through the Phase Ia dose expansion. [redacted]

Phase Ia Dose Escalation

Phase Ia dose escalation study was completed in February 2020. Specific study methods are described in CYH33-101 study protocol version 5. The MTD observed in Phase Ia dose escalation part was determined to be 40 mg after reviewing by the SMC. The definition and assessment of dose-limiting toxicities in Phase Ia are described in Section 3.2.1 of protocol version 5 A list of study assessments is provided in attachments 1 and 2 in protocol version 5.

Phase Ia Dose Expansion

During the Phase Ia study, the safe and effective dose will be selected for the Phase Ia expansion study, while the Phase Ia dose escalation part can be continued simultaneously. At any dose level or if necessary, the SMC can decide to enter the expansion study at this dose level to further assess the safety, tolerability and efficacy combined with the effective dose predicted by the PK/PD model. The subjects enrolled in the expansion study are patients with P1K3CA-mutated advanced solid tumors. The total number of patients at each dose level will be decided by the SMC based on the proportions of patients with CR, PR and SD, as well as the safety and tolerability.

[redacted]

[redacted]

[redacted]

[redacted]

[redacted]	[redacted]	[redacted]	[redacted]
[redacted]	[redacted]	[redacted]	[redacted]

[redacted]

[redacted]	[redacted]	[redacted]	[redacted]	[redacted]
	[redacted]	[redacted]	[redacted]	[redacted]
	[redacted]	[redacted]	[redacted]	
[redacted]	[redacted]	[redacted]	[redacted]	[redacted]
	[redacted]	[redacted]	[redacted]	[redacted]

[redacted]

[redacted]	[redacted]	[redacted]	[redacted]
[redacted]	[redacted]	[redacted]	[redacted]

[redacted]

[redacted]	[redacted]	[redacted]	[redacted]
[redacted]	[redacted]	[redacted]	[redacted]

[redacted]

[redacted]

[redacted]	[redacted]	[redacted]	[redacted]	[redacted]
[redacted]	[redacted]	[redacted]	[redacted]	
	[redacted]	[redacted]	[redacted]	[redacted]

[redacted]

[redacted]	[redacted]	[redacted]	[redacted]
[redacted]	[redacted]	[redacted]	

<u>End of Study</u>

The last enrolled patient completes two tumor assessments after the first dose, has disease progression or unacceptable toxicity, withdraws from the study or dies, whichever occurs first.

<u>Data Safety Monitoring Committee (SMC)</u>

During the study, an SMC composed of coordinating investigators, the principal investigator of the enrolling site, independent clinical oncologists (if necessary), biostatistician, drug PK expert, the sponsor's medical monitors and drug safety physicians will be established to review the safety, efficacy and PK/PD data arising from the study, decide the assignment of patients, dose levels and dosing frequency in each dose group in Phase Ia dose escalation part, and recommend and choose doses for entering the Phase Ia expansion part, so as to determine the MTD/RP2D. [redacted]

3.2 Sample Size

There are approximately 60 evaluable patients in Phase Ia. [redacted]
The actual number of subjects and dose escalation level will be discussed and decided by SMC based on the obtained safety data and/or PK/PD modeling simulation data.

[redacted]

[redacted]

[redacted]

[redacted]

[redacted]

[redacted]

[redacted]

[redacted]

[redacted]	[redacted]	[redacted]	[redacted]	[redacted]
[redacted]	[redacted]	[redacted]	[redacted]	[redacted]
[redacted]	[redacted]	[redacted]	[redacted]	[redacted]
	[redacted]	[redacted]	[redacted]	
	[redacted]	[redacted]	[redacted]	[redacted]
	[redacted]	[redacted]	[redacted]	[redacted]
[redacted]	[redacted]	[redacted]	[redacted]	[redacted]
[redacted]	[redacted]	[redacted]	[redacted]	[redacted]
[redacted]	[redacted]	[redacted]	[redacted]	
	[redacted]	[redacted]	[redacted]	[redacted]
[redacted]	[redacted]	[redacted]	[redacted]	
[redacted]	[redacted]			

4. Study Endpoints and Endpoint Measures

4.1 Study Endpoints

Primary Endpoint(s):

- Phase Ia: Type and frequency of treatment-emergent adverse events (TEAEs), and evaluation of toxicity graded according to NCI CTCAE version 4.03: clinically significant laboratory test results, electrocardiogram (ECG) and cardiac imaging findings, and physical examination findings (including vital signs, weight, and ECOG performance status score), etc.
- Phase Ia: Number and proportion of subjects with dose-limiting toxicity (DLT) in DLT observation phase (within about 28 days after the first dose, 35 days after the first dose for patients receiving a single dose, i.e. 7 days in the single dose phase + 28 days of Cycle 1 of the continuous dose phase) (Phase Ia dose escalation part and dose expansion part).
- Phase Ia: Maximum tolerated dose (MTD) - if MTD is not observed, the RP2D will be determined through PK (pharmacokinetic)/PD (pharmacodynamic) data, safety and preliminary efficacy.

[redacted]

Secondary Endpoint(s):

- Phase Ia: Response assessment endpoints - objective response rate (ORR), progression-free survival (PFS), duration of response (DoR) and disease control rate (DCR), clinical benefit rate (CBR).
- Phase Ia: PK parameters of CYH33 and its metabolite (I27), including:
    - Area under concentration-time curve from time point 0 (pre-dose) to last time point with measurable concentration ($AUC_{0\text{-last}}$)
    - Area under concentration-time curve from time 0 to 24 h ($AUC_{0\text{-24h}}$)
    - Area under concentration-time curve from time 0 (pre-dose) to infinity (∞) ($AUC_{0\text{-}\infty}$)
    - Maximum plasma concentration (C_{max})
    - Time to maximum plasma concentration (t_{max})
    - Elimination half-life ($t_{1/2}$)
    - Apparent volume of distribution (V_z/F)
    - Apparent clearance (CL/F)
    - Accumulation ratio of maximum plasma concentration (ARC_{max})
    - Accumulation ratio of area under concentration-time curve from time 0 to 24 h ($ARAUC_{0\text{-24h}}$)

- o Linearity index (LI)

[redacted]

Exploratory Endpoint(s):

- [redacted]).

[redacted]

5. Statistical Analysis Assumptions

Not applicable.

6. Definition of Variables

Age

Age (years) = rounded [(signature date of ICF - date of birth + 1)/365.25]

Age is recorded on the "Demographic" page of the CRF.

Reproducible measurements

If a variable is measured more than once at the same visit according to the study protocol, it is defined as a reproducible measurement. The results of the reproducible measurements will be averaged for statistical analysis.

Baseline

Baseline is defined as the last available pre-treatment assessment. If the assessment date is the same as the first dose date, the assessment will be performed by time. If there is no assessment time, this assessment will be considered a "baseline" assessment.

Date of death

The date of death is recorded on the "Death" page in the CRF.

Date of enrollment

Enrollment date is defined as the date of the first dose.

Date of the last dose

The last dose date is defined as the date of the last dose of study medication and is recorded on the "End of Treatment" page in the CRF.

Study Day

If the event or assessment occurred on or after the first dose:

Study day = date of the event or assessment - date of the first dose + 1

If the event or assessment occurred before the first dose:

Study day = date of the event or assessment - date of the first dose

Dose-limiting toxicity (DLT)

DLT is defined as an adverse event or laboratory abnormality that occurs within 28 days after the first dose in Phase Ia (within 35 days after the first dose for patients receiving a single administration, i.e. 7 days in the single dose phase + 28 days of Cycle 1 of the continuous dose phase). See Table 1 of the study protocol for specific definitions.

MTD

MTD is the maximum tolerated dose, defined as the maximum dose at which the incidence of DLTs is less than 33% in Phase Ia dose escalation part.

RP2D

RP2D is the recommended Phase II dose and will be determined in light of Phase Ia safety, efficacy, and PK/PD data, regardless of MTD observed. [redacted]

Best response

Efficacy evaluation endpoints are determined according to guidelines (ICH E9, 1998, EMEA Guidelines, 2005 [1]). Patient's best response is defined as the best overall response from enrollment to end of treatment as judged by the investigator according to RECIST v1.1.

ORR

ORR is defined as the proportion of patients with a second confirmed complete response (CR) or partial response (PR) as evaluated according to RECIST v1.1.

PFS

PFS is defined as the time from the first dose of the study medication to disease progression or death, whichever is earlier. Patients without events (no progression or death) will be censored at the date of the last tumor assessment. For patients lacking post-baseline efficacy evaluation, censoring will be made at the date of the first dose. The date of tumor assessment is based on the date of radiography.

OS

OS is defined as the overall survival time from the first dose of study medication to death for any reason. For patients without death events, censoring will be made at the last date when the patient's survival information is obtained.

DoR

DoR is defined as the time from first CR or PR to tumor progression or death due to any reason, whichever occurs first. If no tumor progression or death is observed, censoring will be made at the date of the last tumor assessment. The date of tumor assessment is based on the date of radiography.

DCR

DCR is defined as the proportion of patients with confirmed CR, PR and SD lasting for ≥ 6 weeks.

CBR

CBR is defined as the proportion of patients with confirmed CR, PR and SD lasting for ≥ 24 weeks.

Treatment-emergent adverse events (TEAEs)

Treatment-emergent adverse events (TEAEs) are defined as adverse events that occur between the first dose and the last dose + 37 days or present at baseline but worsen after dosing. Treatment-emergent serious adverse events (TESAEs) are also subject to this definition.

Analysis Set

6.1 DLT Analysis Set [redacted]

Patients who experienced DLT within 28 days after the first dose (within 35 days after the first dose for patients receiving a single administration), or patients who have received >=75% of the study medication within 28 days of Cycle 1 of the continuous dose phase as planned.

6.2 Full Analysis Set (FAS)

Enrolled patients who have taken at least one dose of CYH33. The full analysis set will be used for baseline data analysis of Phase Ia.[redacted]

6.3 Efficacy Evaluable Analysis Set (EAS)

Patients who have taken at least one dose of CYH33 with baseline tumor assessment data and at least once post-baseline tumor assessments. The efficacy evaluable analysis set will be used for the efficacy evaluation analysis of Phase Ia. [redacted]

6.4 Safety Set (SS)

Patients who have taken at least one dose of CYH33.

6.5 PK Analysis Set

Including patients who have received at least one dose of CYH33 and have at least one evaluable PK data. Subjects with protocol violations seriously affecting the PK assessment results will be excluded from the PK analysis set.

[redacted]

[redacted]

7. Analysis Plan

7.1 Interim Analysis

Not applicable.

[redacted]

[redacted]

7.3 Final Analysis

After the database is locked, the final analysis may be performed at the end of the study when all subjects have completed the follow-up or withdrawn from the study, whichever occurs first. PFS, OS, and safety analyses will be updated.

8. Statistical Analysis Methods

8.1 General Principles

Unless otherwise specified, continuous variables will be summarized by the mean, standard deviation (SD), median, minimum, maximum, and number of observations. The minimum and maximum reported will retain the same decimal places as the original data recorded in the database. The mean, median, lower quartile, and upper quartile will be rounded to one more decimal place than the raw data. The standard deviation will be kept to two more decimal places than the raw data recorded in the database. In general, any summary statistics will be rounded to a maximum of four decimal places.

Categorical data will be provided by number, frequency, and percentage of subjects by time point. If required, SAP text and TFL files will detail any planned category merges.

Percentages will be rounded to one decimal place. Percentages are not shown for zero counts. Percentages will be calculated with n as the denominator. If the sample size is small, data percentages will be provided, but only frequencies will be described in any report text.

In general, P values greater than or equal to 0.001 will be kept to three decimal places. P values less than 0.001 are indicated as "< 0.001."

Confidence intervals will be rounded to one more decimal place than the raw data.

Missing data will not be imputed. Handling of missing dates for judging treatment-emergent adverse events (TEAEs) and concomitant medications (CMs) is described in Appendix 1.

All summary analyses will be performed by dose level (Phase Ia) [redacted] unless otherwise specified.

All report outputs will be generated using SAS® version (9.3 or higher) in a safe and effective environment.

8.2 Patient Disposition

A description of the disposition of all subjects participating in the study from screening to the end of the study will be provided, and the number and percentage of subjects will be provided for the following subjects:

- Screening
- Screening failure with the main reason
- Enrollment
- Treatment; no treatment
- Subjects on treatment
- Subjects with premature discontinuation with the main reason
- Subjects under study
- Subjects who prematurely withdrew from the study with the main reason

A listing of subject disposition will be provided. The listing will include subject ID, age/gender, screening date, screening result, treatment/no treatment, first dose date, last dose date, whether the treatment is completed, prematurely terminated with the main reason, and whether the study is completed, prematurely terminated with the main reason.

8.3 Protocol Violation

Protocol violations will be analyzed based on the full analysis set (FAS).

Major protocol violations are those that may affect the known efficacy and/or safety of the study treatment. The purpose of assessing the robustness of the study results and conclusions for the selected analysis sets is to investigate the impact of major protocol violations on efficacy and/or safety outcomes, and to consider excluding data that may have been affected by important protocol violations.

Major protocol deviations and any actions taken to exclude subjects or impacted data from a particular analysis have been defined in the protocol specifications of the project. They should be determined by a data review meeting prior to database lock.

The number and percentage of subjects with at least one major protocol violation will be provided and the number and percentage of subjects involved in various protocol violations.

A listing of major protocol deviations will be provided. The listing will include subject ID, age/gender, protocol violation category, collection date, and the description of the protocol violation.

8.4 Analysis Set

Summaries of the analysis set will be based on the full analysis set (FAS).

The number and percentage of subjects in the DLT analysis set, the full analysis set, the efficacy evaluable analysis set, and the safety analysis set will be summarized.

A listing of subject analysis sets will be provided. The listing will include subject ID, inclusion in each analysis set, and reasons for exclusion.

8.5 Demographic and Other Baseline Characteristics

Summaries of demographic and other baseline characteristics will be based on the full analysis set (FAS). The following variables will be summarized and tabulated. Descriptive statistical analyses will be performed for continuous variables. The number and percentage of subjects in each category will be counted for categorical variables.

Demographic and baseline characteristics:

- Age, age group (< 65, ≥ 65, ≥ 75)
- Gender, ethnicity, race
- Height and weight at baseline
- History of smoking and alcohol
- Baseline NYHA class
- Baseline serology: hepatitis B surface antigen, hepatitis C antibody, HIV antibody
- ECOG score at screening

History of cancer:

- Diagnostic results
- Time from diagnosis to enrollment (years) = (date of first dose-date of diagnosis + 1)/365.25
- Disease stage at diagnosis
- Pathological classification
- Disease stage at enrollment, T-category, N-category, M-stage
- Primary tumor status at enrollment
- PIK3CA mutation status by local report.

History of diabetes:

- Whether there is a history of diabetes, type
- Diabetes status at enrollment, diabetes treatment at enrollment, CTCAE classification at screening
- Whether there is a history of impaired glucose tolerance, current treatment for impaired glucose tolerance

Past medical history:

The past medical history will be coded using the Medical Dictionary for Regulatory Activities (MedDRA) version 21.1 or higher. It will be summarized by system organ class (SOC) and preferred term (PT).

The CTCAE classification of past medical history and ongoing treatment will be presented in the listings.

Prior anti-cancer therapy:

The following variables will be summarized:

- Whether there is a history of prior anti-cancer therapy
- Number of lines and type of treatment
- Best response
- Treatment medications will be coded using the World Health Organization Drug Dictionary Enhanced (WHO-DDE, version September 2018 or latest) and summarized categorically by Anatomical Therapeutic Chemical (ATC) drug class and preferred term (PT).

Prior anti-cancer surgeries and procedures will be coded using the MedDRA version 21.1 or higher. The purpose of the surgery or procedure and the SOC and PT will be summarized.

Prior anti-cancer radiation therapy will be based on prior radiation therapy, treatment site, and best response.

Past medical history and treatment history will be provided in a listing with detailed information.

8.6 Efficacy Analysis

The efficacy analysis is a secondary study endpoint. In Phase Ia, response assessment endpoints include objective response rate (ORR), progression-free survival (PFS), duration of response (DoR) and disease control rate (DCR), and clinical benefit rate (CBR). The analyses will be performed separately for the overall population, the PIK3CA mutation-harboring population, and across tumor types (including breast, ovarian, cervical, colorectal and other tumors). [redacted]

Efficacy analyses will be based on the evaluable analysis set.

8.6.1 Objective Response Rate (ORR), Disease Control Rate (DCR) and Clinical Benefit Rate (CBR)

The number and percentage of confirmed best responses will be summarized:

- Complete response (CR)
- Partial response (PR)
- Progressive disease (PD)
- Stable disease (SD)

ORR (best response of CR and PR) will be descriptively analyzed and a 95% Clopper -Pearson confidence interval will be calculated.

DCR and CBR will be analyzed descriptively, and numbers and percentages will be reported.

Waterfall plots will be provided for evaluable patients, including plots of best relative change from baseline in lesions, with non-target information and new lesion information, and diagnostic and dose group information.

A listing of assessment results and best responses will be provided for each patient at each tumor assessment visit.

8.6.2 Progression-Free Survival (PFS), Duration of Response (DoR) and Overall Survival (OS)

Survival analyses will be performed for PFS, DoR, and OS.

The number of events and censors, the median and the 95% confidence interval calculated by the Kaplan-Meier method will be reported for each variable. The Kaplan-Meier survival curve will be plotted.

Listings of PFS, DoR and OS for each patient will be provided. The event starts time, event occurrence time, censoring (yes/no) and censoring time for each variable will be reported.

[redacted]

8.7 Safety Analysis

All safety analyses will be based on the Safety Set (SS) unless otherwise specified.

8.7.1 Dose-limiting Toxicity (DLT)

The DLT analysis will only be applied to Phase Ia dose escalation part and based on the DLT Analysis Set.

DLT adverse events will be recorded on the "Adverse Events" of the CRF page. The number and percentage of subjects with adverse events marked as DLT will be reported and classified by SOC and PT.

All adverse events marked as DLT will be presented in the listings.

8.7.2 Adverse Events

Adverse events will be coded using the Medical Dictionary for Regulatory Activities (MedDRA) version 21.1 or higher.

Treatment-emergent adverse events (TEAEs) are defined as adverse events that occur between the first dose and the last dose + 37 days or present at baseline but worsen after dosing. Treatment-emergent serious adverse events (TESAEs) are also subject to this definition. If a date is missing or partially missing, an adverse event will be considered treatment-emergent unless there is clear evidence (by comparison of partial dates) that the adverse event started before the first dose of study treatment or more than 37 days after the last dose of study medication. Specific handling of missing dates is provided in Appendix 1.

Non-TEAEs will not be summarized and will only be presented in listings.

The number of subjects will summarize in TEAEs tables. Each subject will be counted only once for each SOC category and PT category, even if the subject reported one or more events under each subcategory. Unless otherwise specified, AEs tables will be sorted in descending order by the following rule: first by the number of subjects under SOC, then by SOC PT throughout; if the frequency is the same, then by SOC and SOC PT alphabetically.

In the summary of CTCAE grades, the worst CTCAE grade recorded under each SOC or PT group will be summarized for each subject. Similarly, in the summary of the relationship to the study medication, the most severe relationship (related to the study medication) recorded under each SOC or PT group will be summarized for each subject. If the CTCAE grade is missing, the medical review should be performed to supplement the appropriate grade before summary analysis. If the relationship is missing, it will be classified as "related".

The AEs recorded on the "AEs" page of the CRF are newly identified AEs, while the AEs recorded on the "AE follow-up" page are subsequent changes of the AE. Only AEs recorded on the "AEs" page will be counted for event analyses.

Changes in CTCAE grade and outcome of AEs should be recorded on the "AEs follow-up" page. In the analysis of AEs, only the record with the highest CTCAE grade and the most severe outcome for AEs will be included in the analysis.

The following summaries will be provided separately for TEAEs and TESAEs:

- The number of TEAEs, the number and percentage of subjects, and the occurrence of TEAEs by SOC and PT.
- The number of TEAEs, the number and percentage of subjects, and the occurrence of TEAEs by CTCAE grade, SOC, and PT.
- The number of TEAEs related to the study medication, the number and percentage of subjects, and the occurrence of TEAEs by SOC and PT.
- The number of TEAEs related to the study medication, the number and percentage of subjects, and the occurrence of study drug-related TEAEs by CTCAE grade, SOC and PT.

In addition, the following summaries will be provided:

- The number of TEAEs with CTCAE Grade ≥ 3, the number and percentage of subjects, and the occurrence of TEAEs by relationship to the study medication, SOC and PT.
- The number of TEAEs leading to death (CRF page Outcome = "Death"), the number and percentage of subjects, and the occurrence of TEAEs by relationship to the study medication, SOC and PT.
- The number of TEAEs leading to permanent discontinuation of the study medication (CRF page Action taken with study medication = "Study Medication Permanently Discontinued"), the number and percentage of subjects, and the occurrence of AEs by relationship to the study medication, SOC and PT.
- The number of TEAEs leading to temporary interruption of the study medication (CRF page Action taken with study medication = "Study Medication Temporary Interrupted"), the number and percentage of subjects, and the occurrence of AEs by relationship to the study medication, SOC and PT.

- The number of TEAEs leading to study medication dose reduction (CRF page Action taken with study medication = "Study Medication Dose Reduced"), the number and percentage of subjects, and the occurrence of AEs by relationship to the study medication, SOC and PT.
- TEAEs with an incidence of ≥ 10%, the number and percentage of subjects, and the occurrence of AEs by relationship to the study medication, SOC and PT.
- TEAEs with an incidence of ≥ 5% and CTCAE Grade ≥ 3, the number and percentage of subjects, and the occurrence of AEs by relationship to the study medication, SOC and PT.

Listings of AEs, TEAEs and SAEs will be provided separately. The listings will include subject ID, age, gender, dose level, adverse events (SOC, PT and report name), start date, end date, CTCAE grade, relationship to the study medication, action is taken, outcome, serious adverse event or not, serious adverse event criteria.

All death information will be presented in listings.

8.7.3 Laboratory Tests

Laboratory tests include hematology, blood biochemistry, urinalysis, coagulation function analysis, blood glucose, serum amylase and serum lipase.

Laboratory tests will be summarized and analyzed descriptively by visit. The change from the baseline to the visit will be calculated for continuous variables. If multiple records occur at a visit, the last assessment recorded at each visit will be summarized. For summaries of visit cross-tabulations (e.g., maximum post-baseline), scheduled, unscheduled and repeated assessments will be considered.

For observations outside the clinical reference range, abnormal values below the lower limit of the clinical reference range will be indicated by "L" and values above the upper limit of the clinical reference range will be indicated by "H". Values outside the clinical reference range will be assessed by the investigator for clinical abnormalities and will be reported as abnormal not clinically significant (NCS) or abnormal clinically significant (CS). The investigator will record abnormal clinically significant laboratory values as AEs.

Reports as "< X" indicates an assessment record below the lower limit of quantification (BLQ), or "> X" indicates an assessment record above the upper limit of quantification (ULQ), X will be used for the summary analysis, but in the listings, it will be provided as "< X" or "> X".

Changes in laboratory test results will be graded according to the NCI CTCAE version 4.03 criteria (Appendix 2).

The following summaries will be provided for laboratory tests:

- A cross-tabulation of investigator assessment versus baseline for each laboratory result will be summarized by treatment group and visit.

- The NCI CTCAE version 4.03 criteria grades for each laboratory result will be summarized by treatment group and visit. The maximum toxicity grade that occurs during the study will also be summarized.

Abnormal laboratory data will be listed in listings, including subject ID, age, gender, test date, laboratory test classification, laboratory test items, results, reference range, abnormal value flags, and investigator assessment.

8.7.4 Other Safety Tests

Other safety tests include vital signs and ECOG score, physical examination, cardiac color Doppler ultrasound or MUGA, electrocardiogram and pregnancy test. If multiple records occur at a visit, the last assessment recorded at each visit will be summarized. For tables and figures summarized by visit, only scheduled visits will be included in the analysis, but for the worst post-baseline summary tables, both scheduled and unscheduled visits should be included.

Descriptive statistics will be performed for safety test variables. The following summaries will be provided:

- A cross-tabulation of investigator assessment versus baseline for each safety test will be summarized by treatment group and visit (if appropriate).
- If no investigator assessment is available, a cross-tabulation of the medical classification versus baseline for each safety test result will be summarized by treatment group and visit.

Abnormal safety data will be listed in listings, including subject ID, age, gender, test date, test items, results, reference range (if applicable), abnormal value flags (if applicable) and investigator assessment (if applicable).

Pregnancy tests will not be summarized and will only be presented in listings.

8.7.5 Medication Information and Treatment Compliance

Medication information and treatment compliance will be analyzed based on the Safety Set (SS).

Descriptive statistics will be performed for the following variables:

- Actual duration of medication (weeks) = (date of last medication-date of first medication + 1)/7
- The actual total dose (mg) is the sum of the doses taken during a specific period.

  The total dose will be summarized by period:

  - Total dose: the sum of the doses taken during all periods
  - Single dose phase in phase Ia
  - Multiple doses phase in phase Ia (total dose, dose in each cycle)
  - [redacted]

[redacted]

- Medication compliance (%) = [actual total dose (mg) in a given period/planned dose (mg) in a given period] × 100%. Medication compliance will also be summarized by period, the same as the total dose group.

  Planned dose (mg) in a given period = dose (mg) for the subject in the dose group × number of days in that period

  The dose group to which the subject belongs is recorded on the CRF page "Study Entry".

  Days in the period = last day of the period-first day of the period + 1.

8.7.6 Prior and Concomitant Medications

The start and end dates will be compared to the date of the first dose to separate medications into prior only and concomitant medications. Medications that start after the completion/discontinuation date will be listed but not categorized or summarized.

Medications that started and stopped prior to the first dose will be classified as prior medication. Concomitant medications refer to medications that start before the first dose and are discontinued on or after the date of the first dose. Concomitant medications also refer to medications that started on or after the date of the first dose. Medications that started after the last dose of the study medication did not fall into these categories.

Missing data will be categorized as described in Appendix 1.

Prior and concomitant medications will be summarized in the full analysis set. The frequency tables for medications will be provided and coded by the Anatomical Therapeutic Chemical (ATC) drug class and the World Health Organization Drug Dictionary Enhanced (WHO-DDE, version September 2018 or latest) (ATC1, ATC3, and PT).

8.8 Additional Analysis

8.8.1 PK Analysis

The PK analysis will be provided by Shanghai Mosim Pharmaceutical Technology Co., Ltd.

8.8.2 Biomarker Analysis

The analysis will be described in a separate analysis plan.

9. Analyses Different From those Described in the Study Protocol

Subgroup analysis grouping is changed from "> 65 years or < 65 years" to "> 65 years or ≤ 65 years".

For the analysis set, the full analysis set is changed from "used for efficacy evaluation" to "used for baseline data analysis". The evaluable analysis set is used for efficacy analyses.

10. References

Clopper CJ and Pearson ES. The use of confidence or fiducial limits illustrated in the case of the binomial, *Biometrika*. 1934; 26(4):404-413.

11. Appendices

Appendix 1.Handling of Dates, Incomplete Dates and Missing Dates for Adverse Events and Concomitant Medications

The following data will be imputed using the following algorithm:

- Adverse Events
- Concomitant Medications

Table 1.Imputation Rules for Partial or Missing Start Dates

		Stop Date						
		Complete: *yyyymmdd*		Partial: *yyyymm*		Partial: *yyyy*		
Start Date		**< 1st dose**	**≥ 1st dose**	**< 1st dose *yyyymm***	**≥ 1st dose *yyyymm***	**< 1st dose *yyyy***	**≥ 1st dose *yyyy***	missing
Partial: *yyyymm*	**= 1st dose** *yyyymm*	2	1	2	1	n/a	1	1
	**≠ 1st dose** *yyyymm*		2		2	2	2	2
Partial: *yyyy*	**= 1st dose *yyyy***	3	1	3	1	n/a	1	1
	**≠ 1st dose *yyyy***		3		3	3	3	3
Missing		4	1	4	1	4	1	1

1 = Impute the date of first dose
2 = Impute the first of the month
3 = Impute January 1 of the year
4 = Impute January 1 of the stop year

Note: If the start date imputation leads to a start date after the stop date, do not impute the start date.

Imputation rules for partial or missing stop dates:

Initial imputation

- For partial stop date mmyyyy, impute the last of the month.
- For partial stop date yyyy, impute December 31 of the year.
- For completely missing stop date, do not impute.
- If the stop date imputation leads to a stop date after the death date, then impute the stop date as the death date.
- If the stop date imputation leads to a stop date that is before the start date, then there is a data error and do not impute the stop date. (ie. set the stop date as missing).

Imputation rules for partial or missing death dates:

- If death year and month are available but the day is missing:
- If mmyyyy for last contact date = mmyyyy for death date, set death date to the day after the last contact date.
- If mmyyyy for last contact date < mmyyyy for death date, set death date to the first day of the death month.
- If mmyyyy for last contact date > mmyyyy for death date, data error and do not impute.
- If both month and day are missing for death date or a death date is totally missing, do not impute.

Appendix 2.Handling of Dates, Incomplete Dates and Missing Dates for Adverse Events and Concomitant Medications

Laboratory Values

Safety laboratory values below a distinct limit (e.g. detection limit, documented as "< [limit]") will be substituted by half of the limit and values above a distinct limit (documented as "> [limit]") will be substituted by the limit itself for all analyses.

A Grade (based on CTC AE version 4.0 [v4.03: June 14, 2010]) will be assigned to each laboratory result as detailed in Table 2. Depending on the toxicity definition, the same result may be assigned to two grading for deviations towards higher or lower values. In case no lower limit of normal is provided for the absolute lymphocyte, neutrophils or leukocyte counts it will not be differentiated between grade 1 and grade 0 results for these parameters. Values not meeting any of the criteria will be assigned a grade 0.

Table 2. Grading of Select Laboratory Parameters

Laboratory Parameter [Unit]	Grade 1	Grade 2	Grade 3	Grade 4
Lymphocytes [G/L]	0.8 - < LLN	0.5 - < 0.8	0.2 - < 0.5	< 0.2
Neutrophils [G/L]	1.5 - < LLN	1.0 - < 1.5	0.5 - < 1.0	< 0.5
Leukocytes [G/L]	3.0 - < LLN	2.0 - < 3.0	1.0 - < 2.0	< 1.0
Platelets [G/L]	75 - < LLN	50 - < 75	25 - < 50	< 25
Hemoglobin [g/L]*	100 - < LLN	80 - < 100	65 - < 80	< 65
Albumin [g/L]	30 - < LLN	20 - < 30	< 20	not defined
AST*	> ULN – 3*ULN	> 3*ULN – 5*ULN	> 5*ULN – 20*ULN	> 20*ULN
ALT *	> ULN – 3*ULN	> 3*ULN – 5*ULN	> 5*ULN – 20*ULN	> 20*ULN
GGT	> ULN – 2.5*ULN	>2.5*ULN – 5*ULN	> 5*ULN – 20*ULN	> 20*ULN
Bilirubin	> ULN – 1.5*ULN	>1.5*ULN – 3*ULN	> 3*ULN – 10*ULN	> 10*ULN
Fibrinogen^	%change of BL <25% or 0.75*LLN - < LLN	25%- <50% of BL or < 75*LLN – 0.5*LLN	50% - <75% of BL or < 0.5* LLN – 0.25*LLN	>= 75% of BL or < 50mg/dL or < 0.25*LLN
Calcium [mmol/L]*	2.0 - < LLN	1.75 - < 2.0	1.5 - < 1.75	< 1.5
Potassium [mmol/L]*	not defined	3.0 - < LLN	2.5 - < 3.0	< 2.5
Lipase	> ULN – 1.5*ULN	> 1.5*ULN – 2.0*ULN	> 2.0*ULN – 5.0*ULN	> 5.0*ULN

Laboratory Parameter [Unit]	Grade 1	Grade 2	Grade 3	Grade 4
Amylase	> ULN – 1.5*ULN	> 1.5*ULN – 2.0*ULN	> 2.0*ULN – 5.0*ULN	> 5.0*ULN
Hyperglycemia [mmol/L]	> ULN – 8.9	> 8.9 – 13.9	> 13.9 – 27.8	> 27.8
Glucosuria	Present	-	-	-
Proteinuria#	+ proteinuria or urinary protein ≥ULN - < 1.0 g/24 hrs	≥2+ proteinuria or urinary protein 1.0 - < 3.5 g/24 hrs	-	-
INR increased	>1.2-1.5 or >1 - 1.5*BL if on anticoagulation	>1.5-2.5 or >1.5 – 2.5 * BL if on anticoagulation	>2.5 or >2.5 * BL if on anticoagulation or bleeding	-
Activated partial thromboplastin time prolonged	>ULN – 1.5 * ULN	>1.5 – 2.5 * ULN	> 2.5 * ULN or bleeding	-

BL: baseline value, LLN: Lower limit of normal, ULN: Upper limit of normal
*: Clinical criteria from CTC AE 4.0 grading were not considered in order to assign grades
^: In case of conflicting criteria the higher grade will be assigned, % change only used when baseline is <LLN
#: in case of conflicting criteria the higher grade will be assigned.
