# Supplementary material for: First-in-human phase Ia study of the PI3Kα inhibitor CYH33 in patients with solid tumors
Source: Nat Commun. 2022 Nov 16;13:7012. doi: 10.1038/s41467-022-34782-9 (PMC9669016; doi:10.1038/s41467-022-34782-9)
Supplement: Supplementary file 2 — Reporting Summary [file 41467_2022_34782_MOESM2_ESM.pdf]

nature portfolio

Corresponding author(s): Rui-Hua Xu
Last updated by author(s): Oct 10, 2022

Reporting Summary

Nature Portfolio wishes to improve the reproducibility of the work that we publish. This form provides structure for consistency and transparency in reporting. For further information on Nature Portfolio policies, see our Editorial Policies and the Editorial Policy Checklist.

Statistics

For all statistical analyses, confirm that the following items are present in the figure legend, table legend, main text, or Methods section.

n/a	Confirmed	
☐	☒	The exact sample size (n) for each experimental group/condition, given as a discrete number and unit of measurement
☒	☐	A statement on whether measurements were taken from distinct samples or whether the same sample was measured repeatedly
☒	☐	The statistical test(s) used AND whether they are one- or two-sided<br>*Only common tests should be described solely by name; describe more complex techniques in the Methods section.*
☒	☐	A description of all covariates tested
☒	☐	A description of any assumptions or corrections, such as tests of normality and adjustment for multiple comparisons
☐	☒	A full description of the statistical parameters including central tendency (e.g. means) or other basic estimates (e.g. regression coefficient) AND variation (e.g. standard deviation) or associated estimates of uncertainty (e.g. confidence intervals)
☒	☐	For null hypothesis testing, the test statistic (e.g. F, t, r) with confidence intervals, effect sizes, degrees of freedom and P value noted<br>*Give P values as exact values whenever suitable.*
☒	☐	For Bayesian analysis, information on the choice of priors and Markov chain Monte Carlo settings
☒	☐	For hierarchical and complex designs, identification of the appropriate level for tests and full reporting of outcomes
☐	☒	Estimates of effect sizes (e.g. Cohen's d, Pearson's r), indicating how they were calculated

Our web collection on statistics for biologists contains articles on many of the points above.

Software and code

Policy information about availability of computer code

Data collection	All clinical data were collected on Electronic Case Report Form (eCRF) designed using Medidata Classic Rave.
Data analysis	PK parameters were calculated by the Phoenix Winnonlin software (v8.3), statistical analyses were performed using SAS software v9.3 (Cary, North Carolina, USA).

For manuscripts utilizing custom algorithms or software that are central to the research but not yet described in published literature, software must be made available to editors and reviewers. We strongly encourage code deposition in a community repository (e.g. GitHub). See the Nature Portfolio guidelines for submitting code & software for further information.

Data

Policy information about availability of data

All manuscripts must include a data availability statement. This statement should provide the following information, where applicable:

- Accession codes, unique identifiers, or web links for publicly available datasets
- A description of any restrictions on data availability
- For clinical datasets or third party data, please ensure that the statement adheres to our policy

The study protocol and the statistical analysis plan are available as Supplementary Note 1 and Note 2 in the Supplementary Information file. Clinical data are not publicly available due to involving patient privacy, but can be accessed on request from the corresponding author Rui-Hua Xu for 10 years; individual de-identified participant data will be shared. The remaining data are available within the Article, Supplementary Information or Source Data file. Source data are provided with this paper.

Human research participants

Policy information about studies involving human research participants and Sex and Gender in Research.

Reporting on sex and gender	This is a phase I clinical trial, which included patients with advanced solid tumors who had failed standard treatment. Both male and female patients could be enrolled in the study. Due to the small sample size, sex and gender were not divided into groups for analysis.
Population characteristics	51 patients (median age, 54 years) including 36 (70.6%) patients with PIK3CA mutations were enrolled. Of these patients, Of these patients, 19 patients were recruited to the dose-escalation stage, with 15 (78.9%) patients having unknown PIK3CA mutation status and 4 (21.1%) patients harboring PIK3CA mutations. Thirty-two (100%) patients harboring PIK3CA mutations determined via local laboratory testing were included in the dose-expansion stage, 4 patients of them in the 20 mg group, 12 in the 30 mg group, and 16 in the 40 mg dose group. At baseline, a total of 18 (35.3%) patients had an Eastern Cooperative Oncology Group (ECOG) score of 0 and 33 (64.7%) patients had a score of 1. The primary tumor types were categorized as colorectal cancer (n = 10, 19.6%), breast cancer (n = 6, 11.8%), ovarian cancer (n = 6, 11.8%), cervical cancer (n = 6, 11.8%), endometrial cancer (n = 5, 9.8%), nasopharyngeal carcinoma (n = 5, 9.8%), urinary system neoplasm (n = 4, 7.8%), and other cancer types (n = 9, 17.6%). Of these 51 patients, 19 (37.3%) had previously received first-line systemic therapy, 12 (23.5%) had previously received second-line systemic therapy, and 20 (39.2%) had been heavily treated with at least 3 lines of systemic therapy.
Recruitment	The site used their existing patient population to determine which subjects meet the inclusion/exclusion criteria and then invited them to review the information consent form. All eligible subjects were invited to participate in this study. Potential self-selection bias into clinical trial may occur with the most interested and keen willing to participate rather than those uninterested, and there is no way to eliminate this bias as with all clinical trials. However this potential self-election bias is unlikely to impact results. There were no other biases as the inclusion and exclusion criteria were robust.
Ethics oversight	Institutional Review Board of Sun Yat-sen University Cancer Center, China.

Note that full information on the approval of the study protocol must also be provided in the manuscript.

Field-specific reporting

Please select the one below that is the best fit for your research. If you are not sure, read the appropriate sections before making your selection.

☒ Life sciences ☐ Behavioural & social sciences ☐ Ecological, evolutionary & environmental sciences

For a reference copy of the document with all sections, see nature.com/documents/nr-reporting-summary-flat.pdf

Life sciences study design

All studies must disclose on these points even when the disclosure is negative.

Sample size	Basing on ATD + mTPI2 design, we conducted dose escalation and dose expansion to determine the MTD and RP2D. SMC will evaluate the safety, efficacy data from the previous dose group and refer to existing PK data, and determine the dose and number of subjects in the next dose group. It is expected that approximately 60 patients will be enrolled in Phase Ia. The actual number of subjects and dose escalation level will be discussed and decided by SMC based on the obtained safety data and/or PK/PD modeling simulation data.
Data exclusions	No data was excluded from analyses.
Replication	Clinical trial data was obtained at the time of each visit, hence it was not replicated. PK (pharmacokinetic)/PD (pharmacodynamic) analysis done on blood samples or biopsies were performed on a single sample due to insufficient material.
Randomization	No randomization. This was a single-arm trial with no control arm. Therefore, this study did not involve randomization.

Blinding	Not applicable for patients, as it was an open label trial.

Reporting for specific materials, systems and methods

We require information from authors about some types of materials, experimental systems and methods used in many studies. Here, indicate whether each material, system or method listed is relevant to your study. If you are not sure if a list item applies to your research, read the appropriate section before selecting a response.

Materials & experimental systems

n/a	Involved in the study
☒	☐ Antibodies
☒	☐ Eukaryotic cell lines
☒	☐ Palaeontology and archaeology
☒	☐ Animals and other organisms
☐	☒ Clinical data
☒	☐ Dual use research of concern

Methods

n/a	Involved in the study
☒	☐ ChIP-seq
☒	☐ Flow cytometry
☒	☐ MRI-based neuroimaging

Clinical data

Policy information about clinical studies

All manuscripts should comply with the ICMJEguidelines for publication of clinical research and a completedCONSORT checklist must be included with all submissions.

Clinical trial registration	ClinicalTrials.gov NCT03544905
Study protocol	Full Protocol included in file uploaded as Supplemental Material.
Data collection	Subject recruitment and data collection were done at Sun Yat-sen University Cancer Center, China. First patient enrolled on July 13,2018, last patient enrolled on March 29,2021. Data cut-off date was July 16, 2021.
Outcomes	Phase Ia primary endpoint: Type and frequency of treatment-emergent adverse events (TEAE), and evaluation of toxicity grades according to NCI CTCAE version 4.03; Laboratory test results, electrocardiogram (ECG) and cardiac imaging findings and physical examination findings (including vital signs, weight and ECOG performance status score), etc. Number and proportion of patients experiencing DLT in the DLT observation phase (approximately 28 days after the first dose, and 35 days after the first dose for patients receiving a single administration) (Phase Ia dose escalation part and Phase Ia dose expansion part). Maximum tolerated dose (MTD). If MTD is not observed, the RP2D will be determined through PK (pharmacokinetic)/PD (pharmacodynamic) data, safety and preliminary efficacy.<br>Phase Ia secondary endpoint: Response assessment endpoints: objective response rate (ORR), progression-free survival (PFS), duration of response (DoR) and disease control rate (DCR), clinical benefit rate (CBR). PK parameters of CYH33 and its metabolite (I27), including AUC0 last, AUC0-24h, AUC0-∞, Cmax, tmax, t1/2, Vz/F, CL/F, ARCmax, ARAUC 0-24h, LI.
